# Molecular imaging with nanoparticles: the dwarf actors revisited 10 years later

**DOI:** 10.1007/s00418-018-1753-y

**Published:** 2018-11-16

**Authors:** Gudrun C. Thurner, Paul Debbage

**Affiliations:** 1 Department of Radiology, Innsbruck Medical University, 6020 Innsbruck, Austria; 2 Division of Histology and Embryology, Department of Anatomy, Medical University Innsbruck, Muellerstrasse 59, 6020 Innsbruck, Austria

**Keywords:** Nanotechnology, Nanomedicine, Quantum nanoparticles, Targeting, Near infrared, Translation

## Abstract

We explore present-day trends and challenges in nanomedicine. Creativity in the laboratories continues: the published literature on novel nanoparticles is now vast. Nanoagents are discussed here which are composed entirely of strongly photoluminescent materials, tunable to desired optical properties and of inherently low toxicity. We focus on “quantum nanoparticles” prepared from allotropes of carbon. The principles behind strong, tunable photoluminescence are quantum mechanical: we present them in simple outline. The major industries racing to develop these materials can offer significant technical guidance to nanomedicine, which could help to custom-design strongly signalling nanoagents specifically for stated clinical applications. Since such agents are small, they can be targeted easily, making active targeting possible. We consider it timely now to study the interactions nanoparticles undergo with tissue components in living animals and to learn to understand and overcome the numerous barriers the organism interposes between the blood and targets in or on parenchymal cells. As the near infra-red spectrum opens up, detection of glowing nanoparticles several centimeters deep in a living human subject becomes calculable and we present a simple way to do this. Finally, we discuss the slow-fuse and resource-inefficient entry of nanoparticles into clinical application. A first possible reason is failure to target across the body’s barriers, see above. Second, in the sparse translational landscape funding and support gaps yawn widely between academic research and subsequent development. We consider the agendas of the numerous “stakeholders” participating in this sad landscape and point to some faint glimmers of hope for the future.

## Introduction

An astonishing number of the great physicists have also made major advances in nanotechnology, and essentially, all of their contributions are in thousandfold daily use in 2018, providing exact calculation powering major industrial enterprises in which urgent hopes are embodied, such as more efficient solar energy panels and, also, brighter flat-screen technologies. Thus, these giants mingle today with the dwarf actors that we reviewed 10 years ago (Debbage and Jaschke 2008). It is now almost 60 years, since Feynmann’s lecture mooted the possibility of nanotechnology (Feynman et al. 1964), and about one quarter of the time that has elapsed, since the first liposomal nanoparticles were reported. It is time to revisit the dwarf halls and assess progress. As a touchstone to aid assessment, we will place centrally a question that is of high clinical relevance today. Our question posits a local density of nanoparticles anchored to a location 3 cm deep within a human body and emitting infrared radiation in the “water windows” (Zhu et al. 2018b). With what intensity must this collection of nanoparticles emit light to trigger a detector that requires a signal-to-noise ratio > 3 to register the presence of the nanoparticles against the background of the body’s infrared background noise? Can we design this nanoparticle using materials satisfactory to a regulatory authority that acts as Maxwell’s Demon at the gateway to the clinical marketplace?

This review will not re-examine the topics covered in our earlier review (Debbage and Jaschke 2008). The field of nanotechnology in Medicine has become vast, with many thousands of publications even in restricted areas of the field. Furthermore, there have been singular developments in one area which point the way forward to rational (customized) design to specification, providing a conceptual framework for the creation of economical and effective nanoagents for biomedical use. Some major areas of progress will, therefore, feature only as sidelines in this review, because they cannot be relevant to the examination of a living human being. This includes the promising field of upconversion, for example. As will become evident by the end of the first part of this review, there are also reasons to sideline major types of nanoparticle that have experienced flourishing development recently, but are unlikely to compete on regulatory grounds with the allotropes of carbon; we, therefore, only note in passing the excellent qualities of noble metal nanocages and of silicon-based nanoparticles.

This review will also largely ignore the toxicological aspects of nanoparticles. The translational and in particular the regulatory processes subsume these aspects routinely and as such can be built from the start into the rational design process used to create customized nanoparticles. Otherwise stated: the regulatory aspects can be planned in from the beginning and the design process then automatically avoids use of materials or energies that will be the objects of regulatory aversion or caprice.

The non-invasive examination of a human body has typically depended on high-energy materials and energies, for example, in Nuclear Medicine. The field of targeted imaging has been represented best by Nuclear Medicine, which can use extremely minimal nanoagents, because they emit hugely energetic signals. Magnetic resonance imaging, an important area of interest in imaging with nanoparticles, also uses intense energies, imaging within large magnetic fields. However, as progress has generated materials that can be visualized by use of much lower energies in the form of photoluminescence, it has recently become timely to consider the possibilities now opening up to peer a few centimeters into the body with the expectation of reliably detecting lesions by use of simple and cheap equipment after application of non-toxic energies and chemistries. We note the potential use of photoacoustic imaging to detect nanoparticles excited by infrared wavelengths (Zhang and Yu 2015; Zhang et al. 2013), but this requires relatively expensive equipment and we will not follow this theme further here. This review focuses on the generation of light at the specific localities of lesions. We are aiming to view the coloured lamps being used in the dwarf halls, a field known earlier in a simpler form as fluorescence.

**Fig. 1:**
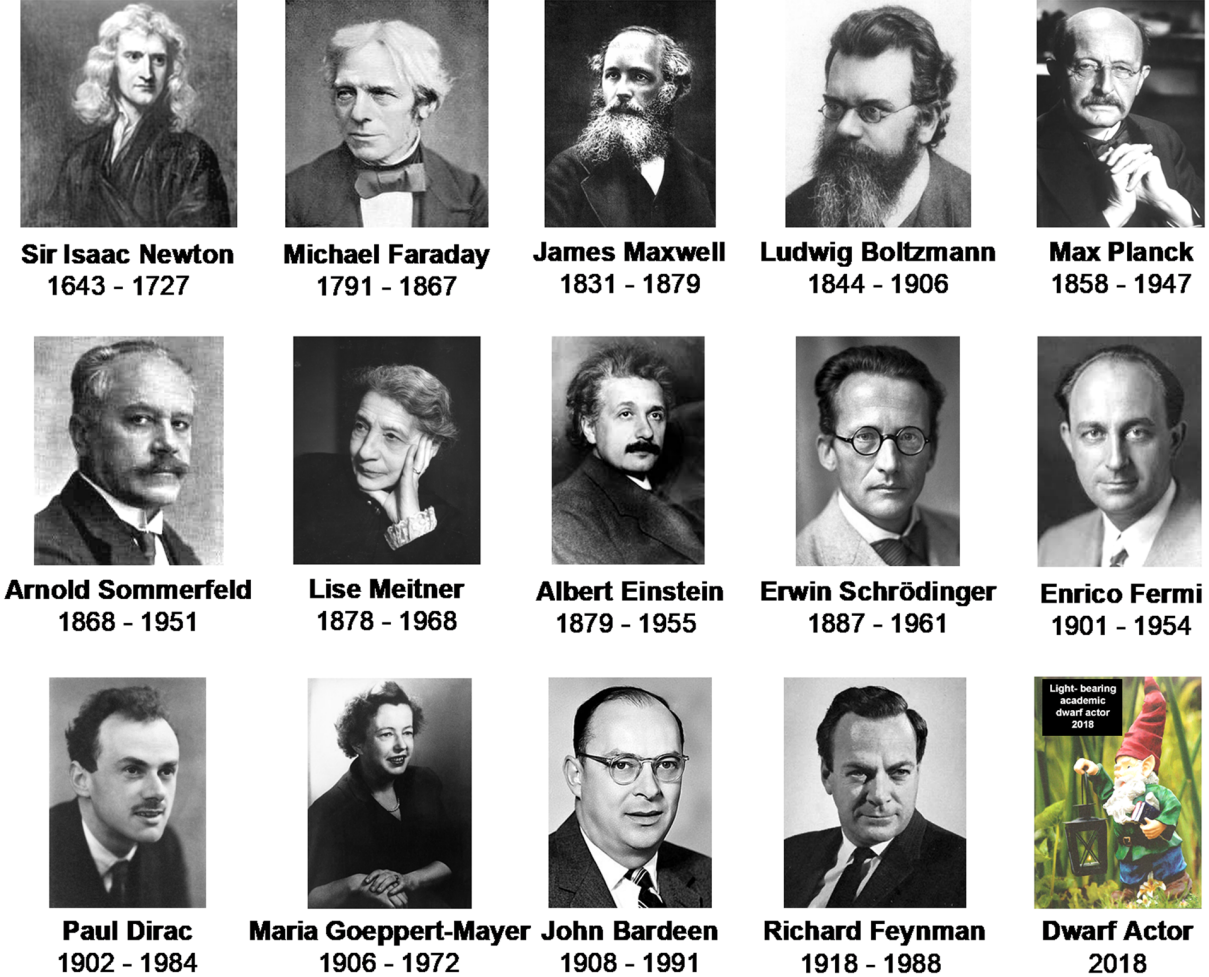
Giants in both physics and nanomedicine. Each of these 11 giants made major contributions in understanding matter and energy at the nanoscale and below. Each of their contributions is used daily today in academic and industrial research and development. Every worker in nanomedicine benefits from these men and women, often unknowingly. The final image is colored, because it represents hope for the future. We salute here all postdoctoral non-tenured workers in nanomedicine: we hope that amongst them today there is at least one Giant

The physics of matter and energy culminated 80 years ago in Quantum Mechanics, which describes the interactions of matter and energy with unprecedented precision and accuracy. Some of the Giants who promoted research that would lead to Quantum Mechanics (e.g., Faraday, Maxwell) or who promulgated concepts and research directions that depend on it (e.g., Sommerfeld, Feynmann) were active participants not only in the physics but also in the nanosciences. We honour some of them here (Fig. 1). These giants are not fading into the past. Their inspirations and their contributions are today in daily use throughout the world of nanotechnology, which today embraces nanomedicine but also has numerous research directions in materials science, photovoltaics, communications technology at several different levels, food technology, and many others. Most aspects of photoluminescence today cannot be discussed without appealing to concepts that are unavoidably and essentially quantum mechanical; we will present briefly one of these, namely, the extreme mobility of electrons densely packed in crystal solids. The rational design of glowing nanomaterials now requires some essential minimal familiarity with matter and energy as described in modern terms: a passing acquaintance with one or two non-integrated concepts from the general field of quantum mechanics no longer suffices for research at the cutting edge. We, therefore, preface the classical topics treated in this review by short visits to the modern concepts underlying research in the production of glowing nanoagents. Unavoidably abbreviated and simplified, our treatment of these crucial matters aims to provide a first step towards a minimal familiarity with the topic, to sharpen our view of the remarkable principles governing the dwarf lights.

The review has five parts, the first describes the quantum–mechanical background to the energies and materials relevant to the nanoparticle design, construction and function. The second part describes a few kinds of quantum nanoparticles and assesses their potential usefulness in functioning to provide imaging in the NIR windows 1 and 2. These two parts arrive at the conclusion that rational design of nanoparticles for clinical use will almost certainly eventually be based on quantum nanoparticles and—with good likelihood—they will be based on carbon allotropes. The third part looks at the quantitative aspects involved in designing nanoparticles for lesion niches, “horses for courses”. Part Four looks at the next grand challenge in nanomedicine, which is to overcome the minimum number of the ~ 1 × 10^20^ barriers that exist and are highly effective in a living human body. The final part of the review looks at the translational landscape of nanomedicine.

The review assesses possible approaches to developing a histochemistry of the living human body, known currently as molecular imaging.

Vision in the world of glowing particles is obscured by irrelevant “signals” in the form of autofluorescence, in the blue–green–yellow regions of the spectrum from the components of proteins and some small molecules. In addition, in the longer wavelengths of the near infrared there are bond vibrations that cause background noise. In addition, the light waves carrying true signals are easily scattered, both by microscopically small structures such as cell nuclei and also by macroscopic tissue components such as blood vessels containing blood components, and by connective tissue proteins and fatty tissues. In general, it is desirable to work with wavelengths of light that “ignore” the body tissues and chemistry but leave the glowing nanoagents clearly visible. The two water windows in the near-infrared spectrum are beginning to offer this opportunity.

Tissues have minimal light absorbance in the two wavelength ranges 650–900 nm (NIR window I) and 1000–1450 nm (NIR window II) (Loo et al. 2004; Li et al. 2011a, b, 2012a, b, c; Wang and Zhang 2014; Chu et al. 2014; Tang et al. 2012; Lim et al. 2006). For small animals this enables whole animal imaging with high sensitivity in core organs in real time without the need for dissection (e.g., Abdukayum et al. 2013; Cao et al. 2012a). For larger animals and humans the penetration depth of the NIR dyes used earlier, with excitation and emission wavelengths between 700 and 850 nm, is too shallow. These wavelengths can penetrate up to 2 mm (Liu et al. 2013). A rethinking of some earlier working principles leads away from using the maximal absorption peak of a dye for excitation. Instead, the use of long absorption tails in the spectra that lead into the near infrared opens the possibility of using excitation wavelengths above 1000 nm and harvesting signals at wavelengths above 1100 nm (Zhu et al. 2018a, b). Penetration depths up to 10 cm have been reported (Prevo et al. 2008; Weissleder 2001). Advances in the use of dyes earlier available and also of novel dye molecules and quantum–mechanical principles go hand in hand with the development of novel sensor types that detect efficiently in different parts of the near-infrared spectrum which, it should be noticed, is considerably wider than the entire visible spectrum.

**Fig. 2:**
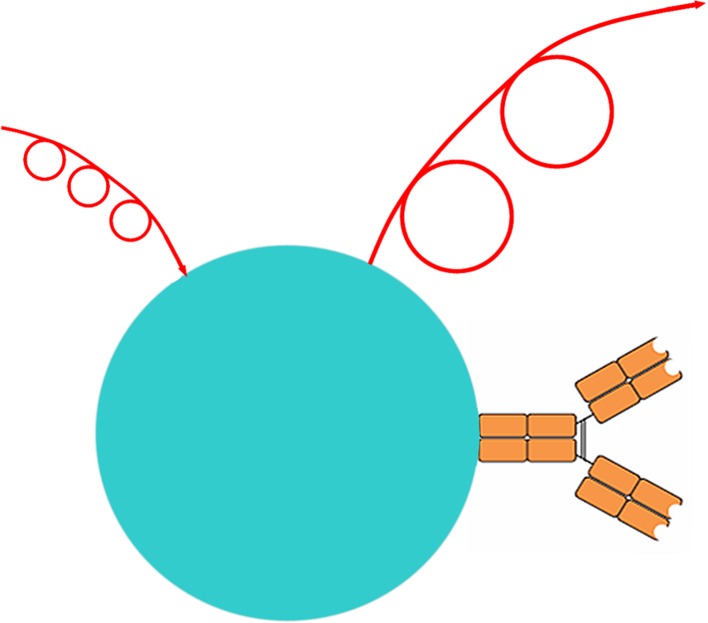
A nanoparticle (matrix colored turquoise) bearing a single antibody molecule as targeting group (at right). When exposed to shorter wave radiation (top left) it emits longer wave radiation (top right). This is the simplest structure which can participate in the answers to the leading question that is in the background at every stage of this review

For simplicity we consider a nanoparticle composed of light-emitting material and bearing a single targeting group (Fig. 2). Anything more complex than this will potentiate both the technical and the regulatory difficulties in moving the nanoparticle towards clinical application.

In general, a photon arises from a single electron (other cases will be encountered below). To obtain the maximum intensity of radiation exiting the skin of the body, it is, therefore, necessary to construct the nanoparticle from a material that makes available a large number of electrons, to maximize extraction of the maximum possible number of photons. The first part of this review, therefore, consists of a brief and much abbreviated statement of the principles involved in the interactions of light with matter. We will use the long-standing division of matter into insulators, semiconductors, and conductors, but the venerable restriction of the word “conductors” to metals will be lifted and we will augment this group by considering not only organic conductors, but also and in particular the allotropes of carbon.

## Energies and materials

### Light

An electric field can do mechanical work. It can push a charged particle through space, exerting a mechanical force of the type first quantified in the 17th century by Isaac Newton and measured in units named for him. The electric field is a force that causes a charged particle to accelerate through space if no opposing forces are in play. Faraday, in the early 19th century, using concepts familiar from earlier studies of fluids, discussed the transfer of electrical force in terms of “lines of force”, noting that these exert mechanical tension and pressure (Faraday 1846; Campbell and Garnett 1882; Hirshfeld 2006; Friedel 1981). Faraday wrote frequently about both magnetism and electricity in terms of lines of force and in 1851 used this concept to define a magnetic field (Faraday 1852a, b). It remained for Maxwell to define the electrical field, in terms very close to Faraday’s definition of the magnetic field (Maxwell 1965). Faraday speculated in 1846 that light is a vibration of electrical and magnetic lines of force transmitted through a medium, and later provided evidence for this by demonstrating that magnets affect the polarization of light rays (Chisholm 1911). Maxwell in the 1860s formulated Faraday’s results as 20 mathematical equations (Giordano 2009; Bergmann 1992; Bais 2005; O’Connor and Robertson 1997) that unify the electric and magnetic forces into a single electromagnetic force. Oliver Heaviside and Heinrich Hertz (1884) used mathematical techniques, not available earlier to Maxwell, to condense the theory into the four equations that we now know as “Maxwell’s equations” which later became leading considerations as Albert Einstein formulated his theory of Special Relativity. At present, 150 years later, intensive research is still discovering new technologies based on Faraday’s and Maxwell’s concepts, as shown by the many texts covering these phenomena in detail, see, for example, (Grant and Phillips 1990; Jackson 1998).

Maxwell’s equations provide a prescription for calculating the fields arising from a given system of charges, and the concept of a field is useful to provide a simplified explanation of permittivity. We discuss permittivity here in terms of field theory, although we noted above that fields, describing continuous entities, are not compatible with quantum theory. Precise description of the interactions of electric fields and matter requires application of quantum mechanics, in particular as formulated by Erwin Schrödinger. Modern calculations apply a range of assumptions based on various models to solve Schrödinger’s equation to obtain highly accurate statements about permittivity.

In the unified theory of electromagnetism the oscillating pair of coupled fields, one electric and one magnetic that together make up a wave of light, travels through empty space at a speed defined exclusively by the “permittivity” and “permeability” of empty space:

$$c_{0}^{2} = \, 1/(\varepsilon_{0} \mu_{0} ),$$

where *c*_0_ is the speed of light in empty space (3 × 10^8^ m/s), *ε*_0_ is the permittivity of empty space, and *μ*_0_ is the permeability of empty space.

Our discussion of how light interacts with matter will explain the concept of permittivity, focusing upon the fact that light includes a rapidly oscillating electric field.

The following text is a simplified, non-mathematical account of light–matter interactions, using the concept of a field. To begin with a familiar example, climbing a hill requires physical muscle-powered work against a gravitational field, which is generated by mass. In the same way, an electrical charge generates an electrical field. The comparison is not merely verbal, as can be seen by comparing the “inverse square” laws developed from each of these concepts:

*Newton’s Law (gravity)* strength of the force *F*_g_ between two interacting point masses:

$$F_{\text{g}} = \, G\left( {m_{1} m_{2} } \right)/r^{2} .$$

*Coulomb’s law (charge)* strength of force *F*_e_ between two interacting point charges:

$$F_{\text{e}} = \, k_{\text{e}} \left( {q_{1} q_{2} } \right)/r^{2} .$$

*Electrical charge is a fundamental property*: It cannot be created or destroyed, though it can be transferred from one body to another. The charge on all electrons is the same and is negative (−), and the positive charge on all protons is the same as that on electrons but with reversed sign (+). Unlike the gravitational field, the electrical field can originate from either a positively or as negatively charged particle. Ordinary matter consists largely of charged particles, their charge being quantized in integral multiples of *e*: a single electron carries one unit of negative charge, −*e*, while a proton carries one unit of positive charge, +*e*. An electric charge produces an electric field everywhere in empty space. If a single charged particle is introduced into an empty space, it will fill the entire space with an electric field. If a second charged particle now enters the space, it too will generate a field; if both particles carry charges of the same sign, the interacting fields will mediate repulsion between the two particles and it will require work to move them closer to one another. If the particles carry charges of opposite sign, it requires work to prevent them moving towards one another. In material bodies, the charges exist in an equilibrium. Atoms, molecules, and crystal lattices consist of clouds of negative charge (electrons) bound to and surrounding positive point-like charges (protons). A neutral atom has no overall charge, but contains positive charge concentrated in the nucleus (radius = 10^−14^ m = 1/100,000 nm) and (an equal amount of) negative charge in the form of an electron cloud (radius = 10^−10^ m = 1/10 nm) surrounding the nucleus. The equilibrium present amongst the charges can be disturbed by the action of an external electric field.

Light consists of an oscillating pair of coupled fields, one electric and one magnetic. The oscillating electric field can interact with the positive and negative charges on the protons and electrons within matter. If electrons are present that are free to move throughout the material, as in metals, the electric field of a light ray impacting the material will interact with these free electrons and causes them to move as an electric current across the material: the material is a “conductor” of electricity. Most materials are, however, not conductors, they are “dielectrics”, which includes not only a narrow class of so-called insulators, but the “broad expanse of non-metals considered from the standpoint of their interaction with electric, magnetic, or electromagnetic fields; dielectrics include gases as well as liquids and solids, and their dielectric properties have to do with their storage of electric and magnetic energy as well as its dissipation” (von Hippel 1954). The word “dielectric”, coined by William Whewell at the request of Faraday, arose from “dia” + “electric”, meaning poorly electric and able to sustain an electric field without passing (much) electric current (Daintith 1994; Frank 1996). In this review, the dielectric/insulating materials under discussion are solids. The charges in them are strongly attached to specific atoms and molecules: the charges are “tightly bound”. In these materials no free or loosely bound electrons are present. Although bound, however, electrons in the outer orbitals of the atoms can be shifted slightly in the space within an atom or a molecule or a crystal lattice. These microscopic shifts account for the characteristic behaviours of dielectric materials, and are, therefore, important for explaining numerous phenomena in electronics, optics, solid-state physics, and cell biophysics.

**Fig. 3:**
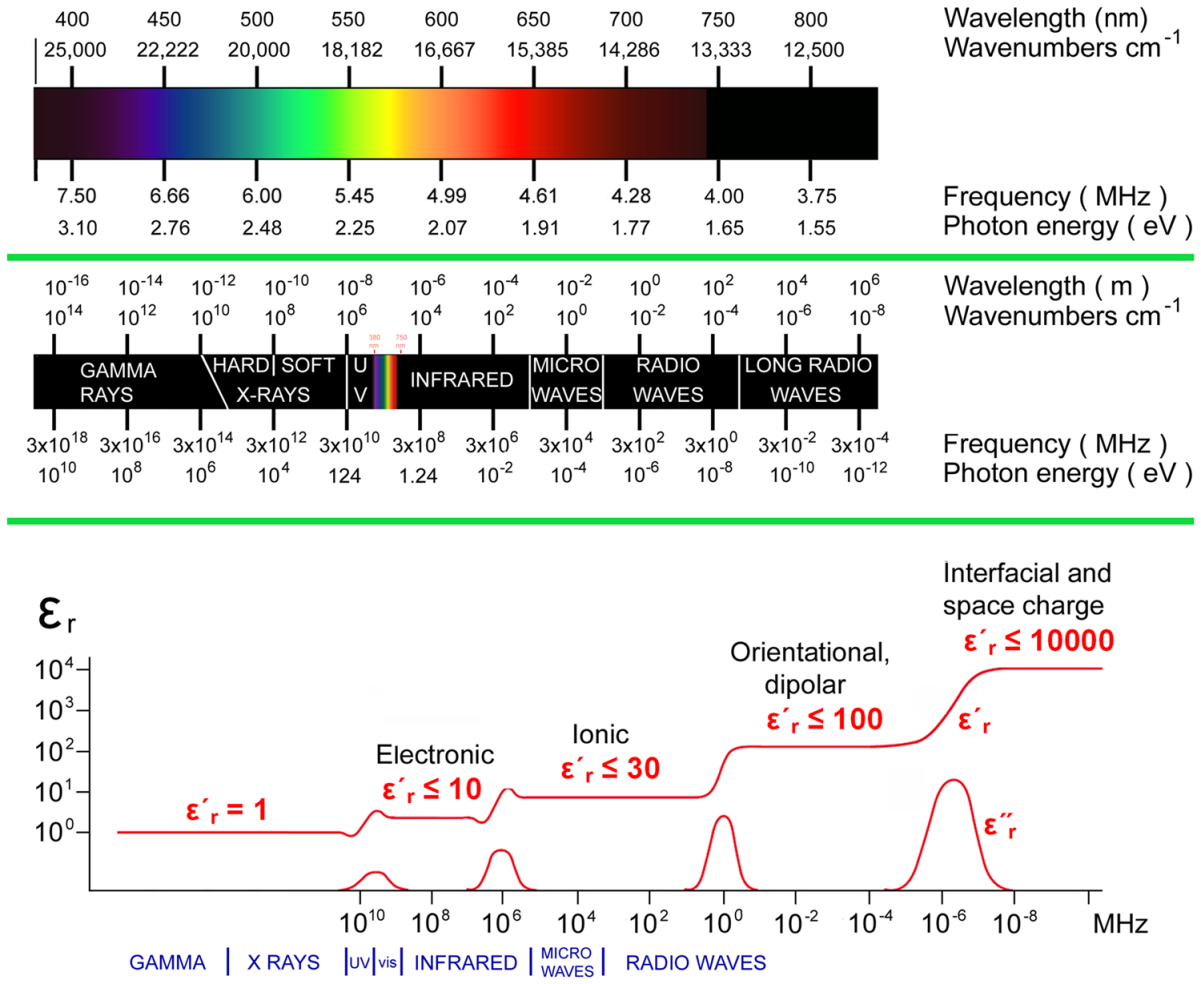
This conversion tool relates the various units employed in different disciplines to describe the properties of light quantitatively. The units are placed in relation to one another and to the colors which the human visual system perceives. Below the rainbow in the upper part of the figure, and drawn to exactly the same scale, the basic interactions of photons and matter are placed in relation to the properties of the light (bottom of image)

Figure 3 shows a simple conversion tool which relates the various units employed by different disciplines to describe the properties of light quantitatively.

### Heat and light

This review focuses on imaging and detection and with particular focus on light as carrier of signals. Prior to considering light–matter interactions it, however, it is necessary to describe the structure of solid matter, as governed by several quantum–mechanical rules, and the response of matter to heat, which conditions many of the behaviours of matter—often by quantitative exponentiation. Many of the properties of matter are strongly dependent on heat. An example is water, which in an extremely narrow range of temperatures is not solid or a gas. Heat is relevant in nanomedical applications and requires careful consideration, because in materials sciences, the common description of properties refers to their characteristics at either at 0 °C or at room temperature. The characteristic temperature of medically relevant biological systems is, however, significantly higher at 37 °C. The dependence on heat being usually exponential (see below), the 20 °C difference between body heat and room temperature may be dramatic (see below). Heat and its effects are properties of bulk materials and thermodynamics as applied to populations of particles (that is, to everything important in nanomedicine) joins hands with statistical mechanics to describe interactions. This applies at the deepest level of interactions in matter: it is necessary in the quantum–mechanical analysis of electron properties to descend to the temperature at absolute zero, in which the effects of heat can be ignored (see below). The description of matter in terms of statistics, that is: in terms of probabilities, is also fundamental. This is, however, not the usual application of statistics which focuses attention upon average values of populations. In studying the electronic properties of matter it is much rather the behaviour of extremely small numbers of particles with properties far from the average value that is important. Understanding the bandgap behaviour of electrons requires serious attention to vanishingly small populations of electrons which mingle with immensely large populations of electrons that show behaviour much closer to the average. These electrons, which are “black swans” are centrally important in diverse fields of electronics, opto-electronics and in a host of enterprises that use the products developed in these fields. In describing the behaviour of electrons in solid materials, the disciplines of quantum mechanics, thermodynamics and statistical mechanics meet and influence one another at the deepest levels. Before turning to the interactions of light with matter, this text reviews the fundamental principles of the structure of bulk matter, and the strong response that matter shows to alterations of temperature.

### Just heat

Heat shakes crystal lattices and increases the speed with which electrons move within crystals. The constant named by Max Planck (1901) after Ludwig Boltzmann, *k*_B_, features in essentially all mathematical descriptions of matter at temperatures above absolute zero (0 degrees Kelvin: 0 K). The product of *k*_B_ with temperature (in  K)—*k*_B_*T*—describes the average kinetic energy of electrons at the temperature *T*. At room temperature, about 300°K, *k*_B_*T* = 0.026 eV, which is the energy available to most electrons in the material. A tiny proportion of the electrons will be thermally agitated to oscillate around their lattice positions far more strongly than most electrons, they have much higher energies available, and this spread of energies requires statistical mathematics to describe it. The properties of materials at 7 °C = 280 K, 27 °C = 300 K, 37 °C = 310 K are quite different (see below). Intrinsic semiconductors are characterized by an exponential dependence of conductivity with temperature, as electrons are excited across an gap in energy (the bandgap), where there are no states. Electrons in the conduction band, and holes (the absence of electrons) in the valence band can then move under an applied field giving rise to useful conductivity. Most semiconductors in their pure form are not good conductors, they need to be doped to become conducting.

## Quantum aspects of matter

### The availability of electrons in materials

Free electrons can have any level of energy. In a crystal, the electrons interact with the periodic potential of the lattice formed by the atomic nuclei: they are restricted in the energy levels they may have. This review focuses primarily on the electrons that occupy the “conduction band” of atoms in crystals. In 2018, major research efforts focus on these electrons, but the industrial researchers are mainly interested in the electrical conductivities that are due to these electrons. For nanomedicine, the major interest lies in the availability of large numbers of electrons able to interact with various frequencies of electromagnetic radiation (see Fig. 3), absorbing some frequencies and emitting others in photoluminescence processes. The following much abbreviated account explains the supply of electrons to the conduction band, and shows that some aspects of electron behaviour are precisely calculable. There does remain considerable space, however, for application of the creative imagination.

Modern explanations of the behaviour of matter base on quantum theory, in which particles have wave nature. Within a single isolated atom, the electron has four quantum numbers and its possible energy states are defined as


**Fig. 4:**
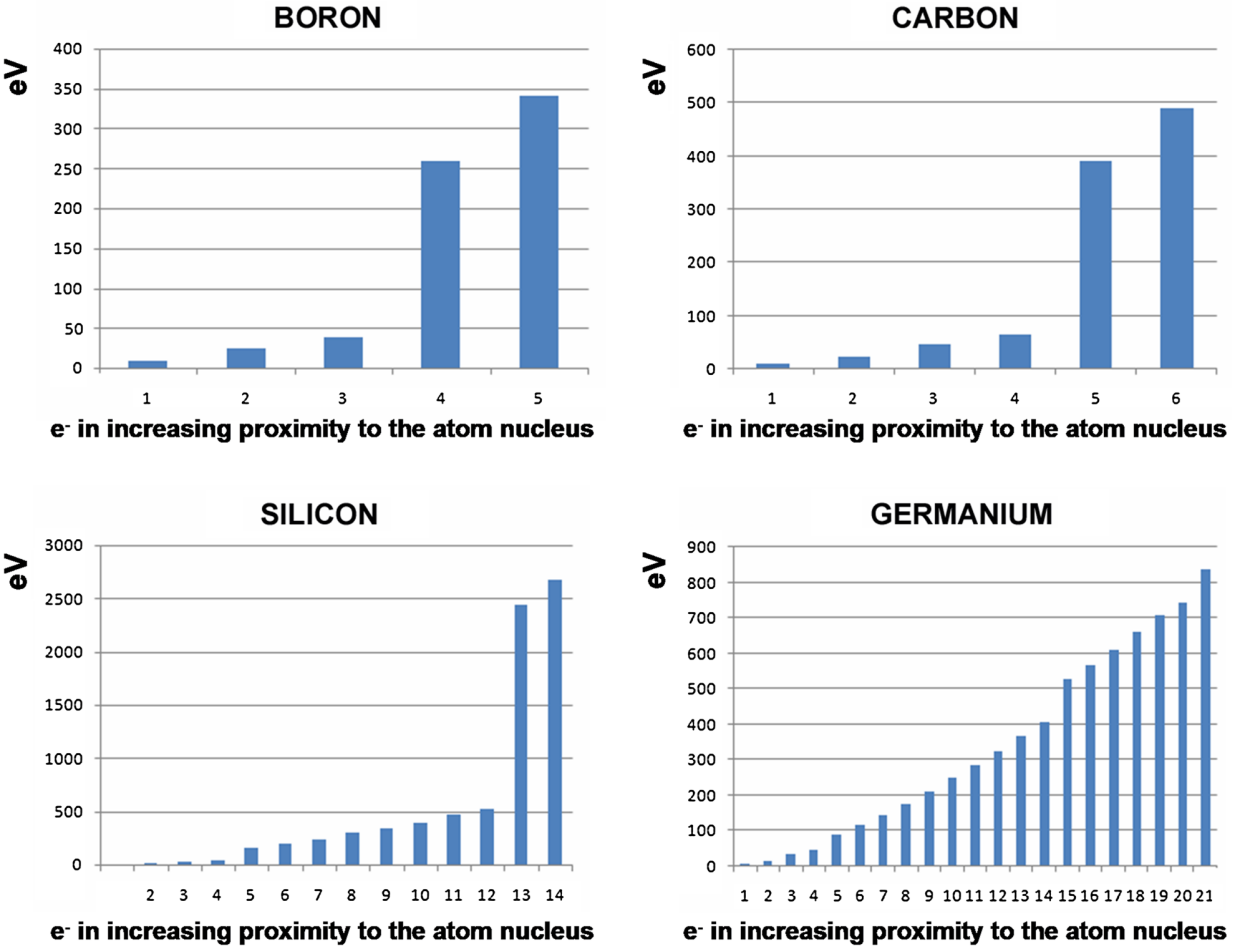
Energies required to strip successive electrons away from four elements. The outermost electrons are shown at the left of the bar diagrams. Note that these “ionization energies” reach levels of hundreds or thousands of eV for the innermost electrons nearest to the atomic nuclei, at right of the bar diagrams

*E*_*n*_ are the energy levels of the shells *n* = 1, 2, 3… or s, p, d, g …., and: *Z* = atomic number of the element. For silicon with *Z* = 14, *E*_1_ = − 2666 eV. Electrons in the inner layers, exposed without shielding to the total electrostatic pull of the nucleus, are bonded tightly to the atomic nucleus. Figure 4 shows that removing the innermost electrons from the atom is associated with an energy cost measured in thousands of electron volts. Electrons in the outer layers, shielded by the inner electrons from the pull of the atomic nucleus, are loosely bonded and can be removed by application of only a few electron volts (Fig. 4).

Although the energy costs of removing the outermost electrons are only a few electron volts, the average kinetic energy of electrons at room temperature is too small (0.026 eV) to tear them off the atom and hence to ionize it. The core electrons of the atoms are tightly bound and remain in their orbitals throughout all the phenomena described in this review. Only the outer electrons of atoms participate in the interactions involved in interatomic bonding, electrochemistry and photochemistry. The electrons in the outermost (or next innermost) shell are termed “valence electrons” and these energy states are called “valence shells”. Valence electrons are those electrons in an atom that can participate in formation of chemical bonds.

In single, isolated atoms, electrons having the same quantum number cannot occupy the same energy state (Pauli exclusion). Interaction of the electrons with the atom’s nucleus restricts the electrons to certain “allowed” orbitals (energy levels) and excludes them from certain “forbidden” energy levels. In pure crystals, consisting of lattices of atomic nuclei with their bound electrons, the electrons do not interact only with the nuclei to which they are most closely bound, they also interact with all other nuclei in the crystal. The orbitals of the outermost electrons grow into quasi-continua of energy states called “bands”. The band theory of metals was initially developed by Arnold Sommerfeld, from 1927 onwards (Sommerfeld 1964), who paid great attention to the underlying thermodynamics and statistical mechanics. The study of energy bands and bandgaps was built on theoretical foundations established in the 1930s and on the advances in preparing highly purified materials (especially germanium and silicon) during the 1940s; and bore fruits early in the 1950s (Shockley 1950, 1953, 1956; Brattain 1957) and is in the 21st century a well-established field with relevance to almost every aspect of modern life.

**Fig. 5:**
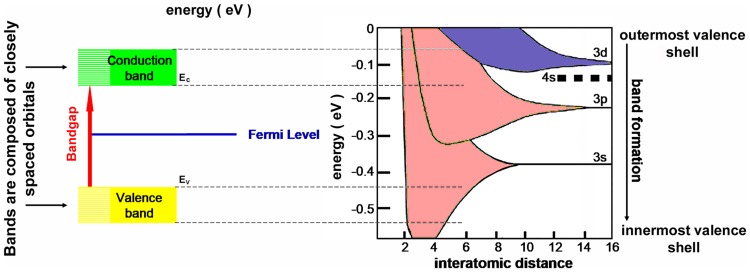
At large interatomic distances the individual atoms have energy states “orbitals” occupied by electrons (at the right of the image). As the interatomic distances shrink, because the atoms have close neighbors in crystal lattices the outer orbitals of the atoms (here, the s, p, and d orbitals) interact with the nuclei of all the atoms in the material sample, and due to Pauli exclusion they split into the same number of orbitals as there are atoms present in the sample. A typical crystal may contain 1 × 10^22^ atoms and about 1 × 10^20^ energy states are formed, each energy state being limited to a size of ~ 1 × 10^−22^ eV. The contraction of the lattice sizes (moving to the left in the diagram) confines the bandgaps, increasing their energy and raising the amount of energy an electron needs to jump from the valence band (*E*_V_) to the conduction band (E_C_)

**Fig. 6:**
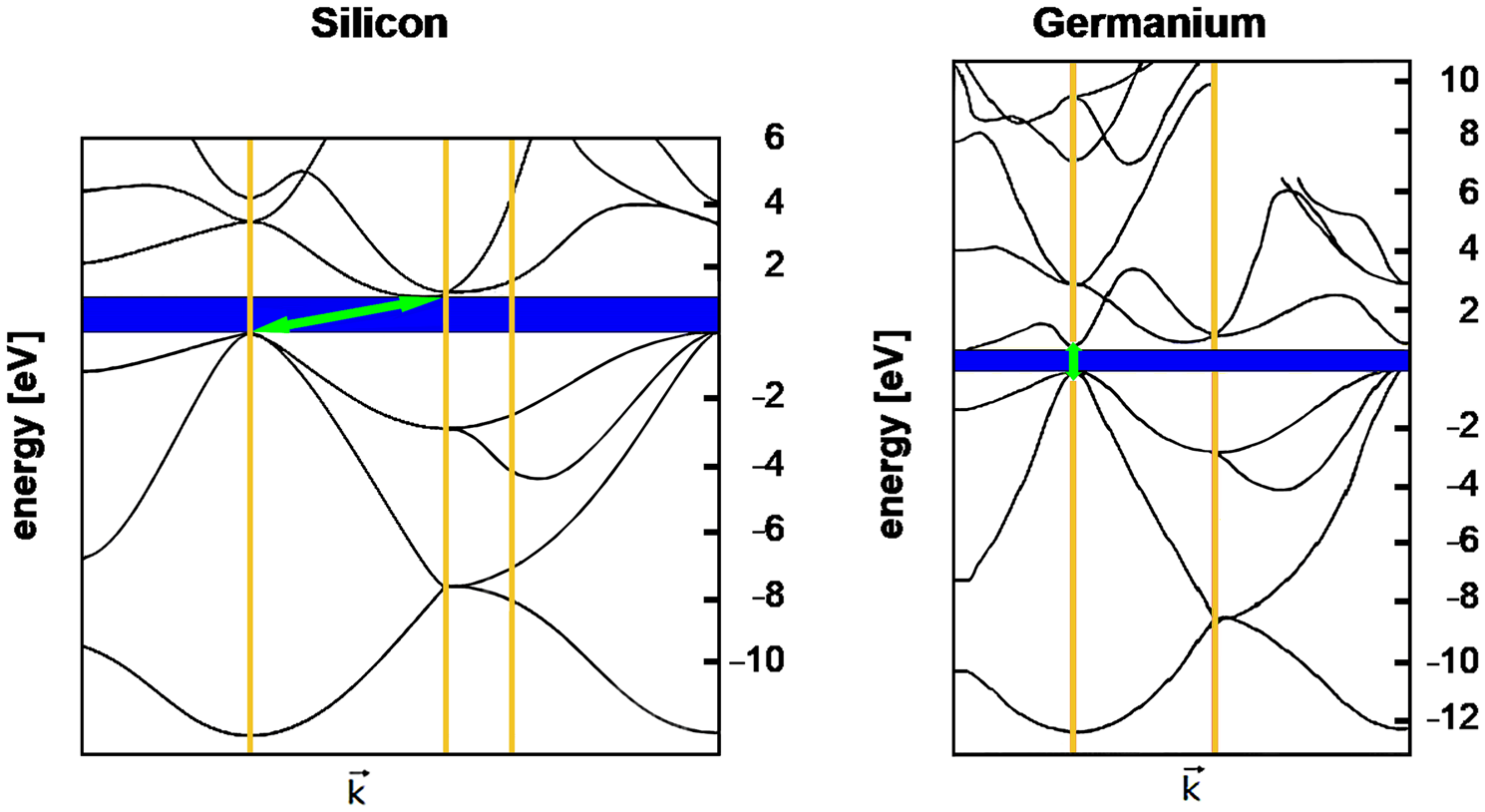
At left: the energy band structure of silicon. At right: the energy band structure of germanium band structure. The complexity of the band structures is evident. The bandgap containing the Fermi level is shown here in blue (the Fermi level itself is not shown). The bands are mapped in “k-space”, which visualizes the interactions of the electrons with the unit cell of the crystal lattice. The energy level in such diagrams is usually based on the Fermi level being denoted as zero, as shown here. The energies involved are ~ 6 to 10 eV. In silicon the highest level of the valence band is offset against the lowest level of the conduction band above it (green arrow): silicon, therefore, has an “indirect” bandgap. In contrast, germanium has a “direct” bandgap (green arrow)

**Fig. 7:**
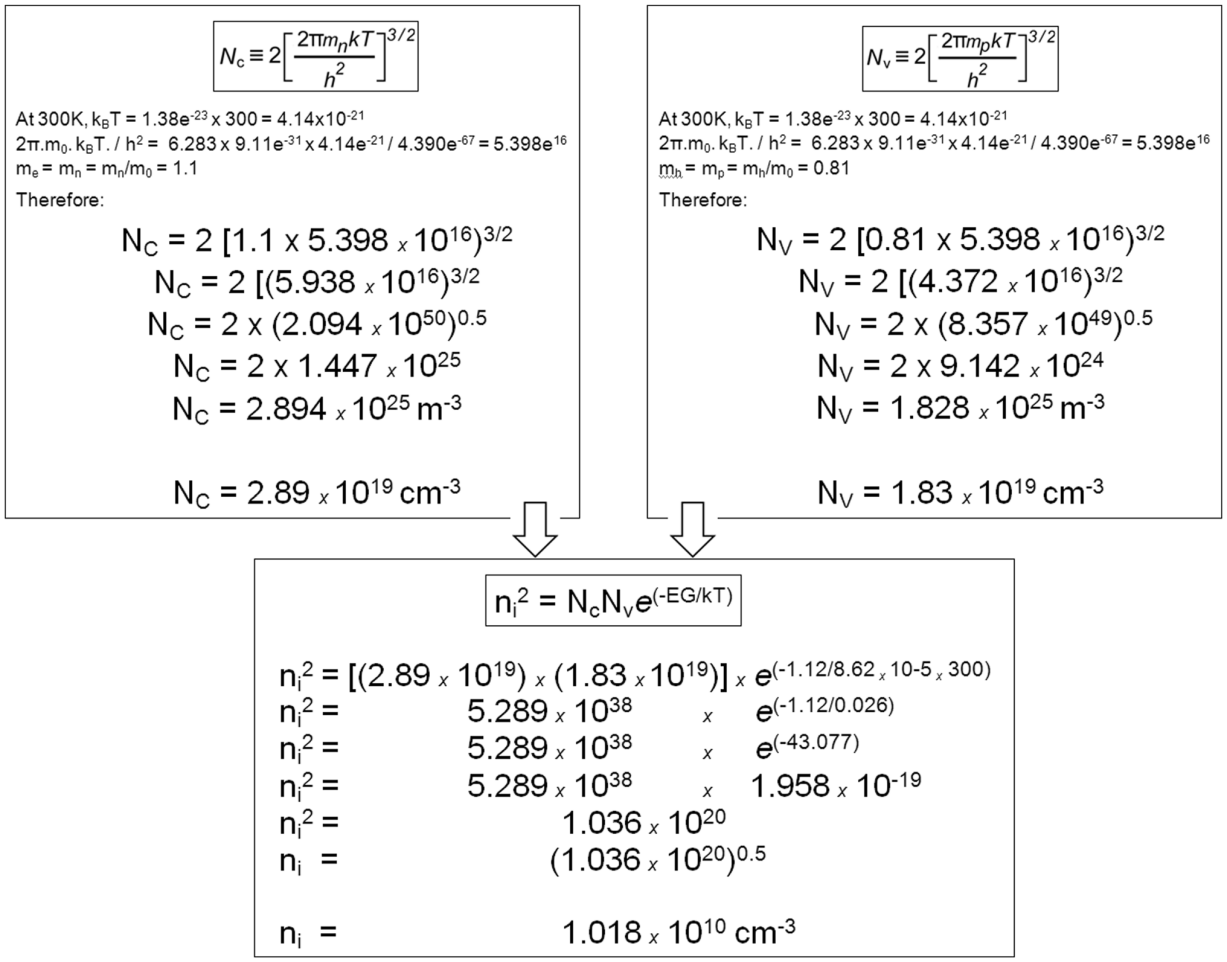
After the effective masses of the charge carriers in a semiconductor have been measured (*m*_e_ = *m*_n_ for the electrons, *m*_h_ = *m*_p_ for the holes), the two values can be entered into the formulae at the top of the figure to calculate the density of energy states available: *N*_c_ for the negative charge carriers (electrons) and *N*_V_ for the positive charge carriers (holes). The calculated values for *N*_c_ and *N*_V_ can then be used in a second step to calculate the number of electrons in the conduction band (or holes in the valence band). The calculation shown here begins with the effective masses of the electrons and holes measured for pure silicon crystal: *m*_e_ = 1.1 and *m*_h_ = 0.81. The first step of calculation gives the density of energy states available: *N*_c_ = 2.89 × 10^19^ per cm^3^ for the electrons and *N*_V_ = 1.83 × 10^19^ per cm^3^ for the holes. The second step of calculation gives the number of charge carriers as *n*_i_ = 1.018 × 10^10^ per cm^3^, meaning that the conduction band is populated by this density of electrons. This very low density means that pure silicon is an insulator (see below)

In bulk materials, atoms are packed so closely together that the outer electrons’ wave functions overlap and the outer electrons of each atom interact with the nuclei of all the atoms in the material. As free atoms are brought together, the Coulomb interaction between the atom cores and the electron splits the energy levels, spreading them into bands. Each state of a given quantum number of the free atom is spread in the crystal into a band of energies. Since the number of atoms in 1 cm^−3^ of silicon at room temperature is ~ 5 × 10^22^, the result of these numerous interactions is that a bulk property of matter emerges: the orbitals of the outer electrons merge into bands of energy levels, the individual energy levels separated by ~ 1 × 10^−22^ eV: in effect, the band represents a continuum of allowed energies. The “forbidden” energy levels now appear as merged “forbidden” levels and are termed “bandgaps”. Bands and bandgaps can be identified and analyzed by spectroscopical methods (Helmholz and Voon 2002). The number of bands depends on both temperature and on the lattice size. Electrons in the ground state (at absolute zero, see below) occupy the lowest available energy states first, filling most energy bands which once full are inert. Such bands cannot contribute to an electric or thermal current. Only partially filled bands need to be considered in calculating the electronic properties of a solid. In summary, the inner electrons of the atoms remain tightly bound to the nucleus and do not form energy bands, whereas the outermost (valence) electrons occupy complex systems of multiple energy bands amongst which there are bandgaps (Fig. 5). The complexity of the bandgaps is sometimes overlooked, as a glance at Figs. 5, 6 and 7 shows. Usually there are multiple bands and multiple bandgaps between them. Only one, however, is of interest to nanoparticle technology, namely, the bandgap associated with the “Fermi level”. To identify this bandgap, which plays the major role in the crystal’s electronic properties, the bands are calculated in their “ground state” at 0  K, absolute zero: the temperature at which the quantum properties of the band structures are not disturbed by heat. This identifies the band which is filled with (outermost) electrons and which is termed the “valence” band. Above the valence is the bandgap and above that is the band termed the “conductance” band, which contains no electrons. At 0 K the crystal has no electrons available that could move through the lattice as electric current: it is an “insulator”. The Kelvin scale, with 0 K as the lowest point on the scale, is used for all discussion of energy bands in materials. It is useful to remember that 1 °C = 1 K, but starts counting at 273.15 K. Thus 20 °C = 293.15 K; 37.5 °C = 310.65 K and 60 °C = 333.15 K. The bandgap is a characteristic property of any given material and it is a property of the bulk material, not of the single atoms. The application of heat raises the temperature of the crystal above 0 K, so that thermal effects are added to the purely quantum-defined energy bands. This introduces probability effects into materials science.

Thermal influences introduce uncertainty about the individual electrons. It is not possible to assign energy values to the individual electrons, so the bulk properties of the huge populations of electrons (~ 5 × 10^22^ cm^−3^) dominate the picture. The statistical–mechanical model known as the “Dirac-Fermi” analysis computes the probabilities of electrons being present in any given energy state and defines the “Fermi” energy level *E*_F_ as that energy level for which the probability of occupation by an electron is 50%. There is only one Fermi level in a system at thermal equilibrium. It is defined as the energy level midway between the top of the valence band and the lowest energy level (bottom) of the overlying conduction band. In a pure (“intrinsic”) semiconductor, the Fermi level is at the exact center of the bandgap; for half the time it is not occupied by any electrons. The probability an energy state is occupied approaches 1.0 if the energy of electrons in it is much lower than *E*_F_, and approaches zero if their energy is much higher than *E*_F_. Note that in this text we ignore the difference between the Fermi energy and the Fermi level, which at 0 K are identical but at higher temperatures diverge slightly, by a few milli-electron Volts (meV).

The Fermi level is of crucial importance for all the remainder of our analysis. As temperature increases from absolute zero some electrons are promoted by thermal collisions into the band above the Fermi level. The valence band loses electrons and is no longer totally full, the conduction band gins some electrons and is no longer fully empty. The conduction band gains electrons, the valence band gains the vacant sites from which the electrons were boosted out of their covalent bonds, the vacancies are known as “holes” and behave like quasi-particles. Thus the Fermi level separates the energy levels (in the valence) band at which no electrons are available for chemical or physical interactions, from the energy levels (in the conduction band) which are available for chemical and physical interactions and which form an electron gas that can be extremely rarified or extremely dense. Much of the remainder of this quantum–mechanical and statistical–mechanical discussion centers upon the ways in which the bandgap can be occupied, how the position of the Fermi level can be shifted within the bandgap, and of the density of the electrons in the energy bands above the Fermi level. The characteristics of the conduction band can be calculated using one of the models based on the Schroedinger equation. The calculation requires the insertion of a parameter that strongly reflects the quantum nature of crystals, namely, the “effective masses” of electrons and holes in crystals. The strangeness of the concept “effective mass” is of considerable intrinsic interest, but also of great industrial and scientific importance. We consider it briefly here, basing the text loosely on the Lecture on this topic given by Feynman et al. (1964).

Following the pioneering work by David Pines and David Bohm (Bohm and Pines 1951, 1953; Coleman and Greene 2018; Pines and Bohm 1952) in the 1940s, electrons are considered to form an electron gas in the conductance band, above the Fermi level, a gas to which statistical principles apply and in which individual electrons are free to move through the crystal. The lattice of positive nuclei lies all around them and they pass through it, or “drift” through it at speeds that depend on the lattice characteristics, the temperature and the strength of any applied electric field.

In crystalline silicon the face-centered unit cell has lattice constant = 5.431Å = 5.431 × 10^−10^ m, and flowing electrons will pass at least two of the silicon atoms in the cell: therefore, under application of a 50 V electric field, the electrons traverse [7/5.431 × 10^−10^] lattice constants = 1.29 × 10^10^ lattice constants per second, and, therefore, flow at a rate of approximately 20 milliards of “amplitude pips” per second, as Feynmann would state it (Feynman et al. 1964). At higher voltages the electrons achieve much higher speeds (in some cases such as graphene, relativistic speeds). The speed of electrons as they pass through the Ångstrom-sized gaps in the densely packed crystal lattices is comparable to the speed of electrons streaming through the vacuum! The electrons apparently pass through the densely packed crystal solid as if it were not there. It is this that allows transistors to replace thermionic vacuum tubes, even for applications that require extremely high switching rates. The explanation is purely quantum mechanical: provided the crystal structure is everywhere uniform—perfect and containing no defects—the wave properties of the electron allow the formation of wave packets with group velocities that in classical mechanics would represent speeds. The wave packets are patterns of displacements which propagate through the crystal as a wave of a single, fixed frequency. The electron amplitudes oscillate as the wave packet passes from one atom to the next; the quantum–mechanical oscillation has the same size at every atom, but the phase alters in steps that equal the distances between neighboring atoms. Out of such quantum–mechanical calculations there emerge two constants, *m*_e_ and *m*_h_, which quantify the effective masses of electrons and holes, respectively, as they pass through this particular crystal. They are termed “effective masses”, but are better compared with momentum than with mass; their “masses” are independent of, but similar in general magnitude, to the mass of the free electron (Feynman et al. 1964). The electron’s effective mass is often stated in ratio to the electron’s rest mass (see Table 1 and Fig. 7). The macroscopic properties of the material, therefore, depend directly upon the quantum–mechanical wave properties of the particle termed “electron”. In anisotropic crystals the effective mass of a single electron takes different values depending on the direction the electron is taking through the crystal: they are different along the different axes of the crystal, and can be calculated from knowledge of the axes. The effective masses of both electrons and holes in a crystal material can be measured, for example, by cyclotron resonance. From their values the concentrations of the charge carriers (electrons, holes) in their respective bands can be calculated for any given temperature, as shown for 300 K in Fig. 7. From these concentrations (“*n*_i_” in Fig. 7) numerous other properties of the crystal can be calculated, and each of these properties can be compared with values measured in the laboratory. The mathematics underlying the derivation of the formulae used for this are university level, but the formulae themselves allow simple calculation by use of any scientific pocket calculator. Figure 7 shows a finger exercise demonstrating this simplicity. Table 1 provides data from which the reader can herself calculate from the effective masses in germanium and gallium arsenide the supply of electrons available in the conduction bands of pure examples of these semiconductors.

**Table 1:** Bandgap characteristics of some insulators, semiconductors and metals

Material	Bandgap (eV)	*m*_e_ (*x* m_0_)	*m*_h_ (*x* m_0_)	*N*_V_ at 300°K (cm^−3^)	*N*_c_ at 300°K (cm^−3^)	No. of atoms/molecules in 1.0 cm^3^	*n*_i_ (300°K, cm^−3^)	Fraction of atoms providing conduction electrons	Conductivity [*σ* (S/m), 20 °C]	Conductivity compared to pure silicon
Insulators										
Diamond (undoped)	5.47	–	–	–	–	1.754e^23^	0	0	~ 1e^−16^ to ~ 1e^−18^	2.30e^−13^ to 2.30e^−15^
Silicon dioxide	8.9	–	–	–	–	2.204e^22^	0	0	~ 1e^−16^ to ~ 1e^−18^	2.30e^−13^ to 2.30e^−15^
Semiconductors										
Germanium (undoped)	0.67	0.55	0.34	4.97e^18^	1.02e^19^	4.41e^22^	1.81e^13^	4.1e^−10^	2 × 10^0^	4.60e^3^
Silicon (undoped)	1.12	1.1	0.81	1.83e^19^	2.89e^19^	5.00e^22^	1.02e^10^	2.0e^−13^	4.35e^−4^	1.0
Gallium arsenide (undoped)	1.42	0.067	0.47	8.08e^18^	4.35e^17^	2.21e^22^	2.59e^6^	1.2e^−16^	1e^−6^	2.30e^−3^
Conductors										
Gold	–			–	–	5.8978e^22^	5.8978e^22^	1	4.10e^7^	9.43 × 10^10^
Silver	–			–	–	5.8575e^22^	5.8575e^22^	1	6.30e^7^	1.45 × 10^11^
Copper	–			–	–	8.4661e^22^	8.4661e^22^	1	5.96e^7^	1.37 × 10^11^

Table 1 compares silicon with two other semiconductors, with three metals, and with two insulators. In the case of metals, which for each atom contribute one electron to the pool of conduction electrons (there is a Fermi level in metals but it is not located in a bandgap—the majority of the conduction electrons in a metal occupy a band of completely filled states with energies far below the Fermi energy. In many cases, such electrons have very little effect on the macroscopic properties of the metal.

We consider more closely the question of electron motion through a densely packed crystal lattice. It yields important parameters used to describe energy bands and bandgaps. The mobility of electrons (*μ*_e_) in a crystal is a characteristic property of the crystal and of the temperature, and the resulting electron drift (*V*_d_ = *μ*_e_*E*) in an electric field is expressed in the terms “m/s *×* V/m” and, therefore, as “m^2^/V *×* s”. Table 2 shows the simple calculation involved while also underlining some principle features of electron mobility, for example: that the average drift velocity of electrons in the absence of an electric field is zero.

**Table 2:** Finger exercise for calculation of electron speeds within materials from data readily available in the public sphere

Material	Electron mobility, *μ*_e_ (m^2^/V s)	Applied electric field, *E* (V)	Electron drift velocity, *V*_d_ (m/s)
Silicon	0.14	0	(0.14 × 0) = 0
Silicon	0.14	50	(0.14 × 50) = 7
Germanium	0.38	1000	(0.38 × 1000) = 380

Measurement of the movement speeds of the charge carriers then allow the conductivity of the material to be calculated. For completion, we note that the “hole” in the valence band also has an effective mass (different to that of the electron) and that it also can migrate within the crystal lattice, though at slower speeds than the electrons, because each movement that a hole makes requires the breaking of a bond between two atoms. In silicon, which has *μ*_e_ ≈ 1400 cm^2^/V s, the mobility of the hole is *μ*_h_ ≈ 500 cm^2^/V s.

In analyzing a semiconductor an early step is obtain X-ray diffraction data allowing determination of the crystal structure. From this knowledge of the structure the parameters of the phonons in the crystal can be calculated and thus the thermodynamic properties of the crystal obtained. From the crystal structure the electronic band structure can be calculated. Only the top of the valence band and bottom of the conduction band are important for most electronic properties.

## The semiconductor bandgap

**Fig. 8:**
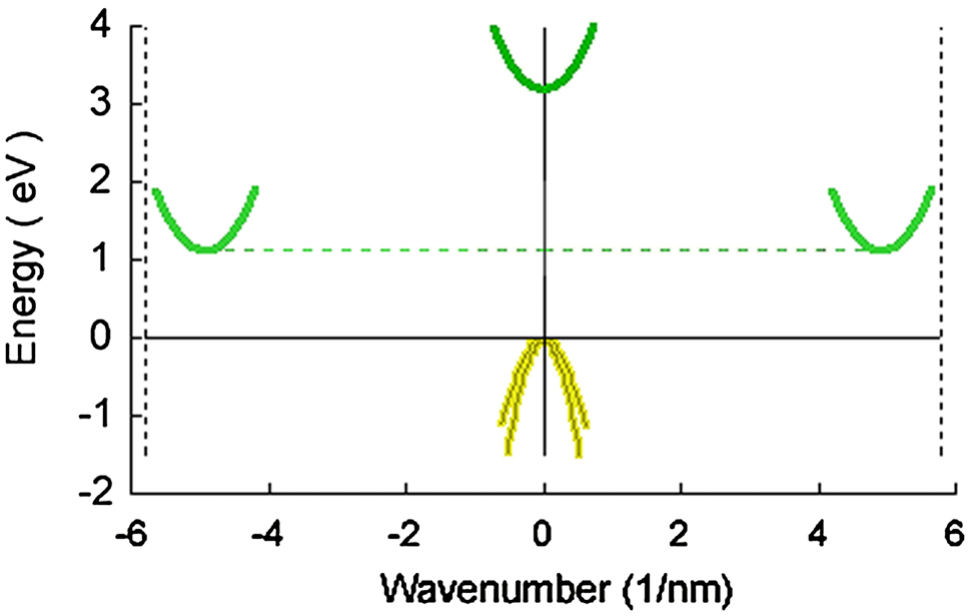
*E*–*k* diagram of silicon, including the primary band minima and maxima. The complex configuration shown here in two dimensions represents a section through the higher dimensional complexity of the total bandgap; for comparison, check Fig. 6. The yellow peak(s) represent the highest energy levels of the valence band; the green troughs represent the lowest energy levels of the conduction band. The dashed green line connecting the two troughs of the conduction band are 1.12 eV above the peak of the valence band: note the offset between those troughs and that peak, meaning that silicon has an “indirect” bandgap (compare Fig. 6)

The bandgap is usually depicted as a juxtaposition of two plane surfaces, represented in most diagrams by two parallel lines. In fact, bandgaps have complex 3-dimensional surfaces with one or more parabolic regions which play significant roles in bandgap characteristics. The band structures depicted above (Fig. 6) suggest the finer structures that will become evident on closer examination. Figure 8 offers a two-dimensional view of the complex series of “valleys and hills” that form the topography of the silicon bandgap, and which have major influences on, for example, the effective masses of the electrons and holes.

**Fig. 9:**
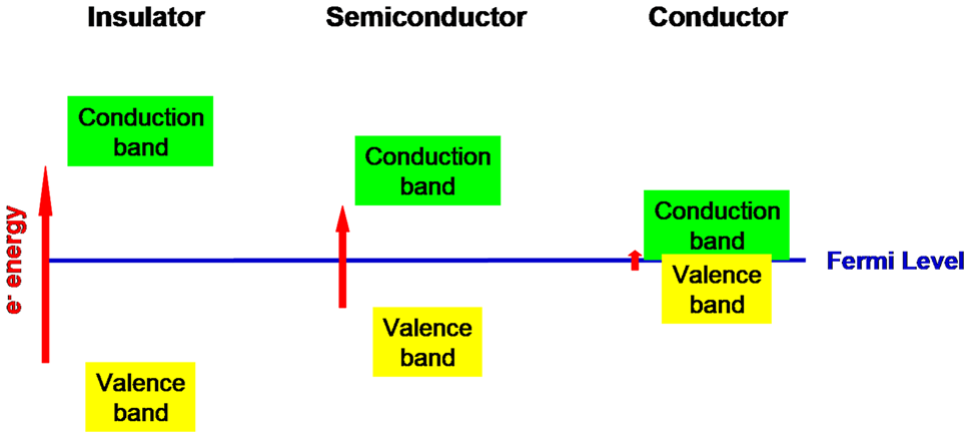
Bandgaps of three types of matter. In insulator materials the bandgap is wide—above 3 eV—and at room temperature, the energies of photons are insufficient to propel electrons across the bandgap at collision. In semiconductor materials the bandgap is narrow enough—from 0 eV to ~ 2 eV—that at room temperature photons in the visible part of the spectrum and adjacent regions of the spectrum can eject electrons out of the valence band into the conduction band; the Fermi level is exactly halfway between the energy level of the conduction band and that of the valence band. In conductors, such as metals, the valence and conduction bands overlap and electrons easily transfer into the conduction band: the Fermi level is not a critical feature in conductors

**Fig. 10:**
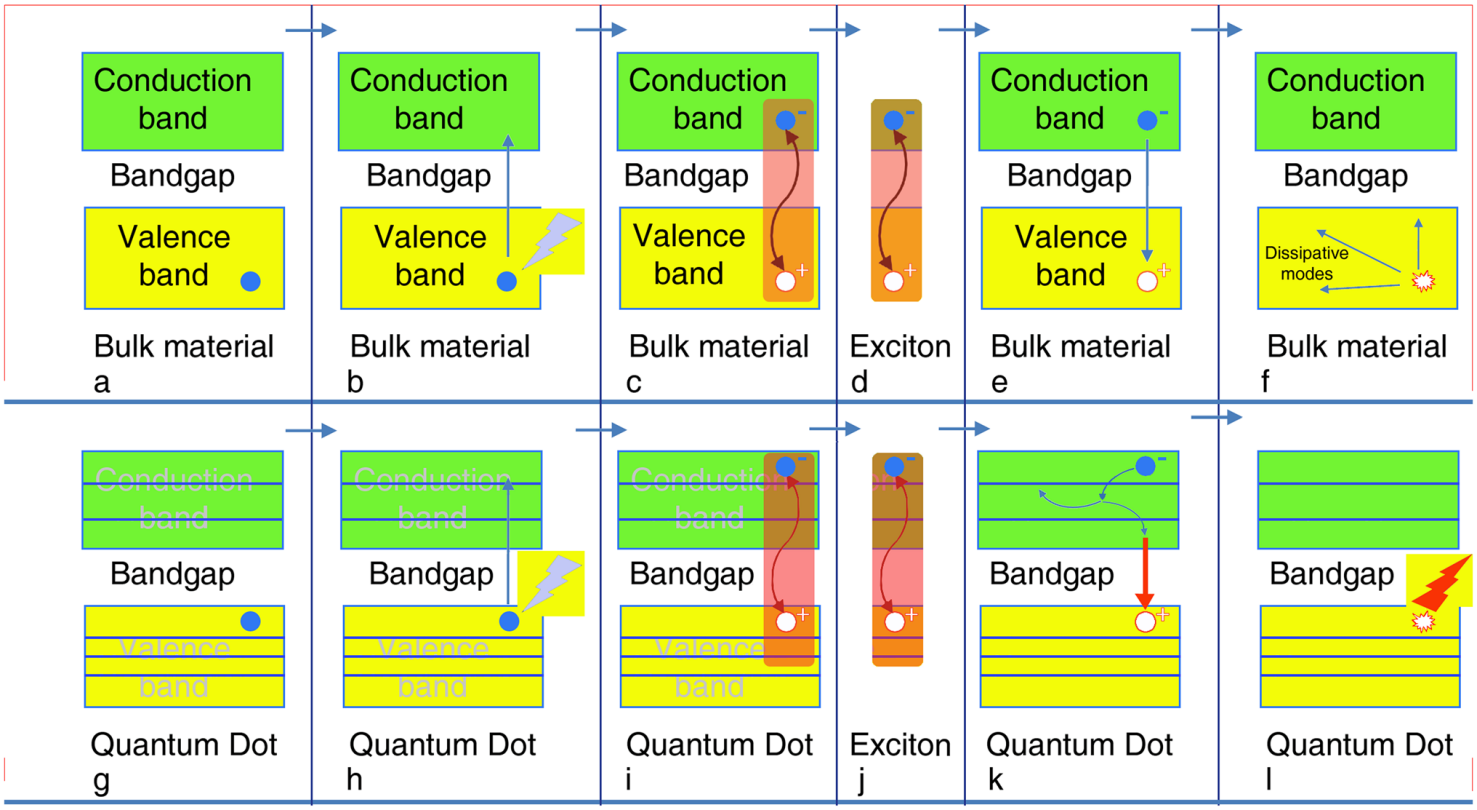
Simplified Jablonski-type sketch diagram of the differences between bulk semiconductor material and quantum dots (QDs). Interaction with a photon does not result in photoluminescence in bulk material (**a**–**f**), whereas it can do so with high efficiency in QDs (**g**–**l**). Note the dissipation of excess energy coupled with bandgap-determined exciton decay in **k** (bold red arrow), causing bandgap-energy-related photon emission in **l**. In the case of radiative recombination, this energy is emitted in the form of a photon. In the case of non-radiative recombination, it is passed on to one or more phonons and in the case of Auger recombination it is given off in the form of kinetic energy to another electron

**Fig. 11:**
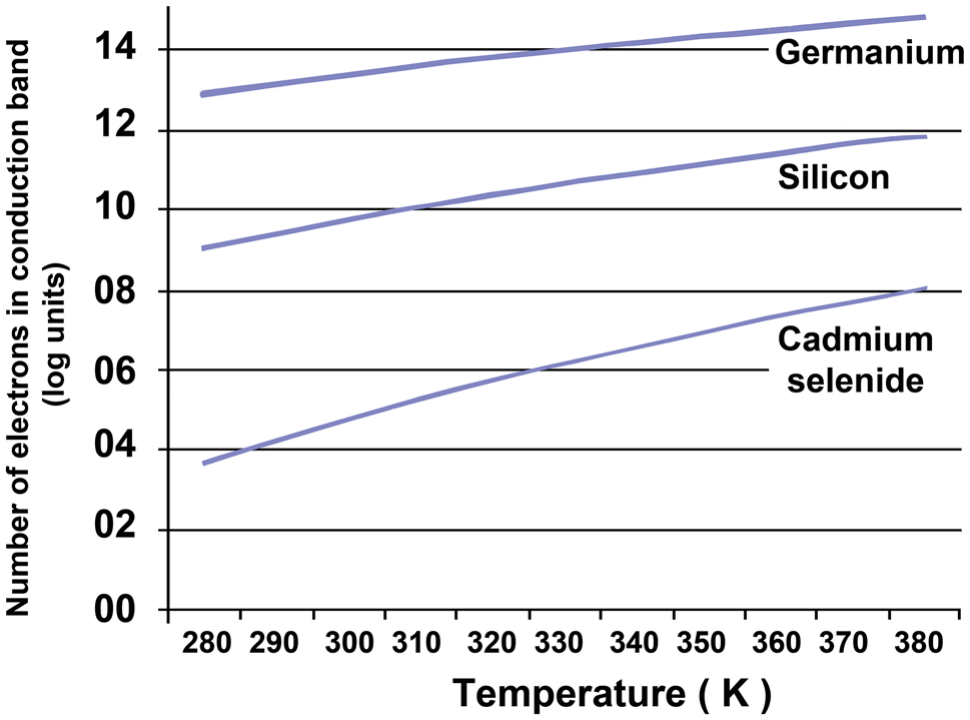
Number of electrons in the conduction band of semiconductors having different bandgap sizes. The dependence of electron supply on temperature is shown, and it follows similar courses in materials having bandgaps up to 1.5 eV in size, as shown here. Note that, as in Fig. 12, the size of the bandgap varies in a linear fashion (*x*-axis) but the consequent supply of electrons to the conduction band varies exponentially (the *y*-axis rises from 1 × 10^5^ to 1 × 10^12^ on the *y*-axis)

Figure 9 shows the essential difference, the bandgap width, which distinguishes between the three types of matter, the insulators, the semiconductors and the conductors. Figure 10 shows how the bandgap is essential for exciton-mediated photoluminescence. Figure 11 shows the temperature dependence of the electron supply to the conduction band.

The electrons available for conduction, absorption, emission and other purposes are those in the conduction band above the bandgap containing the Fermi level. (We will later discuss the case of metals, which have no bandgap, below). The central task of basic semiconductor physics is to establish formulae for the position of the Fermi level *E*_F_ relative to the energy levels *E*_C_ and *E*_V_ (the level of the bottom of the conduction band and the top of the valence band), taking into account the effects of “doping”.

Electron mobility in a crystal is measured using the Hall effect. Conductivity can be measured. The bandgap of a semiconductor can be measured (it is located at the energy (= wavelength) at which the material ceases to be transparent).

## Effects of bandgap size

**Fig. 12:**
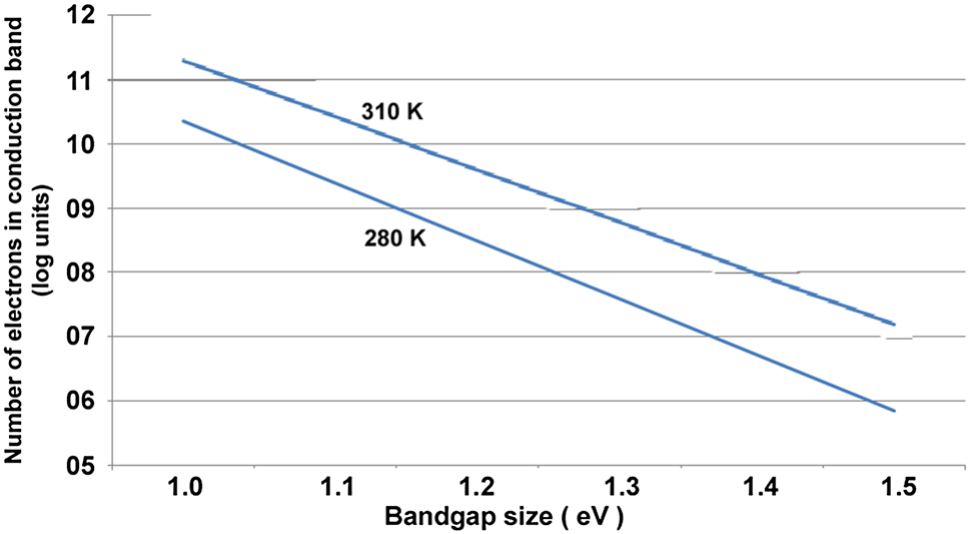
Number of electrons in the material’s conduction band at different temperatures: 280°K and 300°K. Although the three materials have significantly different sizes of bandgap (see Table 2), they all respond in a similar way to an increase in temperature. Note the x-axis here is linear, but the supply of electrons to the conduction band (y-axis) responds exponentially to the change in temperature. A 10 K rise in temperature causes an order of magnitude increase in the supply of conduction band electrons

At a bandgap of 2.3 the number of electrons reaching the conduction band is only 1 electron per cubic centimeter (Fig. 12).

**Fig. 13:**
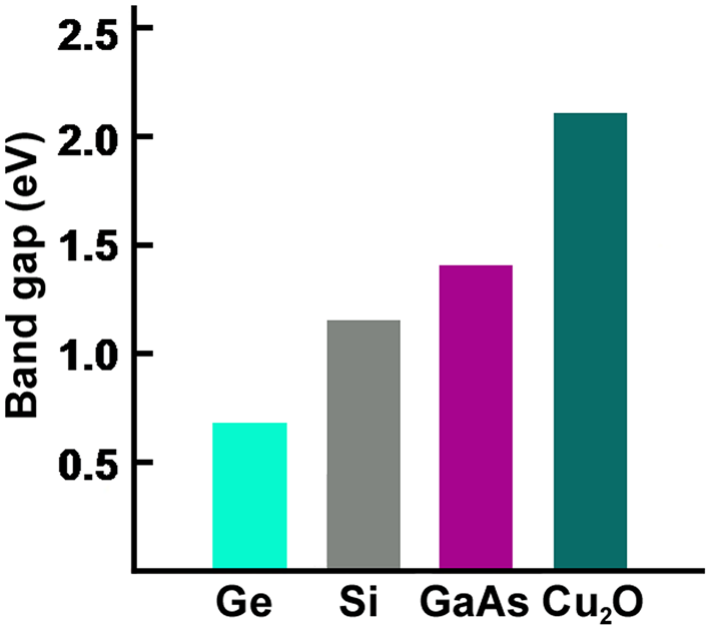
Bandgaps of four intrinsic (undoped) semiconductors. The bandgaps of materials can be as low as zero (in metals) or as high as 8 in insulators such as some types of glass. In semiconductors, bandgaps range from ~ 0.2 to ~ 2.5 eV

We summarize the discussion about bandgaps: Electrons that can be used to generate signals must be free for interactions. The atom’s core electrons are too tightly bound to the nucleus to be used. The central issue is the presence of a bandgap (in insulators and semiconductors but not in metals). The bandgap is a characteristic property of a material and is a property of the bulk material (Fig. 13), not of the single atoms. The relative sizes of the bandgap (in eV) and the forces that can give sufficient impulse to impel an electron across it are of considerable importance in all areas involving photoluminescence. Photons having energies greater than the bandgap are of great importance. Thermal fluctuations at a range of temperatures are of great importance, and are quantified by the kT number.

*The bandgap depends on several of the properties of the material,* namely its atomic lattice spacing, the presence of more than one type of material, the purity of the material and the presence of dopants, the pressure acting on the material, the temperature. Insulators have bandgaps so large that they prohibit entry of electrons into a state in which they can interact. Semiconductors have bandgaps of 1–3 eV which allow small numbers of electrons to become available for interactions. Since the band/bandgap structure of semiconductors is extremely complex, the bandgap that can be used for technological purposes must be identified.

The electronic structure of the pure crystal is considered in the absence of thermal influences, that is at zero Kelvins temperature, to define the Fermi energy which at higher temperatures is equal to the top level of the “valence” band in the material. Since thermal influences raise electrons in energy, above zero Kelvins some electrons are promoted to the “conduction” band of the material. As temperature increases higher numbers of electrons jump across the bandgap from the valence to the conduction energy band, and they do this is the random way that is described by statistical mechanics. Taking the electronic structures into account, also thermal influences and the statistical nature of electron promotion, the Dirac-Fermi model defines the “Fermi level” as the energy level in which the probability of finding an electron at that energy is 50%. The energy band just below the bandgap containing the Fermi level is known as the “valence” band and is usually nearly filled with electrons participating in bonding interactions forming the lattice of the material. The energy band just above the Fermi level is known as the “conduction” band and contains a much smaller population of electrons that can migrate and carry charge; these “negative charge carrier” electrons flow as electric current in the material. The absence of those electrons from the valence band is described as “hole” and the holes are termed “positive charge carriers”, though their migration mechanisms are more complicated than those of the electrons and noticeably slower.

This discussion will focus for the next few paragraphs on the semiconductor silicon, which is abundant, is in common use in many technologies, and which has been studied in great detail. Modern electronic devices are constructed with resistors, diodes, transistors, integrated circuits which are made by semiconductor materials. Nowadays, silicon is the most used semiconductor in power electronic components such as diodes, thyristors, MOSFET transistors. The reason is that the silicon is resistant to very high temperature and current. The maximum operation temperature of silicon transistors is 150 °C, while, for example, germanium transistor operates up to 70 °C. Silicon is not a conductor in the true sense of the word. It conducts electricity under certain conditions. It is a semiconductor material which is insulator at the absolute zero temperature (at 0 K). With increasing of temperature, a thermal energy will generate from covalent electrons a fraction which becomes free. Pure silicon has covalent bonds with energies of 1.1 eV, which quantifies how much energy it takes to free the valence electrons in the crystal structure.

Pure silicon has a bandgap which at room temperature is 1.12 eV wide. At room temperature the average kinetic energy available to electrons is about 0.026 eV (or 1 × *kT*) in size, so that most of the electrons have far too little energy to cross from the valence into the conduction band. Above 0 K extremely small numbers of electrons cross the bandgap, but once the temperature reaches a few *kT* below the Fermi level some electrons have enough energy to cross into the conduction band. The rate of crossing approximately doubles with every 10 K increase. Calculation displays the very small fraction of electrons that have sufficient energy to cross into the conduction band: 1.01 × 10^10^ electrons per cm^3^ (Table 1). Since the silicon valence band at 300°K contains 1.83 × 10^19^ energy states, only 1 electron in 1.83 × 10^19^/1.01 × 10^10^ = 1.8 × 10^9^ electrons cross into the conduction band. On the other side of the bandgap, pure silicon has 3.22 × 10^19^ energy states/cm^3^ in its conduction band, so the occupancy of the conduction band of silicon at room temperature is only 1 electron per [3.22 × 10^19^/1.01 × 10^10^ =] 3.19 × 10^9^ energy states. Raising the temperature 10°K approximately doubles the occupancy of the conduction band, and this response to raised temperature, which is characteristic of semiconductors, was already noticed by Faraday in 1832 (Faraday 1914). Pure silicon is, therefore, a weak conductor able to carry a current of only 1.56 × 10^−3^ *σ* (S/m) at 20 °C. Table 1 shows a comparison of silicon with other semiconductors, with three metals, and with two insulators. In the case of metals, which for each atom contribute one electron to the pool of conduction electrons, there is a Fermi level in metals but it is not located in a bandgap. A comparison shows the following figures:

$$1\;{\text{cm}}^{3} \;{\text{of}}\;{\text{copper}}\;{\text{weighs}}\;8.94\;g = 0.1406\;{\text{Mol}} = 8.4661 \times 10^{22} \;{\text{atoms;}}$$

$$1\;{\text{cm}}^{3} \;{\text{of}}\;{\text{silver}}\;{\text{weighs}}\;10.5\;{\text{g}} = 0.0973\;{\text{Mol}} = 5.8575 \, \times 10^{22} \;{\text{atoms;}}$$

$$1\;{\text{cm}}^{3} \;{\text{of}}\;{\text{gold}}\;{\text{weighs}}\;19.32\;{\text{g}} = 0.09797{\text{ Mol}} = 5.8978 \times 10^{22} \;{\text{atoms}} .$$

Gold, with 5.9 × 10^22^ atoms/cm^3^, has, therefore, 5.9 × 10^22^ electrons/cm^3^ available to carry current and a correspondingly high conductivity of 4.10 × 10^7^ *σ* (S/m) at 20 °C. Silver, with 5.9 × 10^22^ conducting electrons/cm^3^, carries 6.30 × 10^7^ *σ* (S/m) at 20 °C, and copper with density 8.5 × 10^22^ conduction electrons/cm^3^ can carry 5.96 × 10^7^ *σ* (S/m) at 20 °C. The metals conduct electricity far better than silicon, by the factors 2.56 × 10^10^ (gold), 4.04 × 10^10^ (silver) and 3.82 × 10^10^ (copper). These comparisons show that silicon is a very poor conductor of electricity and that this is explained with high quantitative accuracy in terms of its electron band structure and bandgap size. Note that the above text assumes only thermal fluctuations function as energy sources to promote electrons into the conduction band. Photons can also do this, and since photons with energies lower than the bandgap cannot be absorbed, it requires a photon of energy 1.12 eV to promote an electron to the conduction band in silicon at room temperature, and this is in the infrared part of the spectrum (Fig. 3). Photons as energy source will be discussed in more detailed below during discussion of plasmons.

## Doping of semiconductors

Modification of bandgap properties is at the focus of numerous industrial and academic research projects in 2018. Major efforts aim to improve photovoltaic efficiencies or to improve the function of electronic component such as transistors, displays, etc. As noted above, bandgaps can be modified by altering the ambient pressure or temperature, but this is rarely commercially useful. Instead, one resorts to doping. The capacity to respond to doping is part of the definition of semiconductors: “An electronic semiconductor is a valence crystal whose conductivity depends markedly on temperature and on the presence of minute amounts of foreign impurities”. The ability to change the electrical characteristics of the material through selective introduction of impurities is the basic reason why semiconductor devices are possible.

Doping inserts foreign materials into the crystal structure of the semiconductor. Consider the crystalline forms of Group 4 elements such as carbon, silicon or germanium. These have valence bands filled with electrons that are bound in covalent bonds: Crystalline diamond has a large bandgap (see Table 1), too large for thermally excited electrons to cross. We consider diamond more closely. It is an insulator, but can be converted into a good conductor by doping with materials (“dopants”) that provide either extra electrons to the conduction band or extra holes to the valence. As an allotrope of carbon diamond is composed of an element from the Group 4 of the Periodic Table. Therefore, to provide spare holes the dopant can be provided from Group 3 (for example, boron, aluminum, or gallium), and to provide spare electrons the dopant can be an element of Group 5 (for example, antimony, arsenic or phosphorous). Impurity atoms from Group 5 have 5 valence electrons and can produce negative-type (n-type) semiconductors by contributing extra electrons. By doping a pure semiconductor (“intrinsic semiconductor”) one creates an extrinsic semiconductor, that is one which is doped. In general, the doped semiconductors have much enhanced conductivity, because the dopant has provided many charge carriers; in the case of diamond it provides them all. Boron is an acceptor in diamond (it has one fewer electrons than carbon). Indeed, boron as acceptant dopant gives rise to highly conductive diamond. For nanocrystalline diamond, the conductivity of diamond can be tuned within 11 orders of magnitude, with values ranging between 1 × 10^−9^ and 1 × 10^+2^ Ω^−1^cm^−1^.

**Fig. 14:**
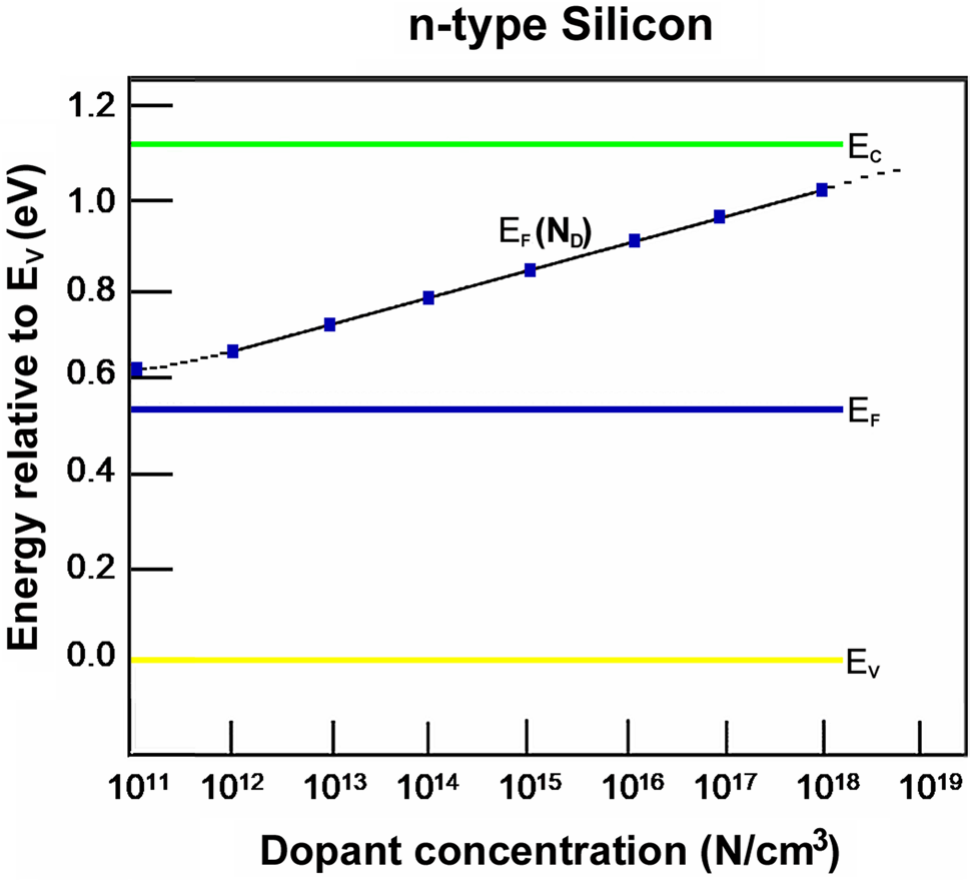
Course of the Fermi level in pure silicon crystals as n-dopant concentration increases. It is customary to base the energy level at zero on the top level of the valence band (*E*_v_): in the case of silicon the conduction band has its lowest energy level close to 1.12 eV: the bandgap in semiconductors always remains fixed, but the Fermi level rises towards the conduction band as the concentration of n-type dopant (which is a donor of negative charge) is increased, and comes close to the lower edge of the conduction band. In the case of doping with a p-type dopant, which is a donor of positive charge

We consider silicon, with its much smaller bandgap. The insertion of a dopant into a crystal of silicon enables electrons to cross the bandgap. The dopant atoms should readily ionize at room temperature. The presence of these ions and their electrons displaces the Fermi band, moving it closer to the lower edge of the conduction band (and, therefore, higher in the bandgap). A new energy level appears in the bandgap, or example at approximately 0.05 eV below the bandgap, this is termed the “donor energy level *E*_D_”. The small gap from *E*_D_ to the conduction band can be crossed by numerous thermally excited electrons (with energies averaging 0.026 eV), so all the donor atoms are ionized. Typical dopant concentrations are in the range from 1 × 10^15^ to 1 × 10^20^/cm^3^, which is many orders of magnitude higher than the intrinsic concentration of carriers due to thermal generation (freeing of electrons from covalent bonds due to lattice vibrations). It is 5–10 orders of magnitude greater than that in the conduction band electron population of intrinsic silicon, which is 1.04 × 10^10^/cm^3^ (see Table 1). Doping with 1 × 10^15^/cm^3^ donor atoms increases the supply of electrons by (1 × 10^15^/1 × 10^10^) a factor 10^5^ = 100,000×; doping with 1 × 10^20^/cm^3^ donor atoms increases the supply of electrons by a factor 10^10^×. Doping with higher and higher concentrations of donor dopant raises the Fermi level higher and higher in the bandgap (Fig. 14). Since 1 cm^3^ of crystalline silicon contains 5 × 10^22^ atoms (Table 1), doping with 1 × 10^20^/cm^3^ donor atoms represents the addition of 1 × 10^20^ atoms to 5 × 10^22^ atoms, which is an addition of (1 × 10^20^:5 × 10^22^) 1:500 = 0.2%. The addition of two parts per thousand of donor dopant increases the supply of electrons in the conduction band by 10^10^×.

**Fig. 15:**
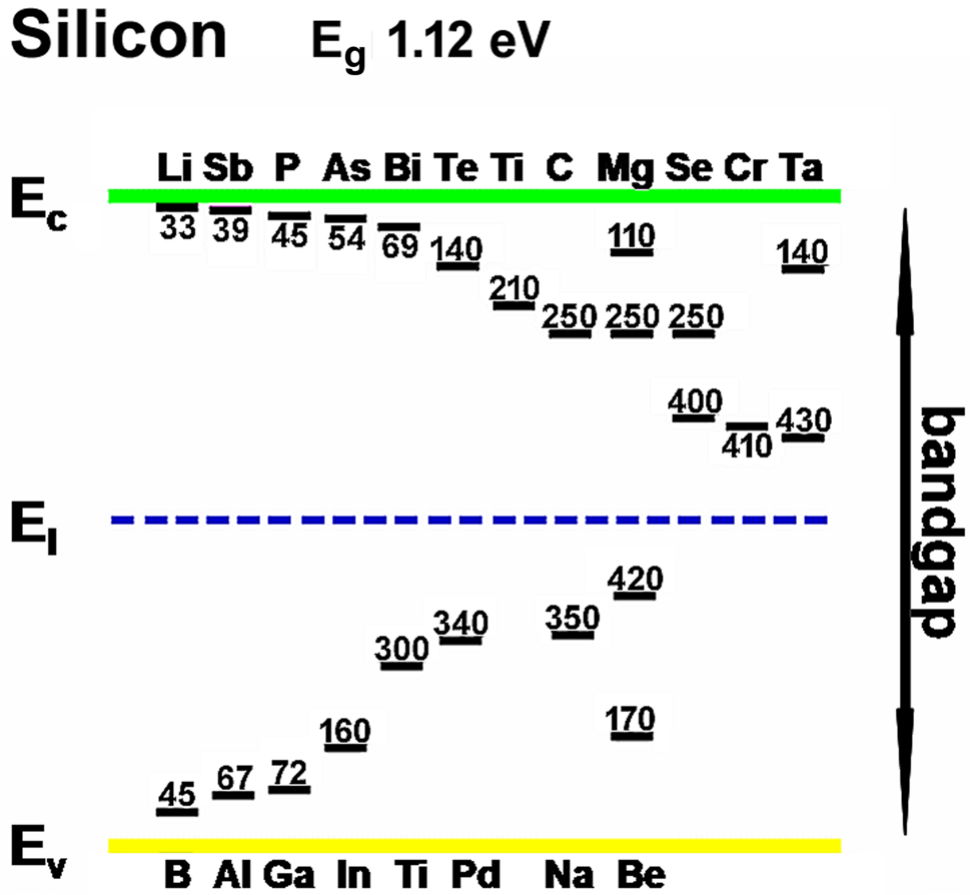
Simultaneous insertion of both donor and acceptor dopants into a silicon crystal. This “co-doping” or “compensated doping” is widely practiced industrially as part of “bandgap engineering”. It is possible to shift the Fermi level to any position within the bandgap, thus rendering shallow energy levels available for electron or hole insertion, or adjusting the Fermi level to access the electron and hole traps deeper in the bandgap. This “tuning” of bandgaps is a source of much proprietary knowledge and is of high economic importance in 2018. The figure shows that any level within the bandgap can be reached by doping with one, two or more dopants

An analogous argument treats the case of the acceptor donor atoms (from Group 3 elements, for example), showing that insertion of low concentrations of such elements into silicon crystals increases the supply of holes in the valence band, and as the concentration of acceptor dopant atoms is increased the Fermi level is shifted downwards towards the upper level of the valence band. Here too, the ideal level is ~ 0.05 eV—here, above the valence band. Figure 15 shows the way in which the bandgap can be structured by the addition of dopants.

Charge carriers obey the Law of Mass Action. This allows calculation of dopant levels. In a non-intrinsic semiconductor under thermal equilibrium, the relation arises from the mass action law and becomes (for low doping): *n*_0_*p*_0_ = *n*_i_^2^, where *n*_0_ is the concentration of conducting electrons, *p*_0_ is the electron–hole concentration, and *n*_i_ is the material’s intrinsic carrier concentration. The intrinsic carrier concentration varies between materials and is dependent on temperature. Silicon’s *n*_i_, for example, is roughly 1.08 × 10^10^ cm^−3^ at 300 kelvins, about room temperature. Consider the case that silicon is doped with an electron donor at *N*_D_ = 1e^16^ cm^−3^. The total number of negative charge carriers is [*n*_i_ = 1e^16^ + 1e^10^] = 1.000001e^16^, so *n*_i_ = ~ *N*_D_. Now the number of holes in the valence band alters to maintain charge neutrality, and n.p = *n*_i_^2^. Since n_i_^2^ = 1.45e^10^ cm^−3^, and the number of holes *p* = *n*_i_^2^/*N*_D_, then *p* = (1.45e^10^)^2^/1e^16^ = 2.1025e^20^/1e^16^ = 21,025 = 2.1025e^4^. Therefore, the concentration of holes in the valence band (2.1e^4^) is (1.45e^10^/2.1e^4^) = 6.90476e^5^ or nearly 1e^6^ times lower than the concentration of electrons in the conduction band. This example illustrates that the fact that if you have increased the supply of electrons in the conduction band by a factor of about 1e^6^ (1e^16^/1e^10^) then you have reduced the supply of holes by about the same factor, namely, 1e^6^.

Indeed, the bandgap of a silicon crystal can be filled with numerous energy levels, by insertion of any of a wide range of dopants, as shown in Fig. 15.

It is evident that the variation of single and mixed dopants, each at a carefully calculated concentration, offers innumerable possibilities for adjustment of the supply of electrons and holes, and can be calculated to allow adjustment at different pressures and temperatures. In industry this fact is exploited daily. In the case of materials destined for clinical use, it will be necessary to avoid use of dopants that show toxic effects in human cells, tissues and organs.

For a concentration of impurities higher than *N*_c_, the conduction electrons are not bound in traps at low temperatures, and the semiconductor exhibits metallic conduction. For phosphorus impurities in silicon, *N*_c_ = 2 × 10^18^ impurities per cubic centimeter. Dopant numbers seem large, but they typically represent only about one dopant atom for each 100,000 silicon atoms. On a percentage basis, a small number of phosphorus atoms will change silicon from an insulator to a metallic conductor. Other semiconductors have similar properties. In gallium arsenide the critical concentration of impurities for metallic conduction is 100 times smaller than in silicon.

From the point of view of nanomedicine, the important interactions of light take place with the electrons in matter. Electrons within a sample of matter may be so tightly bound that they cannot escape from their atomic nuclei. Valence electrons involved in the forming of interatomic bonds are too tightly bound to escape. Mechanical disturbances such as thermal fluctuations may eject a small number of tightly bound electrons out of their bonds to an atom and cause the electrons to enter a freely mobile condition in a higher energy band, the “conduction band”. The number of electrons involved in such cases is very small, as described above. A typical metal has one or more conduction electrons in each atomic unit cell, a semiconductor may have only one conduction electron for each thousand unit cells, and an insulator may have one conduction electron per one million or one trillion unit cells), and if it is zero all the electrons remain in the “valence band”. In that case an electric field may push the electrons and displace them slightly, but cannot cause them to accelerate, or even to move far: a state of strain arises, because the field works upon the material, but the field does not cause electrons to migrate, so no current flows. Such a material is a non-conducting dielectric and will be discussed below, because it allows us to study fundamental aspects of light–matter interactions.

If a small number of electrons is less tightly bound, or if thermal fluctuations can eject them into the conduction band and they do not remain in traps but can move freely, then an electric field can cause electrons to migrate and a current can flow. Materials, in which the normal state is non-conductive, but is rendered conductive by providing free electrons, are known as semiconductors and can conduct electricity under some circumstances and not under others. Semiconductors have been of great interest in nanotechnology, because they respond strongly to size constraints, as will be discussed below. If a material has a copious supply of freely mobile electrons that migrate easily when pushed by an electric field, it will conduct electricity strongly, and the field will do little work on the material itself. Such a material is known as a conductor, and metals such as copper, aluminum, silver and gold provide good examples of this type of matter. The freely mobile electrons can respond to electric fields in ways that are of great interest for nanotechnology, as will be discussed below.

### Metals

A typical metal has one or more conduction electrons in each atomic unit cell. We consider here only the monovalent metals for simplicity, and will focus on the “coinage metals” copper, silver and gold. The metals all have one filled shell and one outer s-electron and since the outer orbits overlap the s-electrons are delocalized. The nuclei appear as positively charged ion cores surrounded by a sea of conduction electrons. In conductors (i.e., metals), electrons only partially fill the valence band and the valence and conduction bands are very close or overlap, thus electrons become conductive (free) very easily. The Fermi level does not appear in a bandgap, instead the valence and conduction bands overlap and the Fermi level lies in well-populated states. In a metal or semimetal, the Fermi level is inside of one or more allowed bands (Fig. 9): electrons do not need to cross an energy gap to enter the conduction band. In a metal, semimetal or degenerate semiconductor, the Fermi level lies within a delocalized band, with many energy nearby that are thermally active and readily carry current; since these bands are made of valence orbitals they are often referred to simply as the “valence band”. The bandgaps in a metal’s band structure are not important for low energy physics, since they are too far from the Fermi level. Metals, having a large supply of freely mobile electrons, between factors 10^8^–10^16^ more than semiconductors (see Table 1), conduct electricity strongly and the electric field does little work on the material itself (compare this case with insulators). Metal s conduct electricity far better than intrinsic semiconductors such as pure silicon, by factors ~ 10^10^ (see Table 1). Metals have been extensively studied and can be characterized by a wide range of experimental techniques. In particular the states near the Fermi surface can be assessed using cyclotron resonance, magnetoacoustic, high-field magnetoresistance, and anomalous skin-effect measurements. Important features of their band structure can be obtained from soft X-ray emission and absorption measurements, and this opens the way to understanding detailed data from optical absorption and reflectivity measurements. Photo-emission studies provide information on densities of states and on electron mean free paths and energy loss per collision, over a wide range centered on the Fermi level (Berglund 1964). For completeness we state the conductivity ranges for the three types of material:

$${\text{Metals:}}\;\sigma> 10^{5} (\varOmega \;{\text{m}})^{ - 1}$$

$${\text{Semiconductors:}}\; 10^{ - 6} < \sigma < { 1}0^{ 5} (\varOmega \;{\text{m}})^{ - 1}$$

$${\text{Insulators:}}\;\sigma < \, 10^{ - 6} (\varOmega \;{\text{m}})^{ - 1} .$$

## Conclusions on energy and materials

The intensive research on transistors and LEDS and photovoltaics that is currently a striking feature of the industrial landscape will create many concepts and develop many techniques and materials that could be of the greatest assistance in the development of nanomaterials for use in medicine. It is not possible to peruse the literature relevant to photoluminescent nanomaterials without encountering new research and data from both industrial and academic sources, and that on an almost daily basis. The quantum–mechanical background in this area has become all-pervasive.

The technologies to measure material properties including quantum ones, and mathematical models based on the Schrödinger equation to calculate them for comparison with the measured values are now several decades old and extensively documented. Therefore, trial and error is no longer a rational procedure in nanotechnology. Reliable and well-tested methods exist for both the measurement and the calculation of the optical properties of both pure and doped materials. The emission from such well-understood materials can be both strong and also tunable across a wide spectral range. This is a fruitful field for research.

There remain these three: bandgap excitons, quantum confinement, and plasmons. For these, and for one or two other types of materials that exhibit prominent quantum effects, a reasonable terminology could be “quantum materials”. In this review we will distinguish between “quantum nanoparticles”, for which the above texts have prepared the background, and more normal nanoparticles that are familiar worldwide and still constitute a large proportion of nanomaterials development today.

This review will consider photoluminescence technology in carbon allotropes as a major topic in the review.

## Carbon allotropes

Allotropy or allotropism is the property of some chemical elements to exist in two or more different forms, in which atoms of the same physical element are bonded together in a different manner, for example, the allotropes of carbon include diamond, graphite and graphene, and others which are discussed below.

### The carbon allotrope diamond: sp^3^ hybrid orbitals

**Fig. 16:**
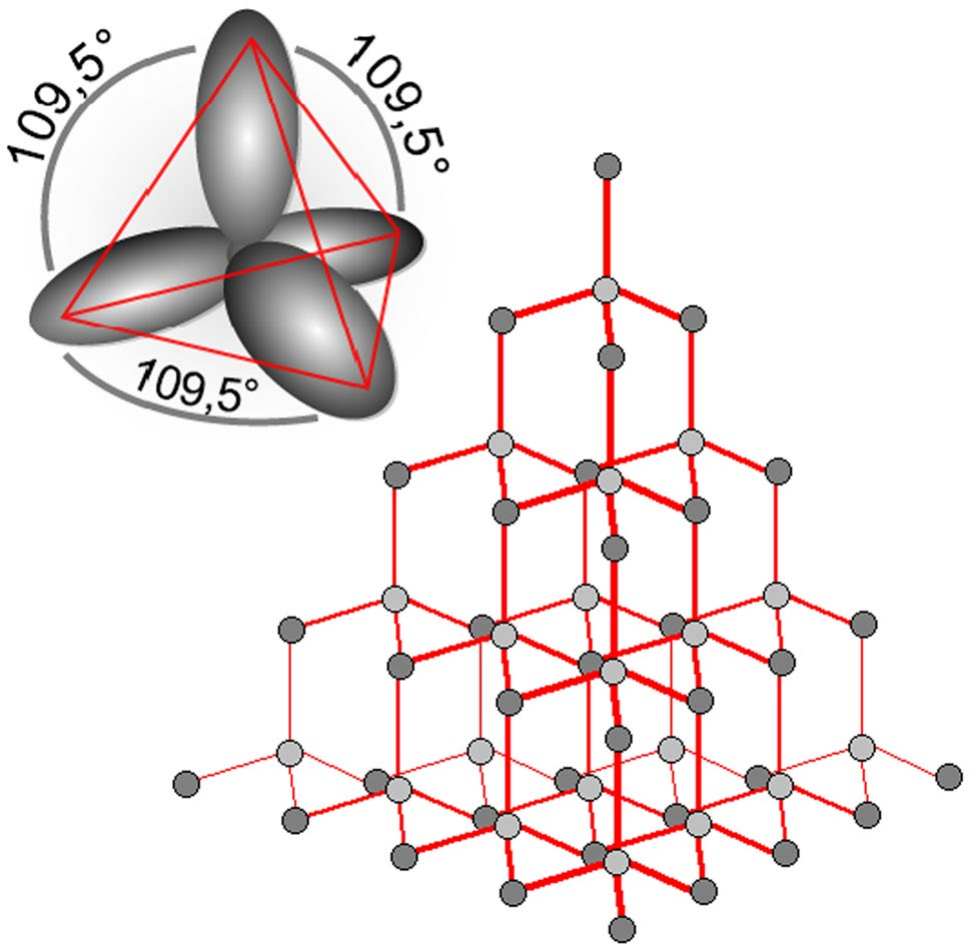
Diamond. The rigid three-dimensional cage-like crystal structure is formed by the four sp^3^ bonds of each carbon atom, linking the carbon atoms in a regular three-dimensional structure built of tetrahedrons; all the carbon atoms and their bonds are identical. No electrons can be promoted into the conduction band without disrupting covalent bonds. The bandgap is, therefore, wide (see Table 2) and undoped diamond is a good insulator

Carbon has the electron structure: **1s**^**2**^**2s**^**2**^**2p**^**2**^. One of the two 2s shell electrons can hybridize with either two or three of the four 2p electrons, giving sp^2^ or sp^3^ hybrid orbitals, respectively. Carbon has the ability to form two stable bonding configurations (sp^2^, sp^3^) with different bond geometries (planar, tetrahedral). In sp^3^ hybridization the 2s orbital mixes with all three of the 2p orbitals, forming four sp^3^ orbitals arising from each carbon atom; this is diamond. The carbon sp^3^ orbitals form four bonds which are directed towards the corners of a regular tetrahedron. The resulting three-dimensional network (diamond) is extremely rigid and, therefore, a hard material (Fig. 16).

The crystalline structure formed entirely from identical covalent bonds does not yield electrons easily, because this would require the breaking of covalent bonds. Diamond, therefore, has a wide bandgap (see Table 1) and is an insulator. It requires doping with Group 3 or Group 5 elements to inject charge carriers, but this doping allows its bandgap to be engineered successfully to tailor its properties to those of a semiconductor. In 2018 this is an active area of research in both academia and industry.

The carbon allotrope graphene: aromatic carbon structures and sp^2^ hybrid *π*-orbitals.

**Fig. 17:**
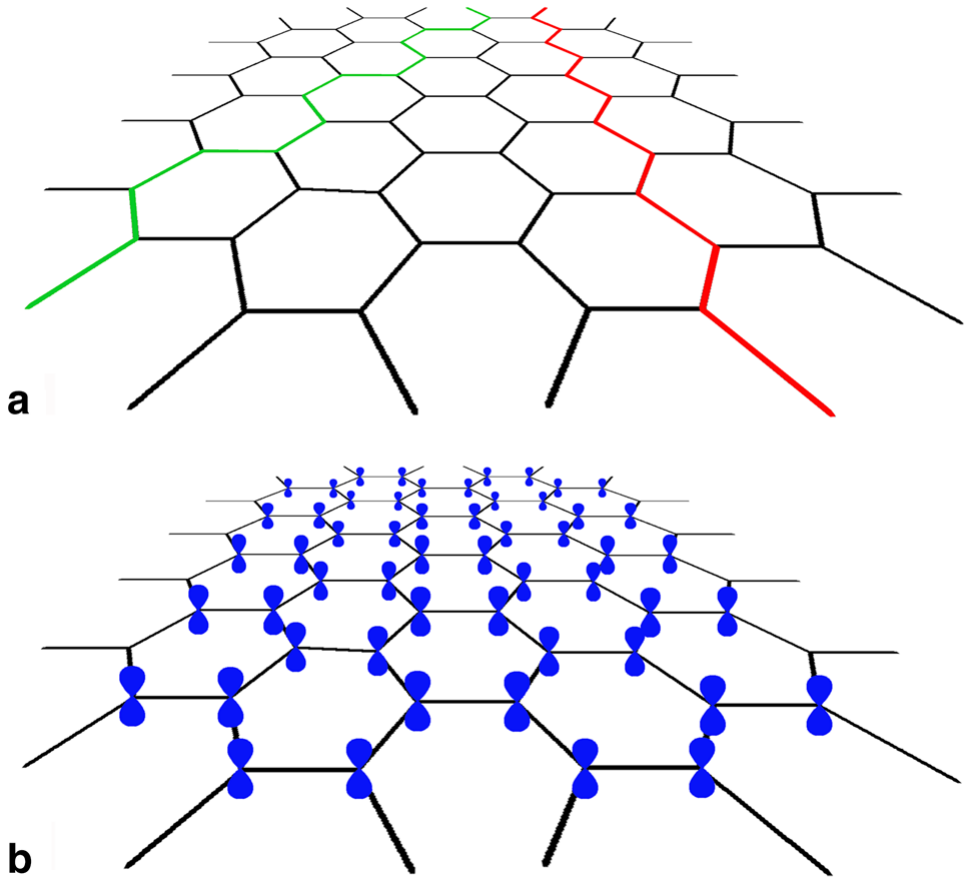
**a** Planar structure of graphene, with zig zag and armchair orientations highlighted in red and green, respectively. The neighboring carbon atoms in the lattice link via sp^2^ hybrid bonds leaving a single electron per atom to protrude vertically on both sides of the sp^2^ sheet.** b** The *π*-electron clouds above and below the graphene sp^2^ sheet: each dumb-bell represents a single z orbital with its node (i.e., zero charge) at the plane of the carbon atom nucleus in the graphene sheet and the orbital lying on both sides of the sheet. The *π*-electrons exchange rapidly with lateral neighboring electrons and form layers of fast-moving electrons both above and below the plane of the lattice

As noted above, carbon’s electron structure: **1s**^**2**^**2s**^**2**^**2p**^**2**^ allows sp^2^ hybridization, in which one of the 2s shell electrons hybridizes with only two of the three available 2p orbitals to form a total of 3 sp^2^ orbitals. Covalent bonds between carbon atoms of such a backbone are formed by three *sp*^*2*^ hybridized orbitals and one non-hybridized orbital, which is commonly denoted as *pz* (Vollhardt and Schore 2010). Figure 17 shows the sp^2^ hexagonal (honeycomb) lattice typical of a sheet of graphite (Saifuddin et al. 2013). The result is graphene, a flat sheet linked by sp^2^ orbitals (Fig. 17) to form a plane regularly tessellated by benzene-type hexagonal rings and, thus, aromatic in nature. In graphite, each carbon atom is connected to three carbons (120°) in the *xy* plane.

On each of the carbon atoms one non-hybridized *p*-orbital remains, the *pz* orbital, oriented in the *z* axis. It overlaps sideways with adjacent *pz* orbitals on the neighboring atoms to form a system of delocalized weak *π* bonds which each have a *π*-bonding molecular orbital and a π* anti-bonding molecular orbital. The resulting shared molecular orbitals are often referred as an extended *π*-system. The non-hybridized *pz* orbitals, therefore, provide two electron clouds, one above and one below the plane of the graphite lattice (Fig. 17). In these clouds the electrons are spatially delocalized: they belong to the whole *π*-system, and no longer to specific carbon atoms. A *π*-system can be extended over an effectively infinite extent, or may be restricted to just a part of it, forming a conjugated segment as discussed in the context of polymers, below. As shown in Fig. 17, the two electron clouds have a zero point (a “node”), where they meet in the plane of the lattice sheet, so there is no electron density at the atomic nucleus.

The origin of the conduction and light emission in these materials lies in the aromatic structure. An aromatic hydrocarbon implies alternating single and double bonds, i.e., conjugation, between the carbon atoms in a ring. In the case of carbon, which has one full S orbital and two half-full P orbitals, quantum mechanisms calculated as the Linear combination of Atomic Orbitals (LCAO) allows for combinations of S and P such that upon bonding, some or all of the electrons involved will hybridize into orbitals which are said to have S character and P character, as described by the sigil sp^n^ with n indicating the ratio of P to S character. Benzene is the simplest of the aromatic hydrocarbons. Each carbon in the hexagonal ring has four electrons to share, one for the hydrogen, two for the neighboring carbon bonds and one extra to freely share for a double bond with its neighboring carbon atoms. In more physical terms, the in-plane C–C and C–H single bonds, σ-bonds, are formed by electrons in so-called sp^2^ orbitals, where the remaining electron occupies the out-of-plane *p*z orbital which due to hybridization with a neighboring *p*z orbital forms a *π*-bond. Since the *π*-bonds are out of plane with respect to the atoms, these orbitals can interact with each other freely, and become delocalized. The out-of-plane *π*-bond implies that instead of being tied to one carbon atom, each electron is shared by all six carbon atoms in the ring. Thus, there are not enough electrons to form double bonds on all the carbon atoms, but the “extra” electrons strengthen all of the bonds on the ring equally. This is why a benzene ring is smaller and stronger than one would expect from the carbon bonds alone. The delocalization of electrons lies at the origin for the conductive behaviour in this class of aromatic compounds, since in this system the electrons can move freely between the different atoms in the molecules. The light emission in organic materials is the result of an electron in an energetically higher (sub) orbital, excited either electrically or optically, decaying radiatively via the selection rules for electromagnetic transitions down to a (sub) orbital lower in energy.

## Fluorescence

A fluorophore is a molecular component that causes a molecule to absorb energy of a specific wavelength and then re-remit energy at a different but equally specific wavelength (Sauer et al. 2010). The limitations of fluorescence, therefore, reflect the capacities of molecules to emit light. For one or two generations of microscopists the upper limit of excitation was considered to lie at ~ 700 nm (~ 1.8 eV), and the upper limit of emission was considered to lie at ~ 1000 nm (~ 1.24 V). This was due to the presence in the fluorophores of the low-lying excited singlet and triplet energy states that absorbed photons but also increased the chemical reactivity of the fluorophores. This reduced the molecules’ thermal and photochemical stability as a result of reactions with, e.g., solvent molecules. Furthermore, the small energy difference between the fluorophores excited and ground states increased the likelihood that excited electrons would relax non-emissively, without radiation of photons. Stable and bright organic fluorophores were considered to absorb and emit in the wavelength range 300–700 nm (Sauer et al. 2010). Classical fluorescence imaging, for example, in microscopy, was one-photon imaging.

*One-photon photoluminescence* Upon absorbing a photon of excitation light, electrons may be raised to a higher energy and vibrational excited state, a process that takes only a femtosecond: 1 × 10^−15^ s (Kong et al. 2012). During the subsequent picosecond (1 × 10^−12^ s), the excited electrons may lose some vibrational energy to the surrounding environment and return to what is called the lowest excited singlet state. From that state the electrons can “relax” back to the ground state with simultaneous emission of fluorescent light. A basic parameter of fluorescence is the fluorescence excitation cross section. One-photon absorption spectra are well documented for a wide range of molecules. Conventional organic fluorophores (e.g., fluorescein, rhodamine), applied extrinsically, have one-photon absorption cross sections in the range 1 × 10^−16^/cm^2^ (Xu et al. 1996b).

*Two- and multi-photon photoluminescence* Intrinsic fluorophores such as amino acids and vitamin derivatives, have extremely low absorption cross sections, orders of magnitude lower than typical extrinsically added fluorophores (Xu et al. 1996b). For these fluorophores (such as some amino acids, NADH, serotonin, or vitamin derivatives, typical fluorescence microscopy would not raise the fluorescence signals above the noise due to autofluorescence, and two-photon fluorescence was introduced to enable the distribution of these weak emitters in living cells to be studied by use of longer optical wavelengths that do not excite autofluorescence. These weak emitters have absorption cross sections in 2-photon microscopy that are close to 1 × 10^−50^ cm^4^ s/photon, approximately 1 GM unit, so that the excitation light needs to be intense (Maiti et al. 1997; Williams et al. 1999) and is often pulsed to provide extremely high power (Zipfel et al. 2003). The peak intensity is many orders of magnitude higher than in normal single-photon excitation, close to 10^11^ W/cm^2^ in routine 2-photon laser scanning microscopy; some applications require as much as 10^12^ Wcm^2^ (Xu et al. 1996b). The resulting application of energy amounts to about 10 mW (10^−3^W), smaller by almost 10^14^ order of magnitude. For functional imaging doses at 20 mW/µm^2^ (a few mW at high N.A.) with a beam dwell time of ~ 1 µs are considered safe (Bennett et al. 1996; Williams et al. 1999). This is because the laser is switched off between pulses, that is: most of the time. The photon fluence in this technique corresponds to a peak photon flux density at the geometric focal point of approximately 3 × 10^30^ photons cm^−2^ s^−1^ (Xu et al. 1996a). Such high fluxes are achieved only at the focal point of a high-N.A. microscope objective, and for this reason two- and multi-photon photoluminescence is not applicable to a context measured in centimeters, such as the human body. The excellent upconversion characteristics of some of the photoluminescent materials to be discussed below are, therefore, not relevant for our theme.

### The interactions of light with non-conducting matter

*Permittivity of pure materials* Consider first a neutral atom placed in an external electric field: it will experience no net force. If the atom is dielectric, however, the electric field will work to disturb and displace the charges within that atom. The field generated by a positively charged source displaces the positive charges within the atomic nucleus a small distance in the same direction as the field, and it displaces the negative charges in the electron cloud a small distance in a direction opposite to that of the field. The atomic nucleus will move in the direction of the electric field until the external force on it is canceled by the force exerted on the nucleus by the electron cloud. In sum, the field pulls the opposite charges apart. The electrons are much lighter than the protons and are, therefore, shifted further than the protons. This deformation of the charges within the atom is known as “polarization”, it creates “dipoles”. So long as the field is present the displacement of the charges is maintained. Upon removal of the field the charges return to their equilibrium positions; in the case of the electrons in the atom, this requires the same time as the original displacement, approximately 10^−15^ s. If the field now reverses its direction, as occurs in light waves, the positive and negative charges (protons and electrons) are pulled apart in the opposite direction. Thus the displacements of the charges follow the oscillations of the field, though with a slight lag time. This lag is briefest in the case of the displaced electrons, and will be discussed further below. Displacement of electrons relative to the atomic nuclei, away from their equilibrium positions in the atoms, is known as “electronic polarization”. In the same way, molecules impacted by an electric field are also stretched by the field, as just described for atoms, so that an electric field can polarize not only atoms but also molecules. Thus in non-conducting materials an electric field strains the electronic configuration of the material, doing mechanical work to pull and stretch the proton–electron bonds, and the material stores charge energy so long as the field is applied. An analogy to the stretched bonds would be the stretching of a metal spring, which stores mechanical energy in the form of strain. As compared to the permittivity of free space, which is defined to have the value 1, electronic polarization is associated with permittivities of 2–3.

Consider next the ionic bonds present in a crystal lattice such as that of sodium chloride. In any material containing ionic bonds, an electric field does mechanical work to stretch the lengths of these bonds. The positive and negative ions are moved apart until the ionic bonding force stops this displacement. As in the neutral atom, this change in length produces a polarization in the unit cell of the crystal; since the polarization here is due to the relative displacements of oppositely charged ions, we speak of “ionic polarizability”. It is comparable to electronic polarization, but the negative charges involved are not electrons but much larger and more massive anions. The work done is, therefore, much larger. Sodium chloride has a relative permittivity of 5.9. Since the ions are more massive than electrons they have more inertia and their response to alterations in the field is slower: their lag time in following an oscillating field is, therefore, much longer than in electron polarization, and is discussed below.

The two cases considered above involve neutral atoms, molecules, and ionically bonded crystals. Consider the third case now in which molecules are intrinsically charged, having positive and negative ends and, therefore, comprising “dipoles”. The interaction of an electric field with matter can form new dipoles in neutral materials as described above, but in many cases the material itself contains permanent dipoles which are present before the electric field impacts upon it. One especially well-studied case is liquid water, which is composed of molecules that contain an angle of 104.45° between the asymmetric bonds coupling the oxygen and hydrogen atoms. At the tip of this angle is the oxygen atom with its negative charge and at the outer side of the angle are the two positively charged hydrogen atoms. This is a polarization built into the water molecule and it is present permanently. When an external electric field impacts such a material, the distance between the chemically bonded positive and negative charges within each permanent dipole remains constant, but the orientation of the dipoles rotates. The external electric field rotates many of the molecules through the angle necessary to align them with the field. The rotational force (torque) that twists the molecules involves much more work than the polarization of electrons or of single anions, because it takes place against the local viscosity of the surrounding molecules. This “orientation polarization” is easier to imagine in the case of a liquid, in which rapid motion of molecules and collisions between them causes the electrically dipolar molecules to point randomly in all directions and thus cancels the net effect of the charges to zero, resulting in an electrically neutral liquid. In solids the molecules or ion lattices are also agitated thermally, resulting in small displacements; consider the motion of pairs of people on a packed dancefloor. Therefore, in solids equally as in liquids or gases, the electrical alignment of charge distributions is disturbed continuously by movements of the surrounding molecules, resulting from thermal agitation. Because the rotation is not instantaneous, orientation polarization requires much longer than the electronic or ionic polarizations described above, and the work which the field must due to achieve (partial) alignment depends both on the strength of the field and also on the degree of thermal agitation, that is: it depends on the temperature of the material (electronic and ionic polarizations are not temperature-dependent). It also depends on the chemical environment. The work done on the dipolar molecules can be large; the permittivity of water has the value 81.

*Dielectric breakdown* A strong electric field pulls charges further apart than a weak field. Very strong electric fields can pull electrons entirely away from the positively charged nucleus, thus ionizing the atoms and allowing conduction flow of free electrons; this breakdown of the insulator requires strong electric fields, however. It needs a force of 13.6 eV to ionize hydrogen by pulling away its single electron, 11.3 eV to remove the outer electron to ionize carbon, 13.6 eV to ionize oxygen, and 24.6 eV to ionize helium. Reference to Fig. 3 shows that these energies are far higher than those of the electric fields in visible light. Photons with an energy of 13.6 eV have wavelength 91 nm, in the UV part of the spectrum, and photons with energy 11.3 eV have wavelength 110 nm, also in the UV. These wavelengths are in the “hard” region of the UV spectrum (which stretches from 10 to 380 nm), so it is unlikely that breakdown of dielectric properties will be met with in nanomedical research.

*Frequency dependence* As noted above, materials cannot polarize instantaneously in response to an applied field. Mathematical descriptions of polarization are, therefore, complex functions of time. Each form of polarization has a characteristic time lag. In the case of electronic polarization, the induced dipoles are small and can, therefore, respond rapidly if the electric field varies in strength and direction, so they can follow an oscillating field even at high frequencies. Electronic polarization can occur even at frequencies as high as 10^15^ Hz = 10^9^ MHz, which is light with wavelength 300 nm, in the UV (Fig. 3). At such high frequencies, only electronic polarization can occur, and this is the most interesting form of polarization for researchers in nanomedicine. The more massive positive and negative ions can follow an oscillating electric field only at lower frequencies, so ionic polarization occurs at frequencies below 10^7^ MHz, which is light with wavelength 30 µm, in the infrared spectrum. The rotation and orientation of molecules can only follow relatively slowly oscillating fields, at frequencies below 10^5^ MHz, which is light with wavelength 3 mm, in the microwave part of the spectrum. Orientation polarization occurs throughout the lower frequency range, down to 1 MHz, which is light with wavelength 300 m, radio wave part of the spectrum. As shown in Fig. 3, each polarization mechanism operates up to a limiting “cutoff frequency”, above which it disappears. As frequency increases, the slow mechanisms drop out in turn, leaving the faster ones to contribute to *e*′. The loss factor (*e*_r_″) will correspondingly peak at each critical frequency. Because permittivity indicates the strength of the relation between an electric field and polarization, if a polarization process loses its response, permittivity decreases. At frequencies above the ultraviolet, none of the polarization mechanisms can function, so permittivity decreases to the value *ε*_0_ in every substance, that is, to the permittivity of free space.

*Permittivity of mixed materials* Mixtures of materials may contain mobile positive ions which can migrate through the material when an external electric field is applied. If a mixture contains large surfaces, for example, the surfaces of grains embedded in a matrix, the ions will migrate as far as such a surface (or “interface”) and become trapped there, resulting in a type of polarization known as “interfacial polarization”. Since interfacial polarization arises from the migration of charge over long distances, the work done by the field on such mixed materials is orders of magnitude larger than that one in pure materials, and the resulting permittivities are far higher. Figure 3 shows that values of several thousands can be obtained in mixed materials. If nanoparticles are embedded in matrices they provide immensely large surfaces for trapping ions, and there is a growing literature describing the applications of nanotechnology in photovoltaic applications, for example, in solar cell design in which high buffer layer permittivities are beneficial (Crovetto et al. 2017).

## Photoluminescence in inorganic semiconductors

Semiconductor materials are small bandgap insulators. The defining property of a semiconductor material is that it can be doped with impurities that alter its electronic properties in a controllable way (Jones 1991), and see above in extenso. Because of their application in the computer and photovoltaic industries—in devices such as transistors, lasers, and solar cell—the search for new semiconductor materials and the improvement of existing materials is an important field of study in materials science. Most commonly used semiconductor materials are crystalline inorganic solids. These materials are classified according to the periodic table groups of their constituent atoms. The interaction of a small bandgap semiconductor to produce either heat or photoluminescence is sketched in Fig. 10.

The size of the energy bandgap determines the rate at which electrons from the valence band enter the conductance band due to thermal influences, see above. It can be measured by observing the transparency of the material to different wavelengths of light, because the photons with less energy than that needed to cross the bandgap cannot interact with the material and, therefore, pass through it: the material is transparent to these photons. As photons of higher and higher energy are tested an energy level will be found at which the material absorbs them and becomes opaque: the energy of these photons matches the size of the bandgap. Thus a semiconductor that is transparent to far infrared light becomes opaque as the wavelength shortens to 870 nm, which corresponds to energy of 1.42 eV (see Fig. 3): this material has a bandgap of 1.42 eV; Table 1 shows that this corresponds to the bandgap of gallium arsenide. Calculation using the equation *λ *=* hc*/*E* shows that silicon (bandgap 1.12 eV) loses transparency at wavelengths shorter than 1107 nm, germanium (bandgap 0.67 eV) loses transparency at wavelengths shorter than 1850 nm, and diamond (bandgap 5.47 nm) loses transparency at wavelengths shorter than 226 nm.

## Presentation and discussion of some “quantum nanoparticles” made of glowing materials, with a sharper focus on the allotropes of carbon

The following text will examine photoluminescence arising from materials which are insulators and can be doped to become conductors (such as diamond and silicon). In these cases the photoluminescence arises in ways that are well modeled in calculations based on the Schroedinger equation. In the case of the carbon allotropes the photoluminescence mechanisms remain a topic of discussion in 2018 and much remains to be learned about them. These materials are the basis of nanoparticles that we term “quantum nanoparticles” in this review, because they do not glow as a result of attaching a fluorochrome to them. They glow from their entire surfaces and the cores, if they have cores, contribute significantly to the glowing from the surface. Since no chemistry is involved in causing these nanoparticles to send light signals they can be more simply designed and their production standardized than materials that are not intrinsically photoluminescent. Only in the case of carbon dots might there be a challenge in devising synthesis protocols that generate highly standardized nanoparticles in terms not only of size but also of their internal chemistry.

## Photoluminescence in metals—plasmons, nanoshells

All metals show plasmon formation. When a metal nanostructure is illuminated with electromagnetic wave of an appropriate wavelength, its conduction electrons will be driven by the electric field to oscillate collectively relative to the lattice of positive atomic nuclei, the oscillating populations of electrons forming a quasiparticle known as a plasmon (Link and El-Sayed 1999), first named by Pines in the 1950s (Pines and Bohm 1952). Associated with the plasmon resonance is a strong local field enhancement in the interior of the metal nanoparticle. The presence of a dielectric core shifts the plasmon resonance to longer wave lengths relative to a solid nanoparticle made exclusively of the metallic shell material. A high permittivity in the core is advantageous. For a given core radius, a thin shell will have a plasmon peak that is shifted to longer wavelengths relative to a thicker shell. “Nanoshells” formed from the “coinage” or “noble” metals exhibit strong photoluminescence properties due to plasmons (Skrabalak et al. 2008). The concept of nanoshells was developed theoretically in the 1950s. A particle in which the inner core of the particle was glass and the outer core was metal would allow the relative size of the core and shell to be varied and in this way one could tune the wavelength of light that that particle absorbs over a large region of the spectrum. Nanoshells were created by Naomi Halas and her team in 2003 and patented in 2004 (Oldenburg et al. 2004). Conventional gold colloids with solid structure, spherical shape, and sizes of 5–100 nm typically display plasmon peaks in the visible region (*λ *=500–600 nm). Only those gold nanostructures having non-spherical morphologies (rods, plates, multipods, and stars) or hollow structure (shells, boxes, cages) exhibit photoluminescence in the NIR spectrum. These gold nanostructures exhibit good upconversion properties (Xia et al. 2013).

## Silicon dots and cornell dots

Cornell dots (“C-dots”) consist of a core about 2 nm in diameter, containing several dye molecules, enclosed in a protective silica shell, which in turn is coated with polyethylene glycol (PEG). This core–shell architecture can be varied in size, both 8 nm and 25 nm diameter being noted in the literature. When exposed to near-infrared light, the C-dots fluoresce. Silica (silicon dioxide) has a wide bandgap of 8.9 eV (*λ* = 139 nm) (Table 1). Silica with this bandgap is fused quartz glass, and is transparent until deep in the ultraviolet spectrum: it cannot absorb photons from the visible spectrum nor from most of the ultraviolet spectrum. Thus unless the shell is doped, the fluorescence arises solely from the enclosed dye molecules. The doping can be carried out using organic molecules or metals (Wolfbeis 2015), and photoluminescence then occurs with emission wavelengths range 300–1000 nm, with a trend towards nanoparticles (NPs) possessing long-wave (> 600 nm) emissions (Mader et al. 2008; Saleh et al. 2011). Fluorophores can also be attached to the outer surfaces of the silica dots, and possible applications involve attachment of lanthanides, rendering the dots capable of upconversion (reviews: Wolfbeis 2015; Mader et al. 2008; Saleh et al. 2011). The resulting nanoparticles do not bleach and show narrow emission bands and can be prepared to emit a large assortment of colors. The silica shell, essentially glass, is chemically inert and small enough to pass through the body and out in the urine. For clinical applications, the dots are coated with polyethylene glycol so the body will not recognize them as foreign substances. The safety and ability to be cleared from the body by the kidneys has been confirmed by studies in mice. This may not extend to malignant lesions: C-dots cause ferroptosis in starved tumour cells but not in healthy cells. C-dots targeted to melanoma (Chen et al. 2018) were entered into clinical trials for use in intraoperative imaging of sentinel nodes in melanomas (Bradbury et al. 2013).

## Photoluminescence in graphene

### Graphene quantum dots

*History* Long the subject of theoretical considerations, graphene QDs could only be prepared as a result of technical advances. Stabilization of the graphene was integral to these new techniques: unstabilized graphene forms graphite. A long history of creating large aromatic sheets by fusing small aromatic compounds encountered challenges due to the low solubility of large graphene sheets. One solution was to attach flexible side chains, separating the graphene sheets by pushing them apart. A simpler process was found that begins with carbon fibers and, by processing at relatively low temperatures (80–120 °C), generates large amounts of blue, green and yellow luminescing graphene QDs. These are smaller than 5 nm diameter and their size can be varied by regulating the temperature at which they are created (Peng et al. 2012). Soon after, similar processes were applied to create graphene QDs from rice husks (Wang et al. 2016d) and papaya powder (Wang et al. 2016b). New technical developments in creation of graphene QDs continue to be made: in 2018, during the preparation of this review, reports appeared describing how orienting two graphene sheets at different angles to one another (“twisted bilayer graphene”) creates a new family of materials in which insulator properties or superconductivity can both be explored (Mele 2018; Cao et al. 2018a, b). As work on bilayer graphene proceeds, novel states of graphene may be found, but this review will focus on the simpler case of single graphene sheets.

### The graphene sp^2^-bonded sheet

*Structure* The structure of crystalline graphene is shown in Fig. 17. Although the plane lattices of graphite are rich in delocalized *π*-electrons they do not have the energy band structure characteristic of semiconductive crystal lattices (discussed above). As a perfect *π*-conjugated single sheet, bulk graphite in pure form consists of sheets that are effectively infinite carbon networks which lack electronic bandgaps. An infinite sp^2^ sheet, therefore, does not photoluminescence: exposing it to light or electricity does not cause it to emit light (Allam and Sarkar 2011). Carbon does indeed absorb light strongly, but it dissipates the absorbed energy by producing phonons or by increasing its temperature. Photoluminescence requires radiative recombination of surface-confined electrons and holes at their surfaces (Sun et al. 2006; Cao et al. 2012b); this in turn requires a bandgap in the electronic structure, which single-layer graphene does not have (Dresselhaus and Dresselhaus 2002). To achieve photoluminescence from graphene, it is necessary to disturb the regular lattice of its bonding linkages and thus to break its symmetry (Abergel et al. 2010). There are several ways to do this. One is to cut the graphene sheet to create an edge and reduce its size. The presence of edges between any intermingled sp^2^ and sp^3^ regions in the impure sheets has a similar effect. It is also possible to dope the graphene (as discussed above for semiconductor materials) and thus engineer a bandgap. Derivatizing the graphene chemically creates bandgaps. Finally, twisted bilayer graphene generate bandgaps in a coupled system of two sp^2^ sheets (Mele 2018; Cao et al. 2018b). Each of these several types of disturbance disrupts the electronic structure of the graphene lattice, imposing local strains on the regular orbital linkages. These types of disturbance may occur singly, or they can occur mixed within a single structure. We consider the major disturbance types next.

*Size of graphene sheet* In large sp^2^ sheets the bandgap is very narrow and the *π* electrons can flow freely among the nearly empty energy states of the conduction band. Graphene QDs are zero dimensional, and graphene has zero effective masses for charge carriers (Li and Yan 2010; Geim and Novoselov 2007; Geim and MacDonald 2007). The electrons, therefore, have mobilities which are quasi-relativistic: the Fermi velocity in graphene has a value of *v*_F_ ≈ 10^6^ m/s (Ponomarenko et al. 2008), which leads to some exotic physical phenomena and remarkable properties not seen in other materials (Geim and Novoselov 2007; Geim and MacDonald 2007). A major result is that the exciton Bohr radius in a graphene lattice is infinite in size, so that quantum confinement occurs in graphene sheets of any finite size (calculated in Son et al. 2006; Ponomarenko et al. 2008; Li and Yan 2010; Brus 2010). For example, for a graphene QD with a diameter of 2.0 nm, the columbic term *E*_C_ ≈ 0.8 eV, representing the bond binding the electron to its hole, is much larger than in CdSe or CdS QDs (Scholes and Rumbles 2010), resulting in distinct excitonic features that can be observed spectroscopically at room temperature (Li and Yan 2010). The size of the sheet quantum-confined the bandgap. In large sp^2^ sheets the bandgap is very narrow, but small sp^2^ sheets are quantum-confined and the bandgap is large; if the sheet size is less than the Bohr radius, photons can cause exciton-based photoluminescence. For sp^2^ sheets of diameter less than 30 nm) the experimental behaviour is completely dominated by quantum confinement, as shown by the onset of quantum chaos calculated from “chaotic Dirac billiards” (Ponomarenko et al. 2008). At sheet sizes smaller than ~ 10 nm photoluminescence can occur. The absorption spectrum of sp^2^ photoluminescence depends on the size of the sheet, acquiring new absorption characteristics as the sheet size is reduced below 10 nm (Pan et al. 2010). The bandgap determines the precise energy of the emitted photon, and in turn depends on the size of the sp^2^ sheet: pure sp^2^ sheets emit from the deep UV (235 nm) to the near infrared (1000 nm) as their size is varied from 0.46 nm to 2.31 nm (Sk et al. 2014). Quantum confinement of conjugated *π*-electrons in an sp^2^ carbon network can be tuned with high sensitivity by varying sheet size shape and edge configuration (Sk et al. 2014; Zheng et al. 2015).

*Bandgap* In view of the unique electronic properties of graphene, a two-dimensional single-layered sheet in which the electrons are free and quasi-relativistic (Son et al. 2006), the lack of bandgaps is seen as uniquely advantageous in applications such as nanoelectronics: many applications would benefit from the predicted unusual electron transport properties of a defect-free extended delocalized aromatic carbon system. On the other hand the sp^2^ sheet presents challenges to any community more interested in graphene’s optical properties. To obtain excitons it is essential that the electronic structure has an energy bandgap (Dresselhaus and Dresselhaus 2002). There has, therefore, been intense research into ways of opening a bandgap in sp^2^ carbon sheets (Abergel et al. 2010). The simplest way to introduce a bandgap into an extremely large sp^2^ sheet is to cut it (Pan et al. 2010). The edge has semiconductor properties, because it disturbs the periodic lattice structure. As shown in Fig. 17, there are two orientations in an sp^2^ sheet, termed “zig-zag” and “armchair”; Radovic and Bockrath proposed that free zig-zag sites are carbene-like, most commonly with a triplet ground state (Zheng et al. 2015; Peng et al. 2012; Fang et al. 2011; Pan et al. 2010; Ponomarenko et al. 2008; Radovic and Bockrath 2005), whereas free armchair sites are carbyne-like, usually with a singlet ground state (Pan et al. 2010; Ponomarenko et al. 2008; Radovic and Bockrath 2005). The edge of an sp^2^ sheet aligns preferentially with the zig-zag configuration. Localized *π* electrons from the aromatic structures stabilize the carbenes through σ–*π* coupling (Pan et al. 2010; Radovic and Bockrath 2005; Fang et al. 2011; Pan et al. 2010; Baker and Baker 2010). In the carbenes there are carbon atoms that possess too few bonding partners to fill their valences, and their unpaired electrons act as spatially fixed “free radicals” and are known as “dangling bonds”. The edge of a carbon sp^2^ sheet is, therefore, rich in dangling bonds. These do not support radiative emission (photoluminescence). “Passivation” of the sheet edges is required, neutralizing the dangling bonds by attaching hydrophilic organic moieties such as carboxylic or hydroxyl groups to them; this permits radiative emission (photoluminescence) to occur. Stated simply, the passivation stabilizes emissive surface energy traps (Myung et al. 2003; Morello et al. 2007; Impellizzeri et al. 2012). Carbenes at passivated sp^2^ edges possess suitable bandgaps (Peng et al. 2012; Pan et al. 2010; Fang et al. 2011), allowing radiative recombination of excitons to generate visible fluorescence. An sp^2^ sheet with edges can be considered a zero-gap semimetal. However, its bandgap is different to those of semiconductor quantum dots. As noted above, their high exciton coulombic attraction plays a much larger role than in semiconductor quantum dots (Li and Yan 2010), rendering it possible to tune the bandgap. Zig-zag edges can emit from the deep UV spectrum to the near infrared (Baker and Baker 2010; Pan et al. 2010; Sk et al. 2014; Li et al. 2011a, b, 2012a, b, c; Ananthanarayanan et al. 2014).

Cutting a graphene sheet into small pieces creates large edge perimeters per area. The same effect can be achieved by creating sp^2^ islands in other types of lattice; this is the case in carbon dots, see below. The islands are commonly created by reducing graphene oxides (Cao et al. 2012a, b). In addition, many different graphene derivatives have been prepared and tested, differing from pure graphene in their structure or in their chemical composition (Kozák et al. 2016). It should be noted that oxidized graphene QDs are no longer technically graphene which is defined as a form of hydrocarbon (Wolfbeis 2015). Both the size and shape of graphene QDs can be precisely controlled by stepwise organic synthesis (Yan et al. 2010). For example, ruthenium-catalyzed cage-opening of C60 molecules produces well-defined graphene QDs (Lu et al. 2011). The shape of graphene QDs can be controlled by varying the annealing temperature and the density of the carbon clusters, usually producing circular or elliptical discs. However, it possible to produce triangular QDs, or QDs shaped as parallelograms, trapezoids, hexagons or mushrooms (Zheng et al. 2015). Graphene QDs are small discs, only one carbon atom thick and, therefore, zero dimensional, with sizes usually smaller than 10 nm diameter, though graphene QDs have been reported as large as ~ 60 nm (Liu et al. 2011a, b). Ribbons can be cut from graphene sheets: termed “graphene nanoribbons”, they are less than 10 nm wide and thus quantum-confined: their edges render them semiconducting (Pan et al. 2010; Son et al. 2006; Wang et al. 2008; Li et al. 2008). The nanoribbons sp^2^ sheets extend over a nanoscale distance in at least one dimension (Kozák et al. 2016).

*Photoluminescence* Due to the *π*–π* transition of C=C bonds (described above), graphene QDs absorb strongly in the short-wavelength UV region (260–320 nm). Like carbon dots, their absorption spectrum extends a tail into the visible range (Baker and Baker 2010; Li et al. 2013). Two excitation peaks are frequently observed from graphene QDs. Their energy difference of < 1.5 eV are due to the *σ*–*π* and *π*–π* transitions associated with the carbene-like triplet state of the zig-zag edges of GQDs (Li et al. 2011a, b, 2012a, b, c; Ananthanarayanan et al. 2014). The luminescence of graphene QDs can be tuned. Green oxygenated GQDs glow blue after replacing carboxyl with alkylamine (Zhu et al. 2012a, b): the nitrogen shortens the emission wavelength (Wei et al. 2014) by withdrawing electrons and reducing the bandgap size (Jin et al. 2013; Ju and Chen 2014; Hu et al. 2013a, b; Li et al. 2011a, b; Sk et al. 2014). Graphite QDs can be tuned to glow from blue to yellow by adjusting their functionalization with amines (Tetsuka et al. 2012). The red emissions of graphite QDs can extend into the infrared (Baker and Baker 2010).

*Quantum yield* Without altering size or shape of the graphene QDSs, chemical reduction or doping with nitrogen reduces bandgap size and raises the photoluminescence quantum yield significantly (Jin et al. 2013; Li et al. 2011a, b, 2012a, b, c; Zhang et al. 2012a, b; Qian et al. 2013; Zhu et al. 2012a, b; Sk et al. 2014; Ju and Chen 2014; Hu et al. 2013a, b), though it blueshifts the emission. Oxidation of the QDs redshifts their emission (Sk et al. 2014; Hu et al. 2013a, b; Zhu et al. 2012a, b; Sun et al. 2013).

*Upconversion* Graphene QDs have excellent upconversion properties (Li et al. 2010a, b; Salinas-Castillo et al. 2013; Yin et al. 2013; Jia et al. 2012; Lee et al. 2013a, b; Zhu et al. 2012a, b), which can be further improved by doping them with nitrogen (Li et al. 2012a, b, c; Wang et al. 2014a, b). Absorption cross sections as high as *σ* = 39,000 GM and 48,000 GM were reported, which are comparable to those of CdSe/ZnS QDs (50,000 GM) (Liu et al. 2013; Wei et al. 2014).

## Carbon nanotubes

The (controversial) history of nanotubes began in 1952, when Radushkevich and Lukyanovich showed clear images of them (Radushkevich and Lukyanovich 1952). Nanotubes and their history have been repeatedly reviewed (Scholes and Rumbles 2010; Hirlekar et al. 2009; Cao et al. 2012a, b; Wang et al. 2010b; Saifuddin et al. 2013; Kozák et al. 2016); a general review with coverage of the various synthesis methodologies is presented by Son et al. (2006). A nanotube is a single molecule composed of an sp^2^ sheet rolled up to form a seamless cylinder (Harris 2009) with its circumference formed by as few as ten carbon atoms configured as benzene-type hexagonal aromatic rings, shown in Fig. 17, and see Saifuddin et al. (2013). The thickness of the cylinder wall is only a single carbon atom. The cylinder has a diameter of ~ 0.7 nm, which is so small that it can be considered zero dimensional in diameter (Wang et al. 2004). It is, therefore, zero dimensional in two spatial directions. The third dimension, represented by the length of the cylinder, can extend for variable distances up to 18 cm (Javey and Kong 2009), that is up to ~ 1.3 × 10^8^ longer than the diameter (Hirlekar et al. 2009) and in this dimension the nanotube is of significant size; the nanotube is thus considered as a one-dimensional structure (Anazawa et al. 2002). The nanotube, comprising millions of carbon atoms, exhibits the flexibility of the carbon atom in forming an endless variety of structures, each with its array of particular, specific and important properties. As counterpoints to the nanotube, which consists purely of carbon and hydrogen, one can point to the polypeptides of proteins or to the strands of oligonucleotides such as DNA, or to the cytoskeletal tubules and filaments, all of which are extended fibrous forms based on carbon chemistry but with vastly different properties and organizing a wide array of the lower molecular weight elements into nanomachineries capable of fulfilling particular teleonomic functions. In this 75th year after publication of Erwin Schroedinger’s seminal publication “What is Life?” (Ball 2018) these comparisons bear a certain poignancy. Unlike the other one-dimensional carbon fibrous structures named, however, nanotubes are far from soluble in water. Nanotubes can enclose other nanotubes, creating multiwall carbon nanotubes (MWCNTs) (Hirlekar et al. 2009), but here we focus on single wall carbon nanotubes (SWCNTs). Carbon nanotubes are to be distinguished from carbon fibers, which are not single molecules (Hirlekar et al. 2009; Saifuddin et al. 2013).

*Bandgap* Carbon nanotubes are strongly quantum-confined. Since they are one-dimensional the nanotubes exhibit periodic boundary conditions around the circumference, and these give rise to a series of narrower sub-bands instead of one wider electronic energy band (Jamal 2014). The energy difference between the two lowest of these is the bandgap of the nanotube (Jamal 2014). The curvature (dependent on diameter) and chirality of the nanotube affects the bandgap and these and related effects render predictive calculation difficult for nanotubes (Jamal 2014). However, optical spectroscopic measurement of bandgap energies (eV) validate the theoretical calculations (Sfeir et al. 2006; Telg et al. 2006). The values of the bandgaps are found to fall from ~ 2 eV for nanotubes of 0.5 nm diameter to ~ 0.4 eV for nanotubes of 3 nm diameter (Jamal 2014). The excitons generated in the nanotubes are delocalized for several nanometers along the nanotube’s circumference (Maultzsch et al. 2005). For one-photon excitation chirality effects affect the excitons (Maultzsch et al. 2005). Their excitons, of both Wannier–Mott and Frenkel types, have large (0.4–1.0 eV) binding energies which are almost one-fourth of the band-gap energy (Maultzsch et al. 2005).

*Photoluminescence* When illuminated with excitation wavelength 532 nm, bare nanotubes emit infrared photoluminescence in the form of several sharp peaks (“quasi-singularities”), each associated with a particular energy sub-band, arising from electron–hole recombination at the band edge. The emission peaks lay in the energy range 0.8–1.1 eV (*λ* = 1130–1549 nm) (Lefebvre et al. 2004). In nanotubes there can occur “dark excitons” that may reduce the quantum yield of the photoluminescence (Zhao and Mazumdar 2004). Carbon nanotubes, in which every atom is at the surface of the nanotube, are influenced by environmental factors such as electric and magnetic fields, ambient pressure, and external chemical environments, and the authors reporting the infrared emissions consider that isolation from the environment may be a factor in obtaining nanotube photoluminescence (Lefebvre et al. 2004). The passivation of carbon nanotubes enhances the intensity of their photoluminescence, as is also the case for other carbon-based sp^2^-type materials (Cao et al. 2012a, b). Passivation is, however, likely to alter the photoluminescence mechanisms of the nanotubes and thus modify the exciton nature of the emission.

## Fullerenes

Fullerenes are an allotrope of carbon that after decades of scepticism (Osawa 1970) was identified in electron microscopical images (Iijima 1980) and a little later as carbon 60- and 70-atom peaks in mass spectrometry. In 1985 fullerenes were synthesized, winning a Nobel prize (1996) for introducing a new allotrope of one of the most important of the elements. Shortly thereafter fullerenes were identified in soot and as a component of interstellar dust clouds. By now, non-carbon fullerene-type molecules configured as cages have been synthesized from a range of other elements including silicon and boron, and from sulfide compounds of tungsten, molybdenum, titanium and niobium (Iddo and Yaffe 2006). Synthesis using sooting flames now produces fullerenes in large amounts, measured in tons (Takehara et al. 2005). The fullerenes have been frequently reviewed, see, for example, Jeong et al. 2012; Wang et al. 2016c; Yeshchenko et al. 2018).

Each icosahedral C_60_H_60_ hydrocarbon fullerene cage is a single molecule formed from carbon atoms arranged in 6-membered or 5-membered rings; since each carbon atom is bound to three other carbons the cage can be modeled mathematically as a trivalent convex polyhedron with only pentagonal and hexagonal faces (Bašić et al. 2017). An infinite number of different fullerenes can exist, but the most stable arrangements do not have adjacent pentagonal rings. The classical isomers with 60 carbon atoms are constructed with 12 pentagonal and 20 hexagonal faces and their properties vary significantly, depending on the distribution of the pentagons. The 70-carbon fullerenes have 70 carbon atoms arranged as 25 hexagonal and 12 pentagonal rings making a total of 37 faces with a belt of 5 hexagons at the equator. Fullerenes with 72, 76, 84 and 100 or more carbon atoms are usually also obtained from syntheses, and purification is a challenge.

The presence of the pentagonal rings prevents formation of normal bonds between the carbon atoms: C_60_ molecules have two bond lengths (1.37 and 1.46 Ångstroms). The bonds between two hexagons are normal double bonds and are shorter than the bonds between a hexagon and a pentagon. The average bond length is 1.44 Ångstroms. The 3 carbon–carbon bonds made by each carbon atom to three others are sp^2^ hybridized. As described above, sp^2^ bonding leaves each carbon atom with one electron that can delocalize as a *π* electron, so that a cage of *n* carbon atoms has *n* electrons free to delocalize over the whole molecule. C_60_ avoids double bonds in the pentagonal rings, and this results in poor electron delocalisation. It, therefore, behaves as an electron deficient alkene, and reacts readily with electron-rich species; in water it becomes an anion. In graphene sheets, which are planar, the sp^2^-hybridized carbon atoms are at an energy minimum, and bending the bonds to form a spheroidal cage causes angle strain. To reduce this strain the C_60_ fullerenes readily undergo chemical changes which convert the sp^2^-hybrid bonds into sp^3^-hybridized ones, decreasing the angles from about 120° to about 109.5° and rendering the molecule more stable.

The C_60_ cage is 0.65–0.7 nm in diameter with the wall being one carbon atom thick. The fullerene cage is strongly quantum-confined, it has partially delocalized *π*-electrons and it, therefore, has a bandgap. In the case of C_60_ the bandgap is 1.7 eV (*λ* = 730 nm)/1.86 eV (*λ* = 667 nm), and C_70_ fullerenes have a bandgap 1.77 eV (*λ* = 700 nm)/1.57 eV (*λ* = 790 nm) (Rabenau et al. 1993). The hollow space inside the fullerene cage can be filled with a metal or small molecules to create “endohedral fullerenes”, which have been studied almost, since fullerenes were discovered. The result is often a reduction in the bandgap; a review in 2013 gives reduced bandgap values of ~ 0.86 eV (~ 1440 nm) in the cases of neodymium, praseodymium and cerium caged inside the fullerene C_88_ (Rivera-Nazario et al. 2013). The fluorescence quantum yield of fullerenes is low: ~ 5 × 10^−4^, due to efficient intersystem crossing (Nascimento et al. 2007).

There is little evidence for fullerene toxicity (Lalwani and Sitharaman 2013). However, after thorough and extensive review those authors state that there are good grounds to assess each new fullerene individually and not to conclude a new form is safe, because it is a generic fullerene. In view of the complex strains caused in the cage’s chemical reactivity by the permutations of possible pentagons, hexagons and (rarely) pentagons in the molecule lattice, and in view of the possibilities of charge transfer across the single layer of carbon atoms that form the fullerene wall, this is a pertinent consideration.

## Carbon dots

*History* Carbon dots (or carbon nanodots), surface-functionalized small carbon nanoparticles (Sun et al. 2006; Cao et al. 2007; Bourlinos et al. 2008a, b; Tian et al. 2009; Hu et al. 2009; Peng and Travas-Sejdic 2009; Jiang et al. 2009; Wang et al. 2010a, c; Li et al. 2010a, b; Qiao et al. 2010; Li et al. 2011a, b), were first reported in 2006 (Sun et al. 2006). These colorless and non-luminescing nanoparticles only become light emitters after being passivated with colorless simple organic molecules that themselves also do not emit light. Passivated carbon dots, however, are brightly photoluminescent. They emit blue light if carboxyl groups are attached to them and yellow light if hydroxyl groups coat their surfaces. At their discovery, 12 years ago, this bright photoluminescence was an enigma, addressed in the first publication reporting them (Sun et al. 2006). It emerged that these nanoparticles are toxicologically and ecologically “friendly”. They can be made by simple “green” techniques, and their photoluminescence qualities rival those of semiconductor QDs. They show great future promise in several technical fields for use as sensors, transducers, electron collectors (Soni 2016), white light generation (Zhu et al. 2018a, b; Yang et al. 2018) and as signal emitters. Their development in nanomedicine is paralleled by developments in several adjacent technological fields and synergies between the research fields are possible. They have, therefore, been the object of numerous investigations, generating a large, detailed literature which has grown exponentially and now includes thousands of publications. Since theirs is a rapidly developing story, we note here a few of the many reviews, noting their dates of publication: in 2008: (Sun et al. 2008a); in 2009: (Yang et al. 2009a), in 2010: (Baker and Baker 2010; Wang et al. 2010a, c); in 2011: (da Silva and Gonçalves 2011; Anilkumar et al. 2011); in 2012: (Zhang et al. 2012a, b); in 2013: (Luo et al. 2013; Zhang et al. 2013; Qu et al. 2013; Zhu et al. 2013); in 2014: (Wang and Hu 2014; Wei et al. 2014); in 2015: (Zheng et al. 2015); in 2016: (Sun et al. 2016); in 2017: (De and Karak 2017; Wang et al. 2017); there are numerous others. We note the admirable research contribution coming from the start from China. To give a taste of the luxurious proliferation of potential raw materials used to create carbon dots we append here an incomplete list, it includes: soot (Allam and Sarkar 2011; Liu et al. 2007; Vinci and Colón 2012; Baker and Colón 2009), carbon nanotubes (Bottini et al. 2006; Zhou et al. 2007), carbon fibers (Vinci et al. 2015), graphene sheets (Pan et al. 2010), activated carbon (Qiao et al. 2010; Li et al. 2011b; Dong et al. 2010), graphite (Sun et al. 2006; Hu et al. 2009; Cao et al. 2007; Zhao et al. 2008; Lu et al. 2009; Zheng et al. 2009, 2011; Wang et al. 2011) various small organic molecules (Zheng et al. 2015) such as acetic acid (Fang et al. 2011), amino acids (Wei et al. 2014), citric acid or glucose (Dong et al. 2012a, b), sucrose (Zhang et al. 2010), carbohydrates (Liu et al. 2011a, b; Peng and Travas-Sejdic 2009), C 60 molecules (Lu et al. 2011), and nanodiamonds (Yu et al. 2005a, b; Chang et al. 2008; Wolfbeis 2015). Research on these eco-friendly nanoparticles blossomed from 2014 onwards, extending the syntheses to a further wide range of natural raw-stuffs and ecologically friendly syntheses including: wool (Wang et al. 2016a), flour (Zhang et al. 2015), bagasse (Du et al. 2014), radishes (Liu et al. 2017a), orange juice (Sahu et al. 2012), peaches (Atchudan et al. 2016), coffee grounds (Hsu et al. 2012), garlic (Sun et al. 2016), coriander (Sachdev and Gopinath 2015), papaya (Wang et al. 2016a, b, c, d), carrots (Liu et al. 2017b), potatoes (Shen et al. 2017), waste frying oil (Hu et al. 2014), waste biomass (Suryawanshi et al. 2014) and pigeon feathers, eggs and manure (Ye et al. 2017); the list becomes longer almost every day. The number of protocols reported for preparation of carbon dots is almost equally long, see below. The result has always been the same: carbon dots do not show photoluminescence unless they first coated with organic materials that themselves have no luminescent properties.

The history of carbon nanodots has been driven by their combination of favourable attributes, including small size, size- and wavelength-dependent tunable photoluminescence, photostability and almost entirely blink-free luminescence emission, resistance to photobleaching, high capacity for upconversion, electroluminescence, simple chemistry of bioconjugation, biocompatibility and low toxicity, chemical inertness, abundance of raw materials, availability of numerous simple and rapid synthesis protocols that can be applied cheaply on a large scale and frequently involving only one step. For a review see (Wolfbeis 2015; Baker and Baker 2010). It has become clear that the synthesis protocol is often a major determinant of carbon dot properties, and given the variety of synthesis protocols it is likely that the composition of the dots would vary significantly. We therefore consider this next.

*Composition* Most carbon-based quantum materials with the exception of diamond (see above) contain little sp^3^-bound material and are rich in sp^2^-bound sheets and amorphous carbon (Fang et al. 2011; Ray et al. 2009; Hu et al. 2009; Baker and Baker 2010), neither of which exhibits photoluminescence. A range of analytical techniques, 13C NMR, selected-area electron diffraction, FTIR measurements, high-resolution transmission electron microscopy, infrared/Raman spectroscopy, and X-ray diffraction, reveal the crystalline regions in carbon dots (Wei et al. 2014; Fang et al. 2011; Ray et al. 2009; Hu et al. 2009; Baker and Baker 2010). A small carbon dot, 2–3 nm in diameter, may contain a small number of sp^2^ regions, stacked (Zheng et al. 2015) and the largest containing fewer than 100 aromatic rings; for an extensive discussion of such small sp^2^ regions see (Eda et al. 2010), which is written in the context of graphene QDs (see above). The carbon dots, therefore, contain abundant edge sites (Zheng et al. 2015). The borders of sp^2^-bound crystalline regions are formed largely of zig-zag sp^2^ sheet edges, their “dangling” carbene-like ground states (Pan et al. 2010; Radovic and Bockrath 2005) stabilized by aromatic structures (see Fig. 17) via *σ*–π bonding (Radovic and Bockrath 2005) These edges are semiconducting, and will be discussed below. The X-ray diffraction patterns revealed polyaromatic structures containing oxygen and, if nitric acid was used in preparation of the dots, also nitrogen in the dot cores (Ray et al. 2009); in one version of carbon dots the content of oxygen can be as high as 50% and that of nitrogen as high as ~ 13% (Zheng et al. 2015; Ray et al. 2009). This content of oxygen and nitrogen distinguished the carbon dots from graphene QDs which, by definition, contain neither of these elements (Wolfbeis 2015). The oxygen and the nitrogen are of high importance for the photoluminescence properties of the carbon dots (see below).

*Potentially high heterogeneity of carbon dots* As already evident in the preceding section on the composition of carbon dots, these nanostructures are prepared under conditions which have numerous variables capable of disturbing uniformity of the results, for example, pH, temperature, ionic concentrations, duration and intensity of the oxidation necessary to create carbon dots, type and intensity of passivation, and others. Small difference in the graphitization temperature can result in varying sizes of sp^2^-bonded domains and thus shift the luminescence wavelength to longer or shorter wavelengths (Fang et al. 2011), or incomplete graphitization in the particle’s core may have fallen more or less short of completion, so that small but varying amounts of sp^3^-bonded material may remain in the core (Ray et al. 2009). Some authors have attempted to drive graphitization to completion to obtain quantifiable carbon dots (Cao et al. 2012a, b). The emission spectra of the dots are broad because of the typically large heterogeneity which results from the poorly controllable synthesis processes (Zheng et al. 2015). The frequently mentioned requirement for filtration or chromatography to obtain small and standard-sized dots bears witness to the heterogeneity of the as-prepared carbon dots, even within a single batch (Zheng et al. 2015; Li et al. 2010a, b, c; Zheng et al. 2013; Vinci et al. 2012; Liu et al. 2007; Pan et al. 2012). After separation by gel electrophoresis or by filtration, the much purer populations of dots do indeed differ strongly amongst themselves (Vinci et al. 2012), confirming the heterogeneity of the original batch. Not only size but also chemical heterogeneity are observed. For example, passivated carbon dots bear oxygen-containing carboxylic/carbonyl moieties at their surface and the oxygen content may be at any level 5–50% in weight (Baker and Baker 2010; Cao et al. 2012a, b; Aloukos et al. 2014; Bourlinos et al. 2008a, b). These variations alter the photoluminescence spectra, which may exhibit several characteristic peaks differing in emission peak position and spectral width and shape, revealing the presence of distinct emission sites on the CDs. Furthermore, the intra-batch heterogeneity will be less than the inter-batch heterogeneity. These heterogeneities hinder analysis of the photoluminescence mechanisms of the dots, by causing inconsistencies in the observational data. In 2017, it was reported that single-dot spectroscopy demonstrated the presence on a single dot of a range of different emissive sites allowing generation of a whole spectrum of photoluminescence wavelengths from that single dot, and matching in the single dot the excitation-dependent spectra resembling those of the population of dots in the batch (van Dam et al. 2017). It is to be expected that inconsistent data might be generated from even a single dot, or from a two slightly different dots. The presence of multiexponential lifetime decays in the photoluminescence provides a further indication that different emissive sites are present (Baker and Baker 2010).

*Carbon dot photoluminescence* Carbon dot photoluminescence was an enigma when the dots were first discovered, and this enigma has been defined in increasingly more differentiated terms, but has not yet been solved. The evidence presented above provides a background against which the complex relationships within the dots can be considered. The initial observation is of major importance: carbon dots do not glow unless they are first coated with non-glowing oxygen oxygen- and nitrogen-bearing small molecules, but then they glow brightly. Passivation is essential. Their glowing is not disturbed by blinking and they remain stable under many hours of strong illumination (Sun et al. 2006; Zhao et al. 2008; Zhang et al. 2012a, b; Shen et al. 2012a, b; Peng and Travas-Sejdic 2009; Baker and Baker 2010). The dots are semiconductors, they also—and at first sight incongruously—exhibit excitation-dependent luminescence.

*Bandgaps* Carbon dots with their sp^2^-bonded regions possess abundant zig-zag edges (Zheng et al. 2015; Sk et al. 2014), each edge disrupting the crystal lattice regularity and capable of supporting bandgap-determined emission (see above). They exhibit the properties of semiconductors, possessing an energy gap due to the sp^2^ domains in the cores of the dots, together with the confinement effects due to their small sizes less than 10 nm diameter (Cao et al. 2012a, b). Electrochemical luminescence data show that the dots possess an energy band structure similar to that of semiconductor nanocrystals (Zheng et al. 2009); the presence of absorption edges in their absorption spectra is a semiconductive feature (Cao et al. 2012a, b). They are at least partly determined in their emission properties by their size, 2–10 nm (Ray et al. 2009; Sk et al. 2014; Wang et al. 2010a, c), and quasi-spherical shape (Zheng et al. 2015; Ray et al. 2009; Allam and Sarkar 2011; Bourlinos et al. 2011; Baker and Baker 2010); dots much larger than 10 nm diameter are only weakly emissive. Apparently, the conditions for *π*-plasmon photo-emission exist in the dots and the reader expects to find the dots to have broad absorption spectra with narrow (almost monochromatic) emission wavelengths (Michalet et al. 2005; Alivisatos 1996), reflected by tabulated bandgap and emission values in the relevant literature, as found, for example, for nanotubes, see above. However, the photoluminescent properties of C-dots depend on the internal dot structure in a more complicated fashion, and the literature points in a different direction. In particular, the size effect is known to be dependent on whether the core is crystalline or amorphous and does not obey the “particle in a box” phenomenon, which is valid in the case of quantum dots (Zhu et al. 2013). The relevant extensive literature provides two evidences that carbon dots do not exhibit bandgap-mediated fluorescence. The first is that these reviewers have not seen a bandgap size stated for carbon dots, quite unlike the case for nanotubes (see above). Second, we have not seen a emission plot for carbon dots that shows the extremely narrow, almost monochromatic, spike that characterizes the emission spectrum of, for example, classical QDs such as cadmium selenide QDs. Some authors report size-fractionated carbon dots showing size-dependent but excitation wavelength-independent emissions (Baker and Baker 2010; Zhao et al. 2008; Wei et al. 2014; Dong et al. 2013; Yang et al. 2014). However, typical emission peaks for carbon dots are relatively broad, and in many cases are dependent on the wavelength characterizing the absorption maximum of the dots.

*Excitation-dependent luminescence* Excitation wavelength-dependent emission wavelengths were also demonstrated (Sun et al. 2006; Liu et al. 2007; Qiao et al. 2010; Qu et al. 2012; Bhunia et al. 2013). In view of the possibility that the dot core is not heavily involved in photo-emission, some authors speak of the “fluorescence” of carbon dots (Ray et al. 2009; Wang et al. 2010a, c; Xu et al. 1996a). In applying the term “fluorescence” to the dots, an author treats the emission sites at the dot surfaces as fluorophores, the groups within small organic molecules that are responsible for fluorescence emission: which is considered to involve only surface confinement of electrons and holes (Sun et al. 2006; Cao et al. 2012a, b). The dots’ overall optical properties result from competition between emissive sites and non-radiative trap sites on the surface (Li et al. 2012a, b, c). One report described two versions of carbon dots, the version derived from particular basic and aromatic aminoacids showing excitation-independent emission, whereas the version derived from neutral and aliphatic aminoacids showed “traditional” excitation-dependent emission properties (Wei et al. 2014). We return to the question of absorption capacity of the dots below, after discussing the wavelengths and luminescence lifetimes of the dots.

Carbon dots have only one excitation peak, and from this arises the maximum emission. Due to their *π*-plasmon electrons (Xu et al. 2011) the dots absorb mainly in the UV spectrum (260–320 nm) with the major single peak at 270–280 nm (Zheng et al. 2015; Hu et al. 2009; Baker and Baker 2010; Li et al. 2013; Cao et al. 2007; Zhou et al. 2007; Zhu et al. 2009); this peak can be shifted by passivation to the 350–550 nm range (Peng and Travas-Sejdic 2009). The absorption peak tails off into the visible range (Baker and Baker 2010; Li et al. 2013); some authors consider this “red-tail” together with short radiative lifetime to be characteristic of fluorophores (Efros and Rosen 2000). Carbon dots excited by these longer wavelengths in the UV blue, from 330–475 nm, then emit mainly in the blue to the yellow–green, from 440 to 600 nm (Wolfbeis 2015); more rarely, versions are reported that emit in the red and near infrared (Wolfbeis 2015; Zhang et al. 2017). The emission from carbon dots rarely has a wavelength longer than the near infrared (Wu et al. 2013; Yang et al. 2018; Li et al. 2011a, b, 2012a, b, c; Pan et al. 2016; Tang et al. 2012; Lim et al. 2006). One report relates the blue emissions to the intrinsic state of the dots, the green emissions to the C=O surface groups and the red emissions to the C=N surface groups (Yang et al. 2018). Some authors report a pH-dependence of both emission intensity and wavelength on pH value of the surrounding medium (Baker and Baker 2010; Zhao et al. 2008; Liu et al. 2009; Lakowicz 2006).

The photoluminescence decay curves of carbon dots are in the range 1–20 ns (Baker and Baker 2010; Wei et al. 2014; Sun et al. 2006; Zhu et al. 2009), suggesting these arise in normal fluorescence transitions (Tang et al. 2012).

The emission intensities of carbon dots depend strongly on their quantum yield, and this in turn varies widely as a result of the numerous synthesis protocols employed (Lakowicz 2006). Almost every publication reporting synthesis and characterization of carbon dots quotes values for their quantum yields. The original reports on carbon dots reported values below 10% (Sun et al. 2006) and more recent works describe doping and passivating the dots, obtaining values close to 80%. The quantum yield of carbon dots was first reported in the range below 10% but can be increased by attaching electron-donating groups such as diamine, thiol, hydrazide, or alkylamine to hinder non-emissive exciton recombination (Zhang et al. 2012a, b; Qian et al. 2013; Zhu et al. 2012a, b). PEGylation raises the yield further and passivation results in extremely high yields in the range 70–80% (Sun et al. 2006; Shen et al. 2011; Lee et al. 2013a, b; Liu et al. 2012; Dong et al. 2012a, b; Shen et al. 2012a, b; Wang et al. 2013; Anilkumar et al. 2011); quantum yield depends on the degree of passivation (Baker and Baker 2010). Doping carbon dots with sulphur or nitrogen can raise their quantum yields above 70%; this increasing quantum yields by doping is a feature that carbon dots share with graphene quantum dots (Cao et al. 2007; Qu et al. 2013; Dong et al. 2013).

For the purpose of considering the question we posed at the beginning of this review, the input–output characteristics of the carbon dots are of critical importance. The quantum yield is the output side of this: it states which proportion of absorbed photons will be emitted (Lakowicz 2006). To consider the input side it is necessary to know either the absorption coefficient of the carbon dots, or alternatively the absorption cross section. The absorption coefficient can be measured using a UV spectrophotometer and consulting the absorbance spectrum of the material, and is then calculated using the Beer–Lambert law. The absorption cross section σ is then related to the absorption coefficient by multiplying the [absorption coefficient × atomic molar mass (g/mol) × *N*_A_]. This arithmetic requires knowledge of the carbon dot’s weight in Daltons, whether from measurement or, if this poses difficulties, this parameter can be estimated from knowledge of the dot diameter and the lattice spacing of the sp^2^-bonded sheets. It surprises us that the burgeoning literature on carbon dots rarely if ever states their absorption cross sections routinely, rendering it difficult to carry out the arithmetic required to calculate photon fluxes into and out of the carbon dots. There are welcome exceptions, for example, the report of these parameters for nanowires (Protasenko et al. 2006). However, amongst dozens of relevant publications checked by us, the dots are usually described as “bright” and their quantum yields are frequently stated. However, knowledge of the quantum yield is of little use unless the absorption cross section is also stated. The absorption cross section “determines the per-QD photo-excitation density at a given photon fluence, which is a crucial parameter for both spectroscopic studies and QD optoelectronic device design, and for calculating radiative lifetime which serves as a benchmark to understand effects such as modifying the surface chemistry of QDs and to manipulate photoluminescence (PL) quantum yields” (Leatherdale et al. 2002; Yu et al. 2005a, b). Instead of reporting this critically important value, authors usually show graphs with the *y*-axis labelled in A.U. (“Arbitrary Units”), as “normalized” or as “Fluorescence Intensity”. Fortunately, the reader can help herself. The general equation for absorption cross section is given as

$$\delta_{ \hbox{max} } \, = \, 10^{ - 15} \;{\text{cm}}^{2n} \times \left[ {10^{ - 23} \;{\text{s/photon}}} \right]^{n - 1} ,$$

where *n* is the number of photons absorbed.

For one-photon fluorescence imaging, the classical variety, for example, in microscopy, this equation requires insertion of *n *= 1 and then takes the form:

$$\delta_{ \hbox{max} } \, = \, 10^{ - 15} \;{\text{cm}}^{2} \times \left[ {10^{ - 23} \;{\text{s/photon}}} \right]^{0} = \, 10^{ - 15} \;{\text{photons / cm}}^{2} .$$

This result accords well with published absorption cross-sectional values measured for inorganic QDs, for example, “6.2 × 10^−16^ × *R*^3^ cm^2^ at 2.76 eV and 3.15 × 10^−16^ × *R*^1.28^ cm^2^ at the first-exciton absorption peak, with the dot radius *R* in nm”, giving respective values of *δ*_max_ = 6.2 × 10^−16^cm^2^ and 3.15 × 10^−16^cm^2^ for InAs QDs of diameter 2.0 nm (Yu et al. 2005a, b). Other authors state InAs QDs to have absorption cross sections ~ 0.43 × 10^−15^ cm^2^ (Osborne et al. 2004). Silicon QDs are stated to have absorption cross section 1.46 × 10^−14^ cm^2^ at 405 nm excitation (Sangghaleh et al. 2013). For CdSe QDs absorbing at 350 nm the per particle absorption cross section *C*_abs_ (in cm^2^) for CdSe is *C*_abs_ = (5.501 × 10^5^)*a*^3^ cm^−1^, where *a* is the particle radius in cm; this gives: 5.501 × 10^5^ × (2.5 × 10^−7^)^3^ cm^2^ = 8.60 × 10^−15^ cm^2^ for a 5 nm diameter CdSe QD (Leatherdale et al. 2002). On the basis of these data and calculations, carbon dots of diameters near 5 nm may be expected to have absorption cross sections close to 10^−15^ cm^2^ at their maximal absorption wavelengths (see above). This agrees also with the fact that carbon dots have similar absorption cross sections as inorganic QDs for 2-photon photoluminescence (Cao et al. 2007). These data are all in accordance with the results reported in (Zheng et al. 2013).

A further check can be carried out with reference to the literature. In 2012, Cao et al. compared CdSe QDs with (ZnS-coated) carbon dots. The two types of particles were of similar sizes and exhibited similar fluorescence quantum efficiencies, and under the same optical conditions they produced images of approximately the same intensity. The images provide an approximate estimate of the photon flux from each dot, and this was approximately the same for both particle types. The absorption cross section for CdSe QDs has been reported as being 8.60 × 10^−15^ cm^2^ for a 5 nm diameter CdSe QD (Leatherdale et al. 2002). This is adequately close to the value we calculated above, namely, ~ 10^−15^ cm^2^ at the wavelength of maximal absorption. From these considerations we can draw one significant conclusion: that carbon dots are not intrinsically “brighter” than inorganic QDs. It follows that carbon dots could be used in replacement of inorganic QDs in many applications.

*Mechanisms of photoluminescence in carbon dots* The enigma of these mechanisms is to harmonize the semiconductor nature of carbon dots with their fluorescence form of photo-emission. The discussion has focused in part on the relationship between the sp^2^-bonded regions in the dot’s core and the oxygen- and nitrogen-containing groups on its surface (Zheng et al. 2015; Zhu et al. 2018a, b; Ray et al. 2009; Zhu et al. 2012a, b; Wei et al. 2014; Bourlinos et al. 2008a, b; Li et al. 2002; Kozák et al. 2016). Some authors posit two separate mechanisms of emission (Baker and Baker 2010; Sun et al. 2006; Li et al. 2012a, b, c; Cao et al. 2012a, b; Myung et al. 2003; Zheng et al. 2009). A more popular explanation as we sampled it from the literature might be to consider the “*π*-conjugated electron systems in the dot core to as centers for quantum confinement and photo absorption (Wang et al. 2010a, c; Krysmann et al. 2012), while the surface groups provide different vibration relaxation for their excitation-independent emission and large Stokes shift” (Sun et al. 2006; Cao et al. 2012a, b; Li et al. 2002; Baker and Baker 2010; Krysmann et al. 2012; Zhu et al. 2012a, b; Wei et al. 2014; Wen and Yin 2016). In that case, the small size of carbon dots might simply imply that they have a large surface area/volume ratio and thus provide them with a maximal area from which to emit photons (Sun et al. 2006). It may be worth noting here that if the sp^2^-bonded regions in the dot core do play a role in the ultimately fluorescence-type photo-emission, then dot size still plays a significant role: in an sp^2^-bonded sheet with its interatomic distance of 1.42 Å (0.142 nm) (Cooper et al. 2012), then in a carbon dot of 1 nm radius, no atom is further than 7 atoms distant from the surface; in a carbon dot of 5 nm radius (as in Sun et al. 2006), the center-to-surface distance is much larger, at 35 atoms. This increased distance between central core and surface may explain the decreasing brightness as dots grow larger (Ray et al. 2009). For review of the discussion in 2015 see Zhu et al. (2015).

*Passivation* As noted above, passivation is essential for carbon dots to exhibit photoluminescence. It not only permits the dot to glow, it also reduces the effects of non-emissive trap sites, and hinders quenching of the photoluminescence (Wu et al. 2013). The reason is that the surface passivation of carbon dots introduces numerous types of organic groups that can act as emissive traps and that can each be considered as a fluorophore. Passivation cause large improvement of the photoluminescence quantum yield of CDs (Wang et al. 2014a, b). It broadens the emission spectra of carbon dots (Zheng et al. 2015; Wolfbeis 2015), introducing different surface trap sites that cause the *λ*_ex_ dependency (Baker and Baker 2010). Its stabilization of the emissive surface energy traps resembles that occurring in inorganic semiconductor quantum dots or nanocrystals, in which the surface ligands or semiconductor shells surrounding their cores conditions their photoluminescence (Myung et al. 2003; Morello et al. 2007; Impellizzeri et al. 2012). Passivation of several types has been explored by numerous authors (Cao et al. 2012a, b) and it features large in the relevant literature. It provides an important means of rendering carbon dots relevant to the question posed at the beginning of this review, by extending their emission wavelengths into the near-infrared spectrum (Wolfbeis 2015; Zhang et al. 2017). Large organic structures such as PEG or polymers may be particularly useful for this; PEGylation of carbon dots shifts both the absorption and emission maxima towards the red spectrum as the molecular weight of the passivating molecule is increased (Baker and Baker 2010).

*Doping* carbon dots respond to doping in similar ways to graphene-based QDs, see the discussion of graphene QDs above. Nitrogen-doped carbon dots are much more strongly fluorescent (Wolfbeis 2015; Sk et al. 2014; Ju and Chen 2014; Hu et al. 2013a, b; Li et al. 2011a, b). Quantum yields can exceed 70% in such dots (Qu et al. 2013; Dong et al. 2013).

*Upconversion* the mechanism of carbon dot photo-emission is still under discussion, but it does allow the dots to excel in photonic upconversion, the emission of shorter wavelengths upon the simultaneous absorption of two or more longer wavelength photons (Zheng et al. 2015). Like graphene quantum dots (Liu et al. 2013), carbon dots can efficiently up-convert the energy of two or three absorbed long wavelength photons (800 nm) into a shorter wave photon (~ 550 nm) (Zheng et al. 2015; Cao et al. 2007; Li et al. 2010a, b, c; Salinas-Castillo et al. 2013; Yin et al. 2013; Jia et al. 2012). The absorption cross section of the dots is high, and doping them with nitrogen raises it to the highest recorded value for carbon-based nanomaterials, exceeding that of other organic dyes and comparable to that of high performance semiconductor QDs, namely, to 32,000 GM (Kong et al. 2012) 39,000 GM (Baker and Baker 2010; Sun et al. 2008b; Cao et al. 2007), ~ 42,000 (Cao et al. 2007; Sun et al. 2008b), rivaling and surpassing the > 10,000 GM reported for CdSe quantum dots (Pu et al. 2006) and 47,000 GM reported for CdSe/ZnS core–shell quantum dots (Larson et al. 2003) for the same excitation wavelength. (Note that GM units for the special case of 2-photon absorption are named after the woman who first formulated the possibility of multi-photon interactions (Göppert-Mayer 1931). The remarkable capacity for upconversion that is exhibited by carbon dots is mentioned here for completeness, but is of little relevance to our guiding question concerning detection of photons emitted from nanoparticles located within a living human being, because it requires a light flux of high peak intensities, typically 10^20^–10^30^ photons/cm^2^ s for the observation of two-photon absorption (Xu and Webb 2002), a photon flux far higher than that at the surface of the sun. Both the continuous wave and the pulsed laser emissions necessary for 2-photon fluorescence can cause tissue heating and cell and tissue damage (Williams et al. 1999). Furthermore, the intense photon fluxes necessary for this mode of excitation are produced at the focus of lenses in microscopy applications, and imaging of nanoparticles at an unknown distance both from the light source and from the imaging device mandate almost the opposite specification, namely, that a wide area flux of photons of approximately uniform cross-sectional distribution is introduced into the human body.

*Toxicology* In living mice PEGylated carbon dots did not show toxicities across a wide range of indicators (Yang et al. 2009a; Zhang et al. 2013) and were excreted rapidly (Huang et al. 2013; Lee et al. 2013a, b); they did not accumulate strongly in the reticulo-endothelial system. They did not cause cytotoxicity in murine and human cell lines (Yang et al. 2009b; Peng et al. 2012; Zhang et al. 2012a, b; Zhao et al. 2008), even at concentrations far higher than required for bioimaging studies (Ray et al. 2009). For a review comparing carbon dots with alternative materials see Wolfbeis 2015). These results are not surprising, because carbon is a component of living cells and tissues; it is worth noting the physicochemical relationships of carbon dots to soot, diamonds, and other graphitic materials, to all of which humans have been exposed for tens of thousands of years. Today, our exposure to such materials in the form of industrial and domestic emissions is so large as to swamp out any toxicity from carbon dots, for example, see the voluntary self-exposure of subjects smoking cigarettes or handling their collection of diamonds. However, the regulatory bodies will not on account of these considerations provide special allowances to carbon dots.

*Comparison with graphene and with inorganic semiconductors* Graphene QDs are discs of sp^2^-bonded carbon atoms in which no oxygen, nitrogen or other elements are present. The perfect and infinitely large graphene sheet is non-luminescent, and has no emissive traps. In contrast to this, carbon dots contain large amounts of oxygen and nitrogen and incorporate extensive edges with intrinsic bandgap properties. The photoluminescence properties of carbon dots arise from complex relationships within carbon dots that are not possible within graphene sheets. Several reviews focus on mechanistic and phenomenological similarities between graphene QDs and carbon dots (Sun et al. 2006; Cao et al. 2007, 2012a, b). However, the structural distinctions between graphene QDs and carbon dots (Ananthanarayanan et al. 2014) render carbon dots more difficult to functionalize at their surfaces (Wolfbeis 2015). Furthermore, quantum yields from graphene QDs are higher than with bare carbon dots, due to the layered structure and more perfect crystallinity of the graphene QDs (Zheng et al. 2015). The graphene QDs do, however, offer a major advantage from the point of view of the question we posed at the beginning of this review: graphene QDs can emit at wavelengths up to 1000 nm (Zheng et al. 2015; Sk et al. 2014).

C-dots show spectrally broad photo-emission with strong *λ*_ex_ dependency, fluorescent nanodiamonds emit from point defects, particularly the negatively charged nitrogen vacancy site, which absorbs strongly at 569 nm and emits near 700 nm (see above).

Carbon dots require passivation to glow, and this is similar to the case of silicon nanocrystals (Baker and Baker 2010), see below.

Carbon dots emit at approximately the same intensities as semiconductor QDs (see above), but have notable advantages in terms of inherent non-toxicity (Zheng et al. 2015).

## Nanodiamonds

Diamond is an allotrope of carbon. It is an sp^3^-bonded crystal with tetrahedral structure, as shown in Fig. 16. Each of its carbon atoms is joined by sp^3^ bonds to 3 others, so that all available electrons are incorporated into the covalent bonds. The bandgap in diamond is, therefore, wide, see Table 1: it is 5.47 eV (*λ* = 226 nm). Diamond is, therefore, transparent until deep in the UV spectrum. Unless doped, diamond is an insulator. If doped with a Group 3 or a Group 5 element it contains charge carriers (electrons and holes) and in this way its bandgap can be bridged and it becomes a semiconductor. Natural diamond has a long history, but artificial diamonds are now of great interest in a wide range of technical areas. For example, millimetre-sized diamonds doped with nitrogen have recently been reported to provide the first high-power masers functioning stably at room temperature and expected to be of great interest in communications technology (Liu 2018; Breeze et al. 2018). Nitrogen doping introduces negatively charged point defects into the diamond crystal lattice, and allows photoluminescence with excitation at 569 nm and emission near 700 nm (Baker and Baker 2010). The doping can be carried out by nitric acid oxidation (Ray et al. 2009). To obtain uniformly doped diamond has been a significant technological challenge, but this challenge is being successfully faced (Seo et al. 2016.

Diamond as a source of photoluminescence does not photobleach and has low toxicity (Yu et al. 2005a, b). The production of nanodiamonds has been carried out by milling microdiamonds, deposition from vapour, and shockwave processes (Baker and Baker 2010; Krueger 2008a, b, c).

## Conclusions on “quantum NPs”

- The allotropes of carbon include some (e.g., nanotubes, nanodiamonds, graphene QDs) that can be tuned to absorb and emit in the near-infrared spectrum.
- The low intrinsic toxicity of carbon is a solid base from which to plan regulatory acquiescence into nanoparticle design from the beginning, though the necessity of checking each new version will be perennial.
- The easy availability of raw materials for synthesis, in large amounts, favours carbon dots over nanodiamonds but not over carbon nanotubes or graphene QDs. The advantage over QDs fabricated from noble metals is evident.
- There are ecologically friendly “green” synthetic pathways available for graphene, nanotubes and carbon dots, and at least for nanotubes the products can be produced in amounts of tons.
- The photoluminescence properties of the carbon allotropes are comparable to those of inorganic semiconductor crystals in terms of absorption and emission qualities. The carbon allotropes are not burdened with the proven toxicological properties of the inorganic semiconductors.

## Non-quantum NPs

*Liposomes* These consist of an amphiphilic phospholipid bilayer and have sizes from 50 to 500 nm diameter; they are used in diagnosis, imaging and targeted drug delivery. According to the size and number of phospholipid layers one distinguishes between uni-, oligo- and multi-lamellar liposomes (Sanvicens and Marco 2008). They are extensively used in research, some are in clinical trials and in clinical use (Zhang et al. 2008; Shi et al. 2017; Ventola 2017).

*Dendrimers* consist of highly branched, symmetric, monodisperse, spherical polymers, sizes ≤ 15 nm diameter. They have layered structures with a central core, an internal region and numerous terminal groups (Menjoge et al. 2010). They can comprise different solubilities and biological activities. They are used as tissue-repair scaffolds, for diagnosis, imaging and targeted drug delivery via chemical modification of their multiple terminal groups (Sanvicens and Marco 2008). Dendrimers are used in research, but none are in clinical trials nor in clinical use (Shi et al. 2017; Ventola 2017).

*Polymers* are natural (e.g., polysaccharides), or synthetic (e.g., polystyrene) macro-molecules, solid spheres or vesicular capsules; sizes from 50 to 500 nm diameter. They have different physicochemical properties such as solubility, melt viscosity, surface area, packing density, solubility and glass transition temperature; used in biomedicine, electronics, photonics, conducting materials, sensors, pollution control and other environmental technologies (Rao and Geckeler 2011). They are extensively used in research, some are in clinical trials and in clinical use (Shi et al. 2017; Ventola 2017).

*Protein based nanoparticles* Proteins, natural polymers, sizes from 50 to 500 nm diameter, used in diagnosis, imaging and targeted drug delivery. They are extensively used in research, some are in clinical trials and in clinical use (Shi et al. 2017; Ventola 2017).

*Quantum dots* They are semiconductor crystals and exhibit fluorescent behaviour due to quantum confinement effects. They fluoresce strongly at wavelengths which are remarkable due to the extremely narrow peaks in the fluorescence spectra. Quantum dots fluorescing in the visible light spectrum are 2–10 nm in diameter, they form their functional centers from groups II to VI (CdSe, CdTe, CdS, PbSe, ZnS and ZnSe) or groups III to V (GaAs, GaN, InP and InAs) of the periodic system. The cores are coated with a layer of zinc sulfide (Sanvicens and Marco 2008). Quantum dots require to be coated and functionalized to render them soluble, to modulate their processing in physiological fluids, and to permit attachment of drug molecules. Quantum dots potentially could be of great usefulness in medicine, because they show remarkably high quantum efficiencies and resistance against photobleaching. They generate strong, stable fluorescence signals and, therefore, provide excellent contrast agents for imaging (Sanvicens and Marco 2008). However, they may blink; moreover, the core materials forming their essential functional centers are often extremely toxic, and care must be taken to ensure that the contents of these particles cannot leach out into the body. They are used extensively in research, e.g., “Qdot^®^ Nanocrystal” containing a core made of CdSe, CdTe or ZnS and being coated with different polymers (Weissleder et al. 2014), but none are in clinical trials nor in clinical use (Shi et al. 2017; Ventola 2017).

*Magnetic nanoparticles* Ultrasmall particles of iron oxide (USPIOs), spherical, crystalline structures of 10–20 nm diameter, comprise cores of Fe^2+^ and Fe^3+^ ions coated with dextran or polyethylene glycol, allowing surface functionalization for attachment of signalling and/or of therapeutic molecules (Sanvicens and Marco 2008). They provide “negative contrast” in MR images (Stollenwerk et al. 2010). They are used in research, and some are in clinical use (Shi et al. 2017; Ventola 2017).

*Gold nanoparticles* Colloidal gold (Faraday 1857), extensive use as contrast agent in electron microscopy, further use in imaging procedures with bright optical emission at narrow wavelengths (Roth and Berger 1982). Functionalization with, e.g., antibodies as targeting agents, for biological uses gold particles are coated with, e.g., silica, dextrans or polyethylene glycols. They are used in research, but none are in clinical trials nor in clinical use (Shi et al. 2017; Ventola 2017).

### Nanoparticle physicochemistry

The targeting efficiency of current nanoparticles is around 0.7% of injected dose, in other words only 7 out of 1000 administered nanoparticles reach their target cells in in vivo models (Wilhelm et al. 2016). This low efficiency can be explained by the biological conditions in vivo, e.g., the tumour physiology, the presence of barriers, immune system, enzymes, etc., often presenting significant obstacles to the nanoparticles. In addition, the physicochemical composition of the nanoparticles plays a major role. However, after in vivo administration the nanoparticles will encounter a complex biological machinery starting to interact with them: interactions with endogenous proteins, different types of cells, membranes and body fluids. These interactions, and the interactions between the single nanoparticles in vivo are still poorly understood. The unique properties of materials arise at the nanoscale, which implies several orders of magnitude increase in surface area for a given volume of the respective material, thus increasing the proportion of constituent atoms at or near the surface. As many reactions occur at the surfaces of materials, this immense extent of surface area not only affects the reactive chemical but also the mechanical and/or electrical properties of devices. In addition, such small materials are the natural home of quantum effects, which influence their optical, electrical and magnetic characteristics. Quantum confinement of electrons gives rise to sharp tuning of emission wavelengths, which underlies the characteristic properties of quantum dots (Varadan et al. 2008). There is as yet no generally accepted guidance concerning the design criteria for clinically applied nanoparticles, but the availability of research- and pre-clinical-data from a large number of different types of nanoparticles has shown certain tendencies that will aid development of such guidelines (Wittrup et al. 2012; Lane et al. 2015).

*Size* The major criterion for nanoparticles is size. According to their definition nanoparticles should at least have one of their dimensions in the nanometer size range, i.e., below 1 µm: “nanospheres” are defined as so-called “zero-dimensional” materials comprising all their dimensions within the nanoscale; “nanowires*”* or “nanotubes*”* are so-called “one-dimensional” materials with one of their dimensions larger than 100 nm; self-assembled thin films are so-called “two-dimensional” materials with two of their dimensions larger than 100 nm (Murthy 2007). However, biological considerations require the lower range for nanoparticle therapeutics to be 10 nm, as measured in terms of sieving coefficients for the glomerular capillary wall and estimation that the threshold for renal first-pass elimination is approximately 10 nm (Davis et al. 2008). The vasculature inside one tumour can vary enormously with parts of different permeabilities ranging from 4 nm diameter in one part of the tumour, to 200 nm diameter in another part (Lammers et al. 2012). Extravasation from the vascular system into the interstitial space requires nanoparticles to be below 60 nm in diameter. A fair balance between prolonged circulation and extravasation is needed. Research has shown the preferred size range to be between 12 and 50 nm diameter, the optimum being within the size range of IgG molecules (Wittrup et al. 2012). However, nanoparticles in clinical use normally range between 100 and 500 nm diameter (Shi et al. 2017; Ventola 2017). This is too large to vacate the vascular system easily. In addition, large nanoparticles are more likely to be recognized by macrophages eliminating them before they can reach their target. These facts might be some of the reasons why nanomedicine is still lacking real success.

*Dispersity* Size and size dispersity are entangled with each other. Dispersity drastically influences the in vivo reactions of nanoparticles irrespective of their use as imaging or drug delivery agents. The polydispersity index (PDI) is a measure for the uniformity of particle sizes in a mixture and is defined as the ratio of the [population average particle volume based on particle volumes] to [population average particle volume based on particle numbers]: $\overline{{V_{v} }} /\overline{{V_{N} }}$. PDI relates to the normal statistical parameters (mean = *n*, standard deviation = *σ*) according to the formulae: coefficient of variation (CV) =  $\sqrt {PDI - 1}$ and CV = *σ*/*n* (Stollenwerk et al. 2010) A major unsolved challenge in nanomedicine is the production of homogenous, monodisperse nanoparticles with affirmed batch-to-batch reproducibility. Remedial action is normally taken by later use of edging processes, e.g., mechanical milling, laser ablation or arc discharge synthesis. These so-called top–down methods are rather expensive and time consuming rendering them unsuitable for large scale industrial production. In addition, they may disturb the original character of the nanoparticles changing their in vivo behaviour. A better but more sophisticated approach is the so-called bottom–up method, where nanoparticles are formed using atoms or single molecules as source materials. The difficulty here lies in identifying the boundary conditions which form one certain size only. However, nanoparticles formed that way are composed more homogeneously regarding their chemical arrangements (Varadan et al. 2008), and are easier translated into industrial production. A combination of top-down and bottom-up processes might help eliminating the limitations present in either of the methods.

*Shape* Nanoparticles have been designed showing different shapes, e.g., tubes, cages, rods, spheroids, elongates, of which rod-shaped particles seem to have higher delivery efficiency compared to other shapes. The corners and edges are excellent emitters of (infrared) photons. However, none of these shapes has improved delivery to more than 1% of initial dose (Wilhelm et al. 2016). According to this, shape has some influence on in vivo delivery, but to a much lesser extent than size, charge, stability or matrix materials used to prepare the nanoparticles.

*Stability* A crucial feature of well-designed nanoparticles is stability, whether considered in terms of chemical stability and resistance to cleavage by enzymes in the body fluids, or in terms of mechanical and structural integrity to withstand turbulence and shear forces in the blood stream. At the same time, the design should allow for an adequate structural flexibility so that, by amœba-like flexibility, they can cross the endothelium, the interstitial space and finally the epithelium, and enter the target cells without being disrupted. Stability and size influence the circulation time which should be long enough for the nanoparticles to reach their target cells. At arrival and entry into the target cells, the nanoparticles should then be able to disintegrate into smaller units releasing the drug. The particle remnants which have only served as cargo for the drug or imaging agents should be excreted from the target cells via exocytosis, and further via renal or hepatic pathways.

*Charge* Electrical charge on the molecules is a major determinant of how closely two molecules can approach one another, and also dictates the orientation of the molecules undergoing electrostatic interactions. Before considering the role of charge in nanoparticle interactions, a discussion of charge in the early, classical model of colloidal dispersions will be presented. The parameter generally used is the Zeta Potential (*ζ*-potential), well documented in pharmaceutical technology and in other technologies using small particles. It is generally measured to aid adjustment of colloidal dispersions to prevent aggregation of the constituent particles. This potential arises when particles migrate in an applied electric field. The particle migrates faster in the electric field than does the diffuse cloud of counterions surrounding it, so that some counterions at the migration front are sheared off, exposing part of the particle’s electric field: this potential difference is named the *ζ*-potential. The “shear plane” lies between the ion layer immobilized on the surface of the particle and the diffuse counterion cloud: the *ζ*-potential is the potential difference between the bulk solution and the shear plane, or in other words, it is the potential difference between the dispersion medium and the stationary layer of fluid attached to the dispersed particle. If only water is present, that is: if no other counterions are present in the Electric Double Layer (EDL), then the *ζ*-potential is identical with the surface potential of the charged particle (Attard et al. 2000; Khair and Squires 2009). Several methods for measuring *ζ*-potential have been developed, including electrophoretic light scattering and microelectrophoresis, evaluated by use of one of several derivatives of the Smoluchowski equation which has been modified by several authors (von Smoluchowski 1903; Overbeek 1943; Booth 1948; Dukhin and Semenikhin 1970; O’Brien and White 1978; O’Brien and Hunter 1981; Hunter 1989; Dukhin and Goetz 2002). Electroacoustic techniques are also employed, using colloid vibration current or electric sonic amplitude (Babchin et al. 1989; Dukhin and Goetz 2002) as measurement parameter. Differently charged nanoparticles have been produced revealing that positive surfaces interact strongest with macrophages being cleared rapidly, compared to negatively charged agents. Nanoparticles with neutral surfaces are least opsonized, least taken up by macrophages and also least toxic. Whereas charged particles tend to disrupt cell membranes, neutral particles can be endocytosed without disruption. Another phenomenon in vivo is protein fouling which is prone for highly charged nanoparticles. Protein fouling describes the adsorption of endogenous proteins present in body fluids onto the surface of charged nanoparticles (Gref et al. 2000; Cedervall et al. 2007; Lundqvist et al. 2008). This implicates that the original known chemical identity of the nanoparticle changes to an unknown biological identity (Albanese et al. 2014). The pharmacokinetic behaviour of such a nanoparticle cannot be foreseen. Therefore, different coatings have been introduced to camouflage surface charges: ligands containing ethers or hydroxyls as functional endgroups induce an overall neutral surface (Ehrenberg et al. 2009). The most commonly used ligand is polyethylene glycol (PEG) together with its derivatives, which also provide a possibility of attaching targeting groups to the nanoparticle (Torchilin 2006). PEG is a neutral, hydrophilic and relatively unreactive polyether. It can, however, be prepared with different molecular weights, different structures (i.e., linear, branched, linear forked, branched forked) and different terminal functional groups (i.e., for amino-, sulfhydryl-, carboxyl- or hydroxyl-group conjugation), and these derivates can be quite reactive and thus suitable in a range of applications (Roberts et al. 2002). Furthermore, linear and branched PEGs act as steric repulsive molecules offering an additional barrier against protein adsorption (Lane et al. 2015). Ligands containing phosphatidylcholine (anionic phosphate and cationic ammonium), sulfobetaine (anionic sulphate and cationic ammonium) or carboxybetaine (anionic carboxylate and cationic ammonium) are zwitterionic coatings having a balanced distribution of acidic and basic residues thereby appearing as overall neutral surface (He et al. 2008). Nanoparticles coated with either of the above mentioned ligands can evade uptake by macrophages or the reticulo-endothelial system (Abuchowski et al. 1977) and show prolonged circulation time in the blood. However, these ligands also increase the overall radial size by up to 10 nm, and in some cases can induce immunological recognition followed by particle elimination (Lane et al. 2015; Ishida et al. 2006). Following the rule of keeping it simple (see below), the preparation process of nanoparticles should be adapted to create particles possessing a neutral surface intrinsically with no need for further derivatization.

*Matrix material* A major concern in nanomedicine is the in vivo stability of the nanoparticle (see above). The used matrix material strongly affects this stability, and also strongly influences allergenic, immunological and toxicological reactions; however, it less influences the passage the nanoparticle takes in vivo. It has been shown that covalently formed nanoparticles carrying covalently bonded functional groups, i.e., antibodies, imaging agents or drug molecules, are more stable in vivo than nanoparticles being connected via physical mechanisms such as charge or hydrophilic interactions. Protein nanoparticles offer the most versatile possibilities of attaching functional groups covalently such as amino, carboxyl and sulfhydryl groups. Depending on the sub-structure used to create dendrimers they can also possess chemical groups which can be used for covalent functionalization. The same is true for polymers. Liposomes in contrast cannot be derivatized covalently. They are made out of lipids shaping themselves into a bilayered structure with a hydrophilic or hydrophobic core depending on the number of lipid layers, and a hydrophilic surface. They, therefore, can be functionalised via solubility and pH gradients either incorporating drug or imaging molecules into the core, or by adsorbing them onto the liposomal surface. Antibodies are normally covalently linked onto PEG chains, the PEG chains are then built into the lipid bilayer structure of the liposome; however, the anchorage of the PEG chain into the lipid bilayer is not covalent. The stability of non-covalently linked nanoparticles for long-term in vivo circulation is doubtful. Anorganic nanoparticles such as quantum dots, gold or iron need to be derivatized with proteins such as albumin, or polymers to be functionalised. The connection between the anorganic and the organic material is crucial for the stability of the complex. Besides the concerns for in vivo stability covalent linkages additionally allow the creation of special bonds, e.g., amide or ester bonds, which are cleaved by certain enzymes in vivo. Using enzymes to disintegrate nanoparticles can be an additional way of targeting tumour sites. Activity-based protein profiling (ABPP) has already identified some interesting regulators of tumour growth and progression, such as retinoblastoma-binding protein 9 (Bogyo 2010) or APOBEC3B (Periyasami et al. 2015).

## Imaging of glowing nanoparticles in a human body: a calculation

We return to the question posed in the Introduction. We posit a local density of nanoparticles anchored to a location 3 cm deep within a human body and emitting infrared radiation in the “water window” (Zhu et al. 2018a, b). *With which intensity must this collection of nanoparticles emit light to trigger a detector that requires a signal*-*to*-*noise ratio *> *3 to register the presence of the nanoparticle against the background of the body’s infrared background noise? Can we design this nanoparticle, using materials satisfactory to a regulatory authority that acts as Maxwell’s Demon at the gateway to the marketplace?*

**Fig. 18:**
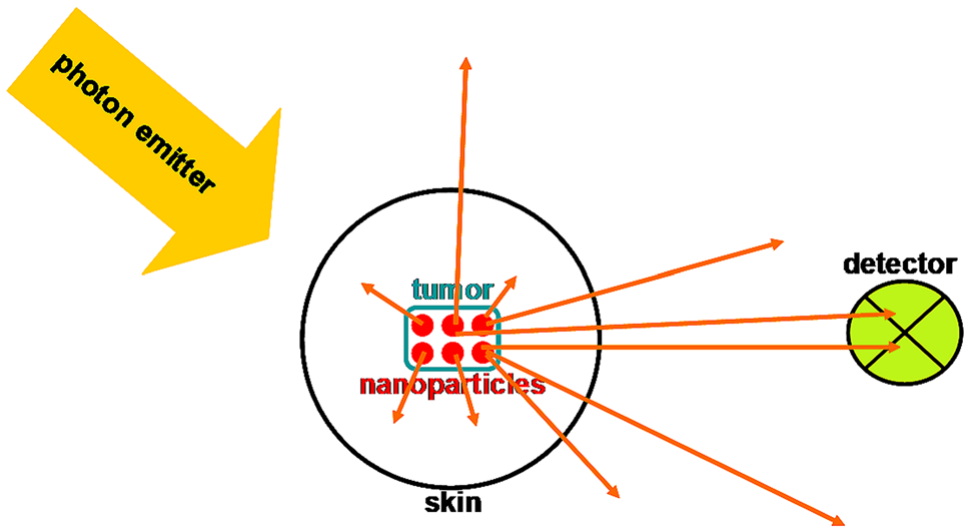
Essential elements in an optical arrangement for photoluminescent imaging of nanoparticles fixed in position inside a tumour located in a living human body. Large amounts of light enter the body through the skin, and during passage through the body fluids and organs the light is reduced in intensity. A small fraction of the light is absorbed by nanoparticles within the tumour, causing photoluminescence that emits light in all directions. A small fraction of the emitted light passes out through the skin into a detector, in which it triggers a count of the photons passing out of the skin. Each of these optical stages can be incorporated into an equation and used to model the imaging parameters and hence to specify the requisite design parameters of the nanoparticles

**Fig. 19:**
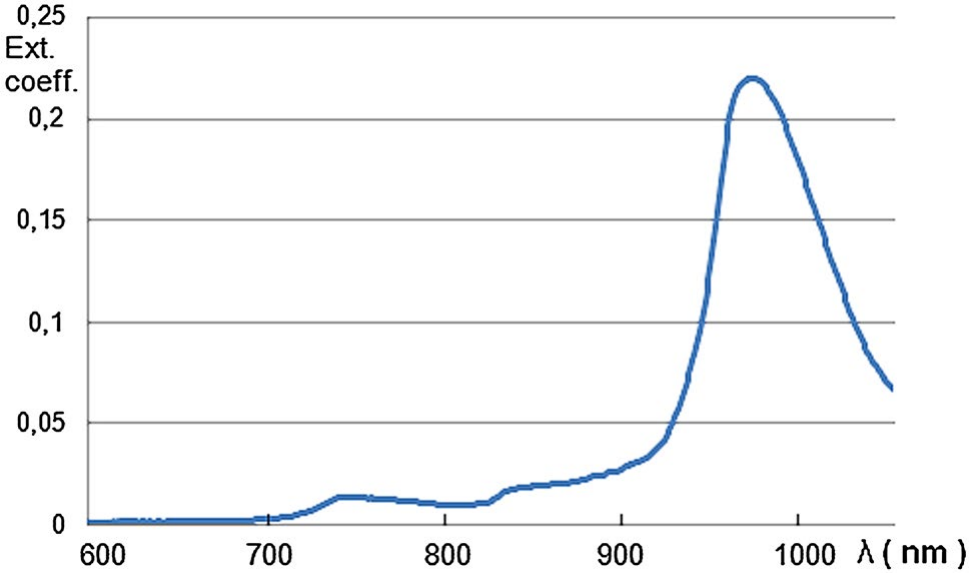
Extinction coefficient of water, graphed from lists prepared with spectral resolution = 1 nm and published in the internet (https://www.ucl.ac.uk/medphys/research/borl/data/matcher94_nir_water_37.txt). The “water window #1” is shown clearly between *λ* = 700 nm and *λ* = ~ 970 nm wavelength. Absorption by water in the body fluids and tissues is low in this spectral region. A second “water window” suitable for near-infrared imaging opens above *λ* = 1100 nm wavelength

**Fig. 20:**
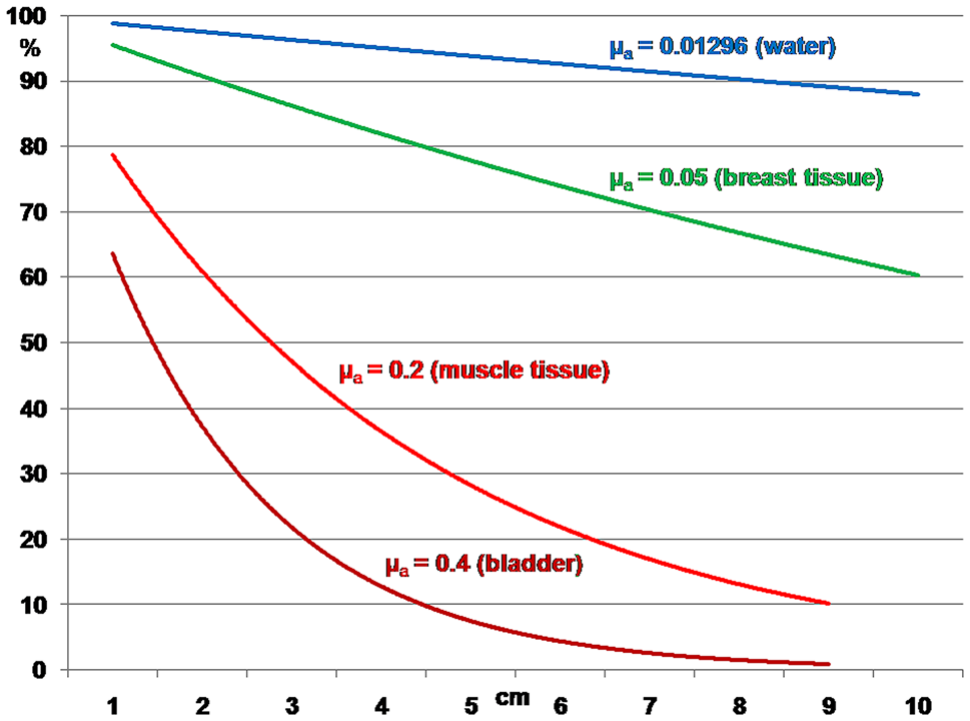
“Survival” fraction of light which enters an absorptive medium at left and travels through it towards the right, for a varying distance 1-10 cm. At distance = 0 cm all the four curves begin at 100% (not shown). Two examples are calculated for two general cases, where *µ*_a_ = 0.2/cm and *µ*_a_ = 0.4/cm; it happens that muscle and bladder tissues are members of these cases. One curve is calculated for breast tissue, which has *µ*_a_ = 0.05/cm at 750 nm (Bhatt et al. 2016). The curve for water is added for comparison

**Fig. 21:**
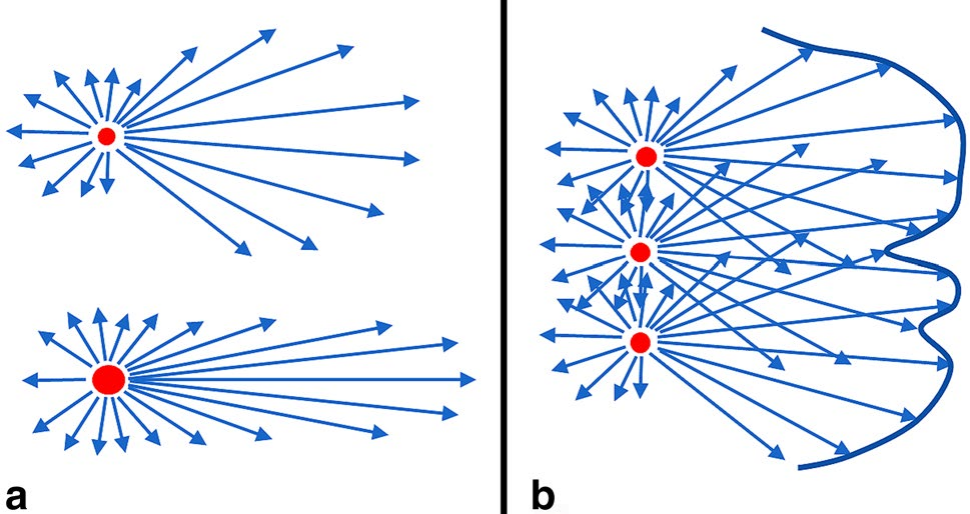
A: Mie scattering by small particles produces less forward scattering than scattering by larger particles. B: Mie scattering reduces spatial resolution and thus causes loss of fine feature detail

This Question compels us to focus on the context in which the nanoparticles will be used. In addition to the physicochemical characteristics of the nanoparticles it is necessary to measure their optical properties, the two most important ones being their optical absorption cross section and the efficiency with which they convert the absorbed light into emitted light. For many fluorophores and also many fluorescing nanoparticles, the absorption cross section has a value close to 10^−16^ cm^2^. The efficiency with which they convert the absorbed photons into emitted photons—their quantum yield—can be as high as 80% but is typically 20–30%. When the nanoparticles are fixed at, for example, a lesion site within the body, then the optical characteristics of the body tissues and fluids affect the light that can reach the nanoparticles and also the light that they emit towards a detector. Figure 18 shows how a chain of quantitative relationships can be constructed, beginning with a light source that illuminates the interior of the body through the skin, The skin absorbs some of the light to a degree which is quantified as the “attenuation factor” of skin, and the remaining light passes towards the nanoparticles through the internal tissues of the body. Each tissue attenuates the light at a rate that is specific for that tissue (Figs. 19, 20). The light that reaches the nanoparticles is absorbed at a rate determined by their optical absorption cross section (see above) and converted by the nanoparticles into heat and light (of a different wavelength). The luminescence from the nanoparticles is emitted in all directions and a fraction of it passes towards a detector. Figure 18 shows that the geometry of the optical arrangement is of great importance at this stage. The nanoparticles emit light in all directions, and thus will illuminate the entire surface of the body from within. Only the emitted light that reaches the detector can be registered, and this small solid angle of illumination represents only a tiny fraction of the light that the nanoparticles emit. The light passing from the nanoparticles to the detector will be absorbed and scattered (Fig. 21), again reducing the intensity of the light. Upon reaching the skin the light is once more subjected to a rather larger absorption by the skin before it exits the body and enters the detector—where part of it will be registered: the detector also has a detection efficiency which in present day equipment is 50–60% unless cooled to subzero temperatures. This double pathway through body tissues, beginning at an illumination source that will provide ~ 10^20^ photons per second and ending at the surface of the skin, where ~ 10^6^ photons per second will be registered, has been studied by numerous authors. Their publications provide guidance for researchers in Nanomedicine, quantifying the context for several types of literature that is available and we encounter repeatedly similar concepts and numbers, which taken together will define the power that must be designed into the nanoparticles.

For a beginning we consider briefly an early study that used a model that in many ways embodied aspects of our Fig. 18 (Ntziachristos et al. 2002). In that study about one quarter of a milliwatt (~ 10^18^ photons each second) was directed at the simulacrum and a maximum of ~ 10^5^ photons were registered at the detector (per mm^2^ each second). The losses of ~ 10^13^ photons occurred firstly during passage through the various simulated tissues: roughly by a factor of 10 for passage through 4 cm of lung or breast tissue, or through 3 cm of simulated denser breast tissue, or through 2 cm of muscle simulacrum or through 1.5 cm of brain simulacrum. The authors concluded that passage of NIR light (in their case, *λ* = 670 nm) through more than 10 cm of tissue is possible. The dye they used as target (instead of nanoparticles) had a typical absorption cross section of ~ 10^−16^ cm^2^ and conversion efficiency (quantum yield) of 0.28. The light emitted from the dye was of longer (Stokes-shifted) wavelength (*λ* = 694 nm) and a small fraction of that emitted light (~ 1/5000) passed towards the detectors. These would register only ~ 0.5 of the photons and in this case were cooled to reduce detector internal background. These values are all typical of a setup designed to view nanoparticles fixed in an internal organ (brain) and they are confirmed by numerous authors working in quite different fields. Neurosurgeons, psychiatrists and neuroscientists utilizing water window #1 found a penetration depth of 1.25 cm into the human skull, imaging cortical vasculature and blood flows (León-Carrión and León-Domínguez 2012), and others studying the brain state found penetration depths close to 3 cm (Okada and Delpy 2003).

**Fig. 22:**
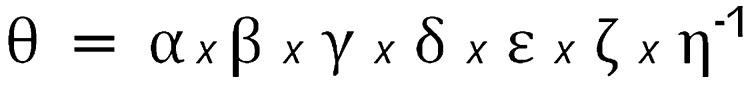
Quantitative relationships in near-infrared nanoparticle imaging. The terms of the equation refer to stages in the optical imaging pathway, as shown in Fig. 18. Varying the values of the terms in the equation can be used to model imaging and thus help to identify close-to-optimal values for designing an appropriate nanoparticle

In Table 3, we show attenuation lengths for some internal organs, referencing their sources. In Fig. 22, we formalize the chain of factors that are essential to consider in designing nanoparticles for NIR viewing “deep” within the body.

**Table 3:** Optical coefficients for a selection of tissues

Tissue	*λ* (nm)	*µ*_a_ (cm^−1^)
Water	600	0.01
Fat	630	0.52
Lung	780	0.1
Breast	660, 780	0.037–0.05
Bone	650	0.09–0.14
Brain	780	0.15
Muscle (human)	661, 780	0.15–1.08
Skin	630	1.3
Bladder	630	1.4
Liver	630	3.2
Hemoglobin	665	4.87
Oxyhaemoglobin	665	1.30
Muscle (rat)	980	1.8

For further values, see Bhatt et al. (2016), León-Carrión and León-Domínguez (2012), Ntziachristos et al. (2002) and Jacques (2013)

*Wavelength-dependent absorption* The attenuation of a light ray passing through a biological tissue is due to the absorption of photons by molecules in the tissue, and by scattering of photons from particles present in the tissue. We consider absorption first. Biological tissues are rich in water, which has a low coefficient of absorption, approximately 0.001, in the spectral range from *λ* = 600 nm–680 nm, where the value starts to rise, reaching 0.01296 at *λ* = 750 nm (Fig. 19).

As a result of this low absorption coefficient light penetrates water without losing much intensity. Figure 19 shows “water window #1”, the range of wavelengths at which water has low absorption of light; above *λ* = 950 nm the optical absorption coefficient reaches values above 0.2 and the water window #1 closes. Tissues absorb light more strongly if blood or pigment molecules are present, and in these cases light penetrate less deeply into the tissue (Fig. 20).

*Particle size-dependent scattering* Particles of diameter 0.1–1.0 times the wavelength of light scatter the light, deflecting photons away from the optical axis. As a photon encounters further particles it may be deflected many times. The NIR wavelengths near *λ* = 1000 nm = 1 µm are, therefore, scattered by cell organelles such as cell nuclei. The type of scattering at NIR wavelengths is denoted Mie scattering, after Gustav Mie who developed the theory as an analytical solution of Maxwell’s equations. Mie scattering has a tendency to scatter forwards (Fig. 21). This scattering does not reduce penetration of a tissue to a high degree, but it does reduce light intensity and cause loss of spatial resolution (Fig. 21).

The equation shown in Fig. 22 does not assume that the localized population of nanoparticles will be imaged. It suffices that their presence simply be detected: the detector can be aimed (like an ultrasound scanning head) through the skin in different directions and the occurrence of a localized signal be recorded. For many clinical purposes more is not needed: the presence of a lesion is flagged and it indicates the need for further clinical investigation.

It is evident that the above equation can only deliver approximations at present. However, as written down it does demonstrate that all the necessary parameters can be measured and that a resulting requisite density of nanoparticles can be calculated. This in turn provides specifications to guide the development of the nanoparticles. As shown above, “quantum nanoparticles” already exist that require only limited further developmental work to satisfy the parameters assumed in our finger exercise. Rational design and development of the specified nanomaterials can be carried out supported by reference to the well-funded and intensive industrial and academic research programs presently being undertaken in optics, electrons, photovoltaics, and related disciplines.

Figure 18 shows that light emitted from a light source passes through the skin into the body tissues, then through the body towards the nanoparticles and then through the body again towards the detector, and finally leaves through the skin. The passage through the skin and the body tissues, which occurs twice and at two different wavelengths, cannot be modified by the operators of the illumination and detector systems, and it cannot be altered by the creator of the nanoparticles. We consider this double trip through the body next. Light is absorbed and scattered as it passes through fluids. Each of these is important in reducing light intensity and scattering also rapidly distorts any image that has been formed. We discuss absorption first and scattering thereafter.

In the ideal case all the light passing into the body would be absorbed solely in the nanoparticles fixed in place within the tumour (see Fig. 18). There the photons would be re-emitted as fluorescence or they would be converted into heat. However, the fluids and tissues between the light source and the nanoparticles will absorb some of the photons. We will assume that the artefacts arising from contact-free imaging will be avoided; these included reflected light from the skin and off-axis scattered extraneous light. We, therefore, assume the light source has contact with the skin. The fluids through which the light must pass can be approximated as saline water with an ionic composition resembling seawater. Here Nanotechnology can benefit from the careful studies that have been carried out on seawater for oceanographic purposes. Saline water does not itself have high absorption of light in the “NIR window” of the red/infrared spectrum. In seawater there are plankton and organic residues that absorb light, these are lacking in body fluids, but the blood vessels in the tissues contain other significant absorbers of light, for example, hemoglobin. The absorption coefficient of body tissues varies from one fluid and organ to another and is, furthermore, wavelength-dependent. Numerous authors have published relevant data for essentially all the organs of humans and also a range of animals. The relevant data for this review are measured within the “NIR water windows”, though a majority of authors have favoured measurements made close to *λ* = 600 nm. Figure 20 shows how critical the absorption coefficient is, since with each further centimeter that the light travels it loses in intensity an exponential amount based on the absorption coefficient. Table 3 shows that several tissues in the body do indeed reach high levels of absorption.

Mié scattering (Fig. 21) is the dominant factor causing loss of light intensity in the body at “Water Window #1” NIR wavelengths, from *λ* = ~ 0.1 µm to *λ* = ~10 µm. Mié scattering occurs when the size of the scattering particles ranges from 1/10^th^ of the wavelength to ~ 1× the wavelength. Scattering depends on the number of scattering particles and may be directed at any angle to the optical axis—including both directly forward and directly backward; Mié scattering tends to include a large forward scattering. A scattering event leads to confusion of the initial feature information including broadened feature sizes: this results in a halo around the central axis, as observed for car headlights in fog, or for the sun in fog. Passage through turbid media is deflected in all directions from the optical axis, the effect being dependent on the relationship between the wavelength of the light and the size of the particles causing scattering. Thus the presence of living cells containing organelles with sizes close to 1 µm (cell nuclei, mitochondria, etc.) deflects light with wavelengths close to 1 µm = 1000 nm, that is: the red and near infrared. Light propagation under multiple scattering conditions, which is typically the case for tissues having thickness > 1 cm, has been modeled accurately using the Monte Carlo simulation, which is considered the gold standard (Bhatt et al. 2016; Hiraoka et al. 1993). The scattering results in spread in the image and loss of feature contrast; the image is degraded. Numerous publications present variations on the mathematical models of scattering, but other authors report experimental measurements of various organs. In the NIR “Water Window #2” scattering is less dominant and the attenuation of light intensity occurs mainly due to absorption, and image features are not degraded (Bhatt et al. 2016).

The principle is shown in Fig. 18. We restrict this review to planar imaging as shown in the figure, for reasons explained below.

Imaging success is represented in Fig. 22 in terms of a signal *θ* that is at least 3× stronger than the total noise (*η*) in the imaging system. We follow the light along the pathway to the nanoparticle deep in the tissue, and then follow the emitted light back to a detector. The losses along this pathway begin with *α*, which quantitates the loss of light intensity occurring between the light source and the nanoparticle fixed deep within the organism; this value is summed from the attenuation due to the skin and to any tissue or fluid that lies between the light source and the nanoparticle. The term *β* quantifies the absorption cross section of a single nanoparticle. *γ* quantifies the light emitted as photoluminescence from the nanoparticle (“Quantum Yield”). *δ* quantifies the amount of photoluminescent light that reaches the detector, accounting for the attenuation losses as the light passes through the tissue between the nanoparticle and the detector. *ε* reduces the amount of light entering the detector by taking into account the (usually small) solid angle subtended by that detector as seen from the emitting nanoparticle. Finally, *ζ* reduces the number of photons counted by the detector due to the inefficiency of detection. The equation shown in Fig. 22 assumes that both light source and detector are in contact with the skin, because it contains no terms accounting for reflection and off-axis scattering (Funane 2012). Table 4 shows a finger exercise that demonstrates how this equation can be used in rational design of a photoluminescent nanoparticle.

**Table 4:** Calculation of light-path parameters for infrared imaging

Pathway description	Factor	Value
Light source	Input to produce luminescence	300 mW = ~ 1.6 × 10^18^ photons
*α*	Attenuation between light source and NP	0.1 × at 4 cm
*β*	Absorption cross section of one single NP	1 × 10^−16^ cm^2^
*γ*	Quantum yield of NP	0.3
*δ*	Attenuation of NP-emitted light at distance to detector	0.1 × at 4 cm
*ε*	Solid angle of detector at 4 cm seen from the NP	~1/5000 = 2 × 10^−4^ at 4 cm
*ζ*	Detection efficiency of detector	0.5
*η*	Total noise in the system	1 × 10^−6^ counts/mm^2^.second
*α*_*x*_ *β*_*x*_ *γ*_*x*_ *δ*_*x*_ *ε*_*x*_ *ζ*_*x*_ *η* ^−1^	$1.6\, \times \,10^{18}_{x} 0.1_{x} 1\, \times \,10^{ - 16}_{x} 0.3_{x} 0.1_{x} 0.2\, \times \,10^{ - 4}_{x} 0.5_{x} 1\, \times \,10^{ - 6}$	4.8 × 10^−11^

At this S/N ratio (4.8 × 10^−11^) the signal is not detectable

To obtain an adequate signal (S:N > 3) the number of NPs could be increased from 1 to [3 × 4.8 × 10^11^ =] 1.44 × 10^12^ NPs fixed at that location in the tissue. Alternative design options would be to increase the quantum yield of the nanoparticles by doping (see above), or to increase the detection efficiency of the detector by cooling (see Ntziachristos et al. 2002), or to employ a wider aperture detector, or to increase the power of the light input. The researcher might opt to combine several of these possibilities. The power of this approach lies in the integrating the nanoparticle characteristics from the beginning of the design stage into the context in which they are planned to be used. There is a major limitation affecting this calculation: if the number of NPs resident in the tissue is to be increased to 1.44 × 10^12^ then the tissue must contain this number of binding sites for the NPs. This number must be available, either from earlier publications or from measurement of it during the nanoparticle design stage. Note also that Fig. 22 does not specify a wavelength for the input photons, nor for the specifications of the detector at a given wavelength. These wavelengths are not directly relevant to the calculation, but they are nonetheless critical factors in NIR molecular imaging, and will be discussed next.

We, therefore, consider that the early developmental phase of Nanotechnology in Medicine is essentially complete. It was the search for adequate materials at the nanoscale. The search ends with the recognition that for most nanomedical purposes Quantum Nanoparticles can be designed. This developmental work can assume that the materials used will be of limited toxicity, and the nanoparticles best constructed from materials such as carbon, which is why this review has focussed on the allotropes of carbon. The particles may require doping and they should be doped if possible with non-toxic dopants; but it should at the same time be recognized that the requisite quantities of dopants reflect amounts that are several orders of magnitude less than the amounts of nanoparticles required and that the amounts of nanoparticles required can also be extremely small. In undertaking the necessary developmental work the researchers in quantum-based Nanomedicine should look over the interdisciplinary fences to keep up-to-date with the developments being made at rapid pace in the neighboring disciplines of optics, electronics and materials research.

As a research discipline, Nanomedicine can now move forward to confront the Grand Challenge posed by the highly effective biological barriers that are present many thousands of millions of times in the human body. We note a recent report on mechanically disruptive nanoparticles acting as scissors cutting at megahertz rates and able to shear through cell membrane bilayers and which can be targeted to specific cells (García-López et al. 2017).

## Targeting in nanomedicine

### Targeting—the two sides of a coin

Nanoparticle targeting still counts as the major challenge to be solved in nanomedicine (Lammers et al. 2016). Targeting of nanoparticles describes the lesion-directed imaging and/or delivery of drug molecules via a nanoparticle cargo in vivo. This requires understanding of in vivo biology and of the interactions between the nanoparticles and the biological environment they encounter after application (Albanese et al. 2014); i.e., understanding cellular uptake mechanisms, blood–tissue barriers, inter-particle interactions in vivo, identifying and using organ-specific receptors for receptor mediated uptake and the making of stable nanoparticle units (Lane et al. 2015). A bio-functionalised nanoparticle should be seen as one single and stable working unit. This unit is built up of a nanoparticulate matrix, e.g., liposome, dendrimer, gold, protein, quantum dot; of imaging moieties and/or drug molecules; and possibly of targeting ligands. This unit has to survive in vivo conditions, the stability of this unit is crucial to ensure long enough circulation until it reaches its target lesion.

During evolution, the mammalian body established variously structured blood–tissue and tissue–tissue barriers, which present obstacles for nanoparticles whether these are applied into the bloodstream or topically. Only one organ, the liver, has no barriers at all. All other organs can be classified as having either low, high or extremely high barriers, which exhibit correspondingly differentiated states of structural and physiological organization. These barriers are of crucial importance in the targeting of nanoparticles (Debbage and Thurner 2010). They comprise a range of barrier mechanisms which are present in specific tissues and are constructed to provide optimal protection of the “private” space within the tissue parenchyma. Some organs maintain more than one such space, for example, the kidney. The barriers vary in their structures and in their physiology, and can be considered as “high”, or “low”. The “high” barriers are found, e.g., in the Central Nervous System and the testis, whereas “normal” barriers are represented, e.g., in exo- and endocrine glands and the gastro-intestinal tract, and “low” barriers are present, e.g., in the lymph nodes and the spleen. These natural barriers necessitate careful consideration of the route of application. Failure to contemplate the barriers adequately has been extremely expensive for the pharmaceutical industry (Ledford, 2010). If nanoparticles are applied orally or parenterally they will face at least five different barriers on their way to their target cells,

1. from application site to target epithelium/endothelium,
2. from target epithelium/endothelium to lesion interstitium,
3. from lesion interstitium to neighbourhood of target receptor,
4. from neighbourhood to close vicinity of target receptor,
5. receptor mediated entry into the target cell.

A nanoparticle equipped with the multiple targeting groups necessary to access its targets in, e.g., a gland such as the prostate or the breast, would offer considerable difficulties in translational development and would meet with high regulatory burdens on the path to clinical licensing. Thus, although the possibility of such targeting has been demonstrated and the targeting efficiency can be as high as 89% (McIntosh et al. 2002), nearly a 100-fold increase in contrast to common small drug molecules which possess an efficiency of roughly 0.01% (Dvorak et al. 1991; Ferrari 2005), this demonstration now lies several years in the past and there has been no sign that it is being followed up actively by the industry. In contrast, topical application generally requires the nanoparticle to be equipped with only a single specific targeting group and is, therefore, much easier to develop at the translational research stage.

*Tumour biology* The large majority of nanoparticles in research is directed to target solid tumours; their microenvironment is characterized by a nearly insurmountable peri-vascular lining of pericytes, smooth muscle cells and fibroblasts, and a high density of malignant cells within the tumour itself. The constitution of the endothelial lining surrounding the tumour site regulates the circulation of blood into the tumour interstitium. A large porous structure of the endothelium increases the permeability of blood into the tumour thereby increasing the interstitial fluid pressure (IFP) and decreasing pressure differences to the microvasculature. In addition, the presence of fibrosis and the contraction of interstitial matrix due to tumour growth influence the IFP (Heldin et al. 2004). The IFP is common to all solid tumours and comprises a significant barrier to the entry of all nanoparticles into the tumour tissue, independent if targeted or not. Nanoparticles possessing targeting ligands (see below) encounter further obstacles. Targeting ligands use the expression of respective antigens to attach onto the surface of malignant cells. However, the expression of such antigens differs from individual to individual, and from early stage to late-stage tumours. Some tumours do not express tumour-specific antigens at all but antigens that are also present on healthy cells. In addition, targeted nanoparticles are likely to bind to the first receptors they encounter. If these are receptors situated on the tumour surface they will block the nanoparticles from deeper penetration into the tumour. This binding-site barrier together with the difficulties in identifying the correct targeting ligands are reasons why there are only a few targeted nanoparticles in clinical trials, and none in clinical use so far.

*Passive targeting* Confronted with the daunting scientific and regulatory difficulties involved in targeting blood-borne nanoparticles to intracellular targets hidden by a series of active and passive barriers, industrial research has opted for the simplest form of “targeting”, which allows limited access to tissues, because the physiological and morphological barriers have been partially disrupted by pathological conditions in the tissue. This much easier access for nanoparticles to their target sites uses the so-called *enhanced permeability and retention effect* (EPR effect). However, EPR does not represent any form of active, i.e., receptor mediated, targeting; it bases only on the hyperpermeability of blood vessels exposed to pathological conditions as, e.g., in sites of inflammation or late-stage tumours. EPR permits “targeting efficiency” to reach approximately 1%, (Wilhelm et al. 2016), this indeed is a 100-fold increase compared to common small-molecule drugs which possess an efficiency of ~ 0.01% (Bae and Park 2011). However, large volumes within tumours may lack blood vessels, and cannot be reached by EPR-“targeted” nanoparticles, the fact that severe side effects are associated with most anti-tumour therapeutics means that nanoparticles “targeted” by EPR typically achieve an anti-tumour treatment far below the therapeutic maximum needed to eliminate all tumour cells, and these agents are limited in use, because they still exhibit a “therapeutic window”. The present state of the art in breast cancer treatment, Abraxane^®^, exploits leaky blood vessels, thereby restricting therapy to late-stage tumours, reducing the overall survival chances drastically. It is a significant drawback for EPR-“targeting” that some organs physiologically possess hyperpermeable microvessels, e.g., some segments of the kidney tubules, the bone marrow, the thyroid gland, the pancreas, the spleen, and most notably the liver (Debbage and Thurner 2010) leading to liver uptake of the majority of passively targeted nanoparticles, followed by their destruction. These organs represent sites for nanoparticle accumulation which could lead to toxic side effects. The deeper consideration is that, by its very nature, EPR-“targeting” can only deliver nanoparticles to sites which already show pathologically disturbed tissues such as in advanced cancer or inflammation: it cannot deliver to targets which urgently require therapy but are still hidden behind intact blood vessels, e.g., early stage cancers or metastasis. In addition, the EPR effect, which was first described by Maeda in 1986 (Matsumura and Maeda 1986), is far more pronounced in rodent cancer models than in human tumours. The reason for this is simple, rodent tumours grow much faster; therefore, the vessel structure inside the tumour is much less differentiated than in a slowly growing human tumour. This further means that nanoparticles which show significant tumour uptake in animal models are very likely to fail in the human situation (Lammers et al. 2012).

In addition to the blood–tissue and tissue–tissue barriers described above, special consideration has to be taken into account of the tumour microenvironment. Nanoparticles which manage to extravasate the circulation into the tumour environment will encounter different layers of biological tissue and different physical constraints. Some parts of the tumour might be accessible rather easily, other parts will be covered with some layers of pericytes, smooth muscle cells and/or fibroblasts; the interstitial fluid pressure (IFP) is a common feature of solid tumours additionally hindering nanoparticles to access the cells (Lammers et al. 2012).

*Active targeting* The active targeting strategy uses targeting ligands attached to the surface of nanoparticles to enable receptor mediated uptake into lesion cells (Jahan et al. 2017). This strategy should increase the concentration of nanoparticles inside the lesion cells thereby increasing the therapeutic efficiency and reducing potential adverse side effects. However, reality looks less promising. The majority of nanoparticles in clinical trials and clinical use are passively targeted systems, no actively targeted nanoparticle has yet reached the clinical market (Shi et al. 2017; Ventola 2017). Active targeting needs deeper considerations on the biology of the patient compared to passive targeting. Several questions must be addressed simultaneously: which cells to target? Which ligand to use? How to attach the ligand onto the nanoparticle?

The route of nanoparticle application, i.e., parenteral or topical, together with the selected illness determines the number of biological barriers to be overcome, thereby determining the type of cells which should be addressed. A nanoparticle targeting cancerous tissue and applied intravascularly, therefore, would need to attach to endothelial cells to extravasate from the circulation as well as to the epithelial cells of the cancer to be internalized. In contrast, locally applied nanoparticles, e.g., in the gastrointestinal tract addressing inflammatory lesions or intravascularly addressing atherosclerotic plaques, would require only one type of targeting ligand, i.e., to target epithelial or immune cells (Debbage and Thurner 2010). It follows that the route of application and the selected illness also determine the types of targeting ligand to be attached onto the nanoparticle surface; each type of cell needs its respective targeting ligand.

Different targeting ligands are available, e.g., transferrin, thrombin-specific cleavable peptide (TAP), aptamers, antibodies (Jahan et al. 2017).

The chemistry of how to attach these ligands onto the nanoparticle is crucial in achieving site-directed targeting. A common but not very successful strategy in nanotechnology is to simply adsorb the ligand onto the nanoparticle surface; however, this physical connection can easily be disrupted in vivo due to changes of charge and pH, leading to the inability of the nanoparticle to bind to the respective receptor. Sophisticated chemistry is required to bind the ligand onto the nanoparticle surface covalently (Jahan et al. 2017) (i.e., stable to survive in vivo conditions), and thereby allowing receptor recognition (i.e., no disturbance of the antigen-binding sites). Chemical procedures have been introduced which bind the ligand covalently, the most common one is 1-ethyl-3-(3-dimethylaminopropyl) carbodiimide hydrochloride (CDI) (Jahan et al. 2017). However, unspecific attachment of the targeting ligand is pre-programmed as CDI attaches to any primary amino groups; in addition, CDI fixes the targeting ligand onto the nanoparticle as no spacer arm is present to allow the ligand to move into its correct orientation. The authors own experience has shown a difference in the binding efficiency of CDI-targeted nanoparticles and of nanoparticles targeted via a sophisticated linker (unpublished data).

Comparing the architecture of the viruses, their sizes are between 22 nm (parvo-virus B 19) and 300 nm diameter (pox-virus) (Hahn et al. 2001), they possess a certain number and arrangement of their targeting spikes on the surface, and their flexible targeting spikes protrude between 5 and 20 nm out of their surfaces (Plückthun 2010). Viruses will be discussed in more detail below. Taking this into account, the size of the nanoparticle determines the number, the density and the length of the targeting ligand needed for successful receptor interaction. The targeting ligands on nanoparticles have to be covalently attached via a flexible spacer arm that allows rotation of the ligand according to the conformational requirements of its receptor. This spacer arm should also bind the ligand in the correct orientation not disturbing the antigen-binding sites and not disturbing the natural charge of the ligand which is essential for interacting with its receptor. Receptor recognition depends on the correct orientation and antigen-binding capability of the ligand. Effective targeting can only be realized with a proper spacer arm (Klement et al. 2015; Chen et al. 2013).

In addition, the structural conditions for receptor interaction need chemical spacers connecting the antibodies to the nanoparticles covalently leaving the antigen-binding sites intact. Some research groups have already successfully employed natural spacer molecules, e.g., protein G (Lim et al. 2009) or biotin (Pramanik et al. 2016). There exist some databases with natural and artificial linkers comprising different functionalities and different molecular weights (http://synlinker.syncti.org/, http://www.ibi.vu.nl/programs/linkerdbwww/). The spacer arm which attaches the targeting ligand to the nanoparticle is an important part of the nanoparticulate unit and a crucial feature in nanoparticle design, including the correct nanoparticle matrix and the correct specificity of the targeting ligand. Nanomedicine still holds the promise to site-directed treatment of diseases minimizing adverse side effects, this being the key for precision medicine; however, the active targeting challenge has to be solved.

*Viruses* As noted above, viruses are natural nanoparticles that target actively to the cells in which they will take over the synthetic apparatus and replicate themselves. They are adapted to function in hosts which over long periods have been developing barriers to entrance and thus, in the unremitting selection processes that run rapidly during their short replication cycles, viruses have adopted various strategies to bind specifically to, and enter, their target cells which hide behind barriers, for example, behind thick mucus barriers in the respiratory tract. The influenza A virus, 84–170 nm in diameter (Harris et al. 2006) targets the ciliated epithelia of the oronasal respiratory space in birds and mammals (Thongratsakul et al. 2010). Later we will consider the HIV-1 virus, 110 nm in diameter (Zhu et al. 2006) and thus of comparable size to the influenza A virion. Both these viruses attach to their target cells by binding specifically to receptors present on the surfaces of the target cells. For influenza A virions, this binding process has been studied at high resolution in both time and space by cryoelectronmicroscopy, atomic force microscopy and molecular dynamics simulations. The initial weak binding by one or two specific sites on the viral spikes is followed within nanoseconds to milliseconds by either disruption of the linkage via stormy thermal fluctuations of the surroundings, or it is stabilized by the binding of further specific sites to the surface receptors (Sieben et al. 2012). Influenza A virions do not need to bind efficiently to their target cells, because, once inside the cell, they will replicate with high efficiency and produce many thousands of successor virions. It is decisive for them that they bind rapidly before the mucus flow washes them away, and they maximize the chance that each physical contact with the target cell will lead to binding and entry. The Influenza A virus represents the extreme of one strategy: it bears the maximum possible density of spikes on its surface, ensuring that the initial binding to the target will be followed rapidly by subsequent binding of neighboring spikes, thus ensuring the most efficient binding and entry into the target cell. This strategy is both expensive (in terms of spike numbers that must be produced) and it maximizes the likelihood of immune recognition, but it also provides a rapidity of binding minimizing the danger that the flowing stream of mucus in the respiratory tract will wash it away before it can bind to the target cell.

The HIV virus represents the extreme form of quite a different strategy: it produces the minimal number possible of viral spikes necessary to enable their binding to the target (Klein and Bjorkman 2010). It resides less than 6 h in the host’s tissues and bloodstream (Wei et al. 2014) and targets CD4 target molecules on the surfaces of dendritic cells and white blood cells (CD4^+^ T cells). Its binding efficiency is extremely low and only a small minority of physical contacts with the target cell will lead to binding and entry into the cell. The minimized number of antigenic spikes does, however, confer the advantage that there is a low likelihood that recognition by the host’s immune system will occur.

There are, therefore, two quite different targeting strategies that are demonstrated in the natural world and the design of artificial nanoparticles will involve a design choice in the direction of one of them. Since the targeting antibodies are usually the most expensive items constituting the target nanoparticle, the HIV strategy of minimizing the number of binding sites per nanoparticle offers the cheapest way to construct the particle. We designed and prepared nanoparticles based on this strategy and used them to obtain strong specific binding and imaging (in preparation for publication). We recognize, however, that there are circumstances in which it may be necessary to adopt the strategy of the Influenza A viruses.

### How to improve targeting—what the literature suggests

Different ideas have been employed to improve the targeting of nanoparticles, however, most of them use additional chemical or physical stimuli which by themselves could cause adverse reactions or even support the development of cancerous mutations inside the cells they strike, e.g., the application of heat or light or the use of radiotherapy.

*Stimuli responsive targeting* External stimuli such as heat, light, ultrasound or magnetic fields are applied to change the microstructure of the tissue, connections between cells are loosened, vascular pores increase slightly in their diameter. Application of such stimuli requires to know the exact location of the tumour and metastases in advance. In contrast, internal stimuli such as pH differences, presence of certain enzymes, temperature differences or redox gradients use biological differences between diseased and healthy tissues (Tong and Langer 2015). However, these differences can differ from person to person, and from organ to organ, rendering their reliability rather low.

*Penetration* Pharmacological means such as collagenase or relaxin are applied to tumours prior to chemotherapy to render tumourous tissues more susceptible to anticancer drugs (Danhier 2016). Studies have shown a 2–3-fold increase in intra-tumoural delivery of antibodies and macro-molecules. Another approach that has been tested is the combination with radiotherapy. Radiotherapy induces the expression of VEGF and FGF, endogenous molecules responsible among other things for the growth of blood vessels and angiogenesis, respectively. By their functions, VEGF and FGF, therefore, can lower the interstitial fluid pressure helping nanoparticles to extravasate into the tumour. However, cancerous tissues also use VEGF for growing metastasis. Application of radiotherapy has to be seen very critically: a shot towards the tumour can easily be fired back.

*Multistage delivery systems* Nanoparticles have been designed possessing sizes around 100 nm diameter; these nanoparticles show prolonged circulation in vivo. Tumour proteases such as MMPs degrade these nanoparticles after extravasation into 10 nm sized particles which are able to penetrate deep into the tumour (Stylianopoulos et al. 2012).

### How to improve targeting—what we suggest

Select carefully between the Influenza A and the HIV-1 targeting strategies.

Ensure that the targeting region of the antibody molecule is situated several nanometers from the particle surface and faces away from the particle body;

Ensure that the targeting region of the antibody can rotate slightly;

Attach the targeting group covalently to the particle body (Fig. 2).

## Translation to the clinical market

### The outline of this story

In theory, nanoparticles clearly have advantages over common small drug molecules:

- they are too big to cross intact (i.e., healthy) blood–tissue barriers randomly,
- they enable lesion-site-specific imaging improving diagnosis,
- they enable lesion-site-specific drug release reducing adverse effects and overall drug dosages.

**Fig. 23:**
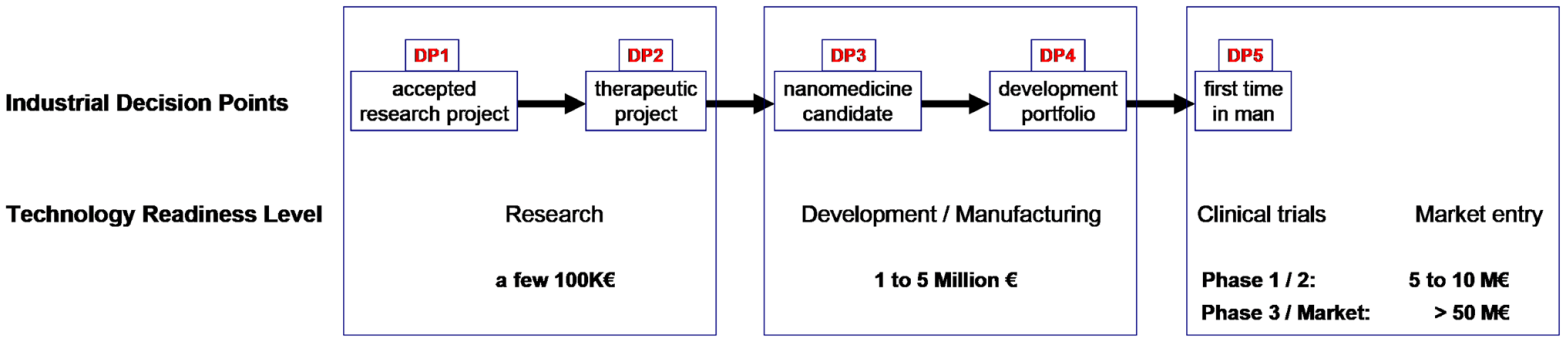
Research and development (R and D) pathway from laboratory to clinic. The steeply rising costs associated with each step in RandD are shown, and the hiatus between academic research and developmental financing is clearly visible. Adapted from Eaton et al. 2015

However, only about 2% of all the nanoparticles discussed in the published literature have entered clinical trials (Wicki et al. 2015), and only a fraction of those have actually entered clinical use (Shi et al. 2017; Ventola 2017; Bobo et al. 2016). Most of the nanoparticles fail in the last stages of their clinical trials (Fig. 23), because they do not prove to be better than the already established small drug molecules or cause adverse immune reactions. In vivo and tumour biology as well as nanoparticle chemistry determine the effectiveness of nanoparticle targeting.

**Fig. 24:**
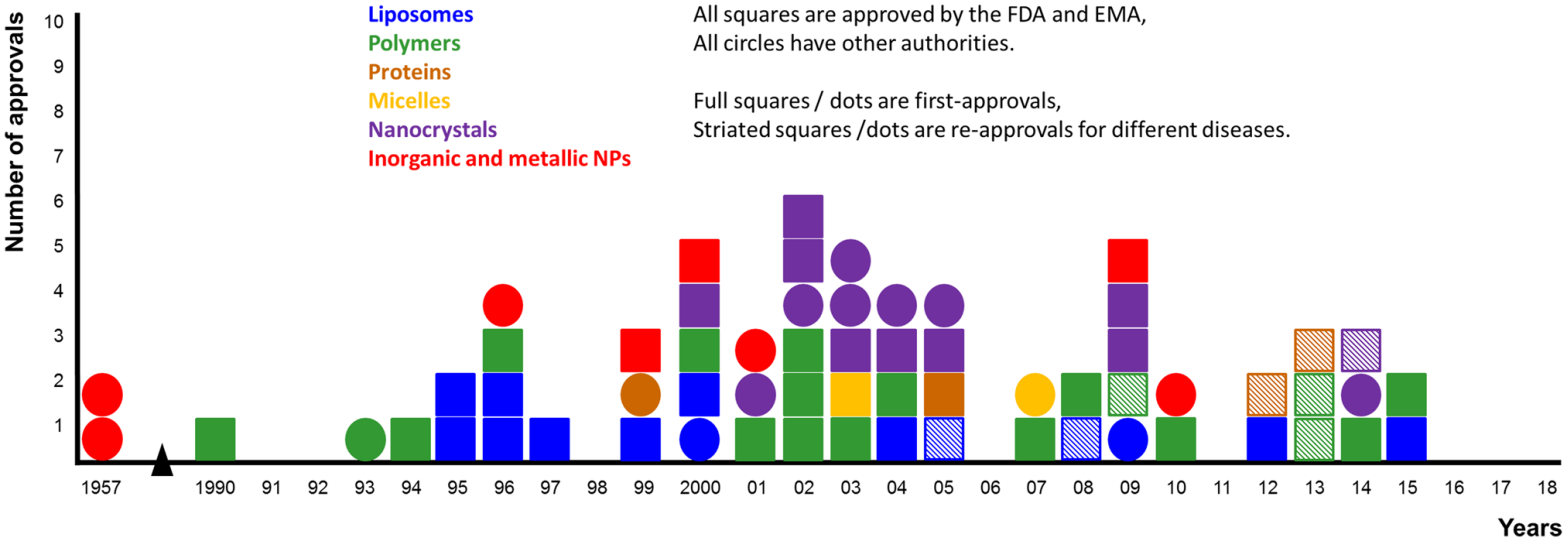
Approvals of different nanoparticle types during the last 60 years; note that some “approvals” were for new indications of previously licensed nanoparticles

**Fig. 25:**
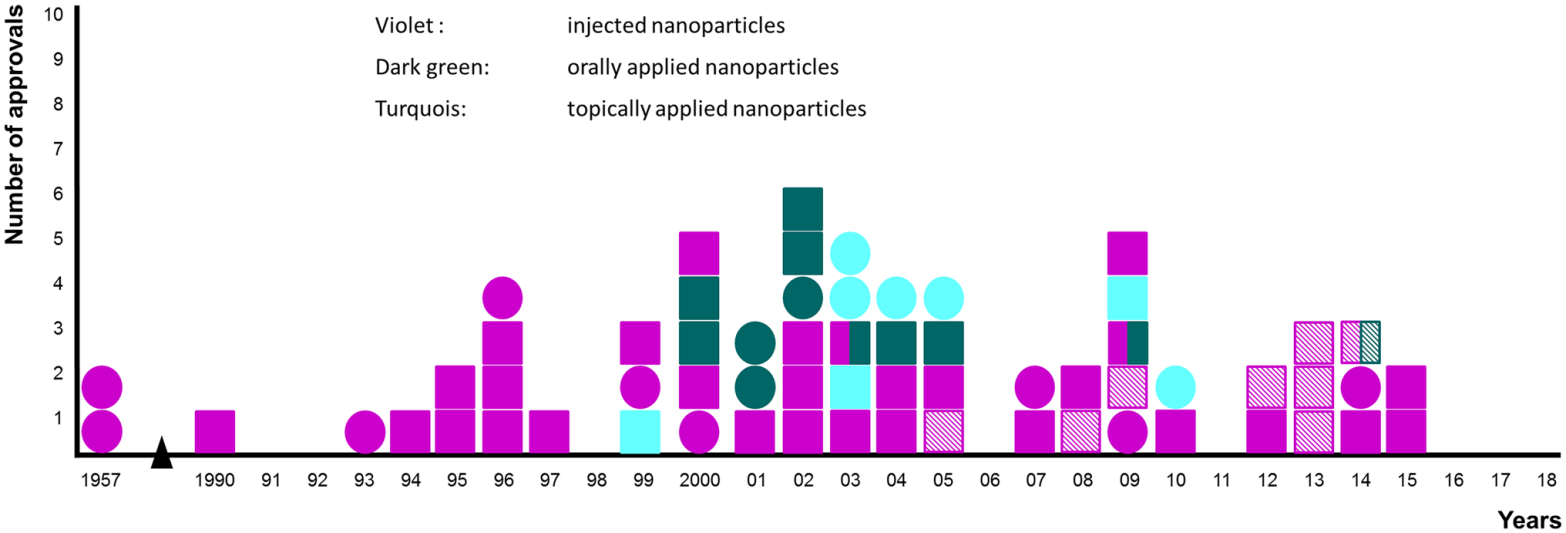
Approvals of the same nanoparticles, as shown in Fig. 24, but here categorized by the approved portals of clinical application

Since the beginnings of nanotechnology in the 1970s many different types of nanoparticles have been developed for clinical use. Organic nanoparticles are made of either natural materials, e.g., proteins, sugars, lipids, or viral components, or are fabricated via chemical synthesis, e.g., synthetic polymers. Such nanoparticles often are composited of natural as well as of synthetic materials, e.g., protein particles covered with polyethylene glycols for stealth characteristics. Some have already entered clinical use, e.g., as liposomes AmBisome^®^, Doxil^®^/Caelyx^®^, Myocet^®^, as polymers Pegasys^®^, Neulasta^®^, Renagel^®^, as proteins Abraxane^®^ (Zhang et al. 2008; Bobo et al. 2016) (Figs. 24, 25).

There has been no explosive blossoming up of clinical applications. The strongest period of granted licenses is behind us (2000-2005). Why has the clinical aspect of Nanomedicine stalled? It is not due to a lack of novel NP formulations. There has been no slackening in the numbers of novel NPs being reported. One explanation is internal and requires action by ourselves, the academic light-bringing dwarves (Fig. 1). Our light at present is dark. A second problem is external and requires action from outside the immediate research circle. We will discuss each of these two challenges in turn.

The first is due to a blind spot in ourselves, the researchers. We authors remember one of Feynman’s judgements after his visit to a major research center in a different country, that the people there were occupied in carrying out several types of excellent research, but that “the whole institute had been avoiding confrontation with the major issue in that research field for many years now”. We believe this is happening in nanomedicine. There is a fascination with the exotic, the sophisticated and the expensive in Nanomedicine, and we await a Henry Ford to ask: “Is that really necessary?” and to tell us: “You can have any color you like, provided you like black”. In this review there has been mention of at least one area in which emphasis on sophistication is hampering development of necessary but simple approaches; the KISS principle recently elaborated in USA politics has not taken root in Nanomedicine. This is compounded by the fact that “bread-and-butter” research to investigate the “Boyg” (in Ibsen’s play) is not easy to get funded. We have observed that projects promising the sky do much better with the reviewers of the funding bodies than projects aiming to further develop yesterday’s or this morning’s innovative sky.

The field as a whole remains focused on creating novel NP chemistries. However, the challenge has moved on. The major and wholly unsolved challenge facing Nanomedicine in 2018 is to fix the signalling/drug-bearing NPs exclusively and strongly onto their target cells located deep in a tissue which itself is located deep in the body.

**Fig. 26:**
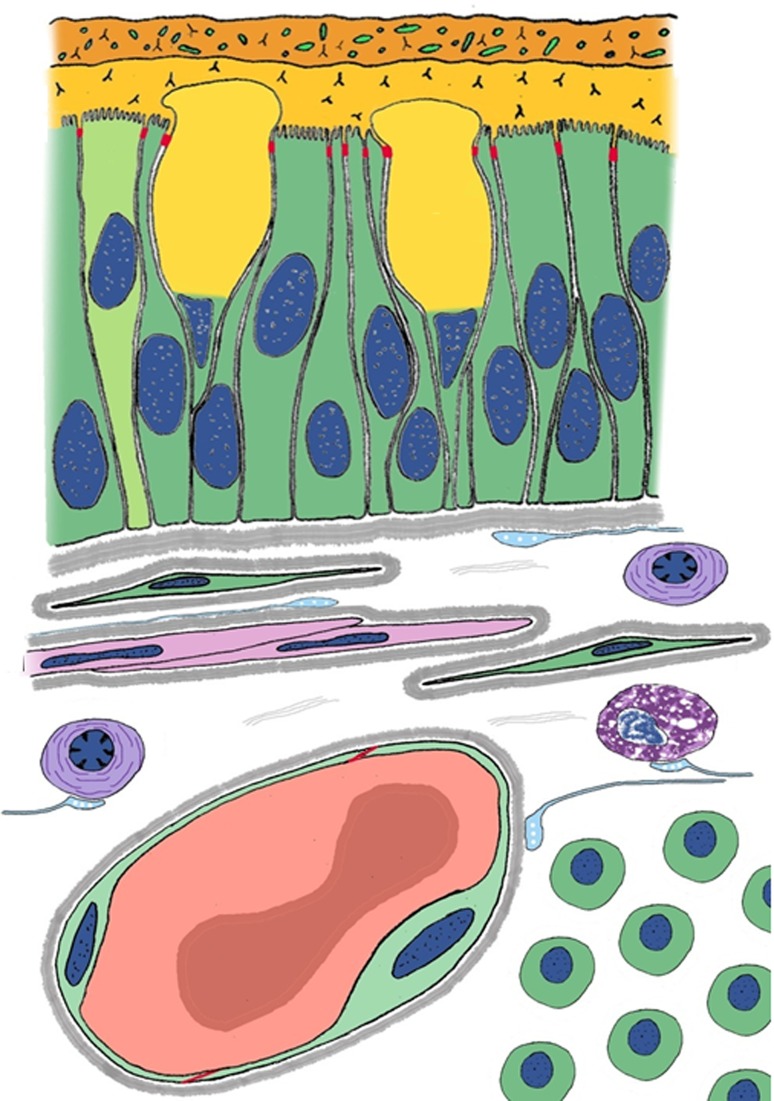
Simplified sketch drawing of a site in the gut wall. The tissue barriers between the blood and the apical surface of a transformed enterocyte (pale green) are shown in the sketch. To provide the reader with the opportunity to identify as many blood–tissue barriers as she can, we have removed the identifying labels from the tissue components in the sketch

We published the nature of that challenge earlier, and in adapted form we show here (Fig. 26) one of our sketch figures from that paper. We enquire of our reader: “How many crucially important barriers to the passage of NPs can you count in this image? (Imagine your starting point is directly beside the erythrocyte and your “destination” is the apical surface of one of the enterocytes). We ourselves are aware of at least 13 barriers barring any direct path in this tiny piece of tissue.

There is a failure to recognize the body as a complex organized self-protecting mechanism. One of us (PD) several years ago at an international meeting discussed with the departmental head of research at a multinational industrial company, who confessed his belief that “there is only one barrier in the body (the blood–brain barrier)”. If you, gentle reader, have identified the numerous barriers we carefully sketched in Fig. 26, then you will be aware of the numerous barriers that are not cellular but are built into the connective tissue (amongst other places).

**Fig. 27:**
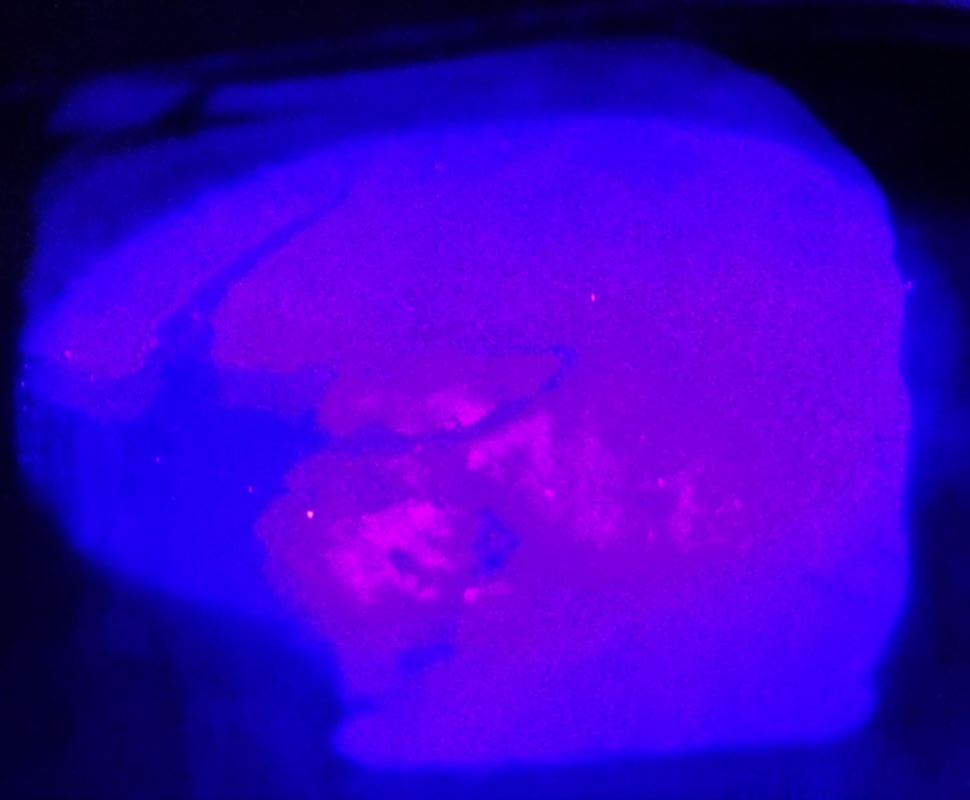
Molecular imaging of human colon cancer cells by use of well-characterized and well-standardized nanoparticles prepared by the authors of this review. The 2 µm section contained numerous nests of tumour cells which were visualized by use of targeted nanoparticles. The blue areas reflect the excitation light; the section reflects most of the blue light. The lilac areas mark the tumour cells due to the attachment of the nanoparticles. The image was captured with a simple commercial digital camera attached to a macro-objective. A 1/30 s exposure sufficed to capture this image. The optical arrangement used here has many similarities to commercial colonoscopes, and the image demonstrates that video imaging of tumours during screening endoscopy will be possible. The next step in this research is no longer research: it is development and, therefore, falls into the translational valley with its difficult landscape

Thus in Nanomedicine the central issue that is being avoided (evaded?) is Targeting. We noted this above, that there is a widespread feeling that “if you attach an antibody it will find its way”. Lamentably, this is simply entirely untrue. One of us (PD) at a European COST meeting a few years ago dubbed this “the problem of the final 10 micrometres”. The human body has been engaged in battle for many generations with agents capable of using highly sophisticated targeting strategies (see the section on viruses, above) and has developed numerous and effective barriers to control access by such agents. If each cell in the body has a single barrier, there will be > 10^12^ barriers in a human body. In fact, each cell operates numerous barriers. It will need some years of coordinated and targeted research to understand how the body’s barriers function and, indeed, where they all are. Without this research effort, Nanomedicine will remain limited to what can be achieved by “EPR”, which is certain to miss, for example, any metastatic cells that have crept behind a blood–tissue barrier. This point is of the highest possible relevance to those of us who harbour metastatic cancer. Since this issue will require some years to ripen, especially in view of the fact that at present we hardly know where the correct questions are, our target public in this review must be young researchers, e.g., those we addressed in Fig. 1. Here is a Challenge fit for a Giant (Fig. 27).

### Regulation of the clinical market

Considering the challenges and the many unknowns still present in nanomedicine it is not surprising that by 2016 only 51 nanomedical products have been approved by the FDA and EMA (Bobo et al. 2016), most of them being liposomes or polymers using EPR for tissue “targeting” (Fig. 24). Firms that have been hyped at their beginnings had to declare bankruptcy within a few years of their existence, because they did not manage to develop a hit nanoparticle (Ledford 2016). One major drawback is the usefulness of animal models in testing nanoparticle uptake into tumours: nanoparticles that show efficiency in animal models do not show the same efficiency in humans. The reason for this discrepancy is that rodents show a much faster tumour growth than humans do. This implies that the vascular structure in rodent tumours is not well-defined and leaky blood vessels are omnipresent; EPR works in rodents but not in humans. Another drawback is the complexity of many nanoparticulate systems. However, upscalability needs simple preparation processes. Following Prestwich´s advice for tissue engineering, we should “embrace complexity, engineer versatility, and deliver simplicity” (Prestwich 2008). The demands made on nanoparticles for clinical application are high. They need to be non-toxic, biocompatible, uniform in their size, stable in vivo, highly standardized, highly reproducible with marginal batch-to-batch variability, easy to upscale and easy and cost-effective in their production. The simpler the nanoparticulate formulation, the easier it is to identify its in vivo pharmacokinetics, and the easier it is to upscale it to industrial production. Clinical translation additionally requires extensive toxicological studies, which need to be fully conducted for each single type of nanoparticle; there is no standard toxicity test yet available, and very likely will never be. The availability of many different types of nanoparticles renders it extremely difficult to identify a standard way of testing. Each matrix material, each size, each functionalization which is attached to the nanoparticle can cause severe toxicities. In addition, we are only slowly beginning to understand the behaviour of nanoparticles at the nano-bio-interface; however, there is no common rule of behaviour visible. Each type of nanoparticle shows its own characteristics, and a generalised translation of these characteristics into the human biology cannot be done. Inter- and intra-patient variabilities in biological processes will render the development of a universally applicable nanoparticle very difficult. However, nanomedicine claims to be the clue for personalized therapy; it can still be realized if patients are carefully pre-examined via, e.g., imaging and biological methods identifying tumour location and tumour-specific receptors and enzymes, respectively. Nanoparticles in turn can be prepared in a simple, stable and storable manner, which would only require the respective patient-personalized targeting groups to be attached in the form of kit-chemistry. The authors have shown a way how to approach that (data not published). A pharmaceutical chemist working in the clinic has the training and the options to prepare such patient-personalized nanoparticles in-house. Regulatory agencies such as FDA and EMA so far have only approved non-targeted nanoparticles, the last being approved in 2015 (Fig. 25). This fact of course leads to the question if nanomedicine is a dying field? The answer here is “no”. A lot of research and enthusiasm is still present in the laboratories working on nanotechnology for medical applications. However, it is time for changing the focus from inventing ever new nanoparticle materials to really understanding in vivo interactions of the present nanoparticles with tissues and receptors. It is time that pharmaceutical chemists and chemical engineers cooperate with biologists and physicists possibly creating machines which are able to follow nanoparticles deep into the living body visualizing their behaviour at the nanoscale, one might speak of an “in vivo real time detection electron microscope”.

The tempo and style of this review alters in this Part. In earlier parts our topic was the interaction between energy and matter, between glowing nanoparticles and the human body. Until a project reaches technical success the number of participants is limited and can often be outsourced to this or that other colleague in the university or a nearby clinic. Once the innovative part of the creative process has produced success, there follows an equally long work period in which “bread-and-butter” laboratory work must take over, and which delivers no spectacular bibliometric “beans” and during which the employer may prohibit publication of what are increasingly valuable “Intellectual Property Rights” (IPRs). Here too the number of participants and potential beneficiaries in and of the work increases mightily and even the number of different types of participants quadruples (at least). In the commercial language so prominent in recent decades in research and development (R & D) there are different kinds of “stakeholders” who have quite varying agendas and these are often enough in conflict with those of one or more other types of stakeholder.

Every organization which does not subsist from profits feels an institutional need to present itself publicly; the urge to publish is as heavy in the young official as in the young academic. The larger the organizations, the glossier and more professionally prepared are their offerings, and the citizen is accustomed to read positive presentation and smooth linguistic self-encomiums from a multitude of organizations, also in nanomedicine. The European and national funders are prolific in this area (we cannot speak for other parts of the world). A brief internet search will turn up a plethora of reports from large funders, large and middle-sized firms and from universities. The average citizen perusing these materials will be confronted with glossy faces and smiles, often in teams, and only by careful and partially informed scrutiny will she notice the problem areas that are camouflaged in ritualized euphemisms; the art of detecting such moments in many-paged folia is one that should be learned by the young researcher and practiced until perfect. The Reports with stratospheric views are represented in this review by a funder, the European Union, that has behaved with decency and humanity in our own personal experience. At lower and less mighty levels it is wise to bear in mind that these amiable propensities are not written into the average charter of a university or of a national funding agency. They are also not categories in international business law. Quite other constraints are in play there and reflect not only the aims of the particular stakeholder but also highly personal and private motivations arising within a rather small number of people who have many and rather smaller powers to wield and decisions to make.

The level of “organization” from which glossy Reports rarely arise is the lowest. The scientist laboring on his project and mindful of his superiors does not often take time off work to prepare the personal report of his experiences, nor those of his postdoctoral colleague. When such reports do appear, in this or that media interview, they invariably focus on the successes in the work and not on the possibly malign influences of other “stakeholders”. The interlaboratory feud is rarely elaborated for public consumption. Here, therefore, we present our Report as representatives of successful researchers in the technical area, trying to find their way home in a culture of many and other agendas that cannot but conflict with those of the ground soldier working in the lab. We will complement our own personal report by noting a report from the upper eminence level of nanomedicine (Chang et al. 2015) and also a report from a European coordinating project which is glossier and heavier on positive statements although not silent on failure areas which are large enough to be noticed even from a stratospheric viewpoint. First then, we share personal experiences which begin with technical success in the project (young scientists should pay careful attention to that initiating timepoint of adversity: it follows on from the earlier experiences of finding a secure and stable job and of securing precarious and short-term funding. Next then, our personal report.

Having acquired a European grant, we and our international consortial team found a number of quite different avenues to technical success. We note this in the context of imaging colon carcinomas in a screening clinical setting, the work being aimed at developing imaging nanoparticles avoiding the usual intravenous portal of application and thus evading the numerous and highly developed blood–tissue barriers omnipresent in the human body (Fig. 26). The reader is invited to count the number of such barriers she can identify in that sketch diagram; the spatial constraints of such a sketch prevented us from including the vast majority of such barriers that are in fact present. We count the (ominous) number of 13 readily identified barriers in that sketch. Having achieved success in the technical aspects of that project (Fig. 27) we entered upon the translational aspect of our work with confidence and hope. The reader will notice that Fig. 27 is the final figure in this review. At that point the simple problems associated with scientific research had been solved and detailed and laborious testing of the techniques we had been developing became necessary—poorly paid work in terms of results and biobliometric “beans”. Here too we encountered the multitude of various stakeholders and diverging agendas that make translational work into a labyrinth ensconced in a dark valley (we have identified several “Jub–Jub Birds” by now). Unavoidably we will speak here in part as the voice of the Advocatus Diaboli. Our own experience is worth recounting here, because it is salutary and may help young researchers move amongst the pitfalls so liberally strewn around this landscape. We share here a simple list of adversities you may face as a young researcher if you move on to try applying your nostrum to the human condition. After winning a national translational prize we were trained and groomed by national translational agencies and by the European Translational Advisory Board (TAB). We approached several industrial firms at various size levels from regional to major global. We approached national funding agencies. There was no support anywhere for further development of a proven concept aimed at a major disease (Fig. 27).

Both the universities and the funding agencies face difficulties in picking up what the firms do not want to develop. Their constraints will be discussed below.

We experienced:

- bibliometrics-based evaluation penalizing innovative academics who cannot publish work that is on hold for patenting reasons;
- universities lacking a translational partnering scheme for innovators;
- universities with no Translational Research Office or a “center” or department with a comparable function.
- universities that release only part of the IPRs to the inventors, retaining strategic IPRs in the hope of realising income from them—at the risk of rendering all the IPRs unusable and unsaleable;
- failure to understand that although innovators may benefit from training in market lore and business skills, this benefit will only accrue if they are partnered and funded in their developmental work;
- quasi-official funding agencies focussed exclusively either on novel data or exclusively on industry-partnered developmental work, with neither attending seriously to early developmental research;
- funding arrangements that would not have supported Galileo, Newton, Faraday, Darwin, Einstein, Curie (until after her first Nobel prize), Schrödinger, or Göppert-Meyer in planning and carrying out their epochal research (we must confess that here is one single adversity we did not meet personally);
- regional funding agencies without interagency coordination or even contact with one another;
- short-term funding arrangements that consume 50% of research and development time by requiring repeated lengthy detailed applications for programs; with only < 10% funding rates;
- global player firms shown publicly to be focussed on minimizing their tax burdens by manipulations in the global finance markets and not on nurturing the early development of innovations;
- middle-sized firms that are too small to carry the risk of developmental financing;

We move forward from our personal experiences and survey the larger translational landscape. First we note the steep cost increases involved in moving from a laboratory-based technical success into development of that success in the translational landscape (Fig. 23). We pause to note the rather stagnating numbers of licensed nanoparticles and the heavy preponderance of liposomal formulations amongst those nanoparticles (Figs. 24, 25). To formulate better the issues involved in translational research we identify the “stakeholders” involved in research, development and translation into the clinic, they include the “ground soldiers” at the laboratory bench, the employers of those ground soldiers, the universities; other employers of the ground soldiers, both middle-sized and larger private industrial firms; clinical bodies with major objectives to organize the care of patients; the patients; the semipublic agencies disbursing research funding, and finally the public authorities charged with maintaining drug safety as uppermost priority.

Our first stop is now to examine a historical episode which points up some important aspects of the translational valley, one in which premature developmental work generated costs and losses that were appreciable even from the viewpoint of global player firms. The public scrutiny of the episode was limited to a relatively small number of journalistic publications appearing in the scientific journals, far fewer than, for example, the Rolling Stones would harvest in a wider spread of publications. We repeat largely verbatim what was written at the time in science journals, it needs little further comment from us, but we will note one or two matters that can be discussed profitably now, a decade after the event.

The pharmaceutical industry invested large sums in wrapping siRNA molecules into nanoparticles in the hope that the exquisite specificity of the siRNAs could be unleashed precisely at their target sites, thus generating highly active and highly specific blockbuster medicaments. After the end of the (foreseeable) siRNA failure journalistic reporting rapidly died away and the only lesson the firms admitted was “we had trouble with the targeting”. The following excerpted period pieces summarize the journalistic reporting of the siRNA episode; the excerpts are from the few journalistic reports that appeared in high-impact journals during 2010-2011, e.g., Ledford (2010). In 2010: *“Merck paid more than US$1 billion to snatch up Sirna Therapeutics in San Francisco, California*—*one of the first biotechnology companies aiming to harness RNAi to create new drugs. Yet et al. that seemed like ancient history last week when drugs and diagnostics corporation Roche in Basel, Switzerland*—*a major investor in RNAi*-*based drug research*—*announced it was killing its program after spending 3* *years and more than $500 million on the technique; the company declared it would cease developing RNAi*-*based therapeutics. The development of RNAi*-*based drugs has stalled as companies confront the challenge of delivering RNA molecules, which are notoriously fragile, to target cells in the human body, and then coaxing those cells to take up the RNA. Alnylam is laying off 25*–*30% of its staff as its long*-*standing collaboration with Novartis around RNA*-*interference*-*based therapeutics comes to a close”. Merck spokeswoman Carolyn Lappetito says her company has not pulled back from RNAi.* A year later in 2011 the following was reported: *“Less than 5* *years after betting $1.1 billion on the power of RNA*-*interference (RNAi) to treat disease, pharmaceutical powerhouse Merck is shutting down the San Francisco research facility it acquired when it purchased Sirna Therapeutics, a biotechnology firm that specialized in RNAi, in 2006”.* Although this period piece largely speaks for itself, there are one or two critiques to make here that may have value. Firstly, there was no public post-mortem of the episode, which affected several competing firms at the same time, and from which the whole research field of Nanomedicine could have learned. Secondly, the > 2 billion US$ that were lost in these abortive efforts (several other firms had similar costs and experiences in this chase after a RNAi-based blockbuster) did not issue in a learning experience for the Nanomedicine field as a whole. It is fair to point out that this was not “public money” being lost, but equally fair to add that the firms certainly recouped their losses from their customers: from the public. It is likely that the sole learners from this expensive episode were workers in the research labs—which the firms promptly shut down. Such lack of communication, therefore, surely contributes to hindering the blossoming of Nanomedicine, but will not be presented in readily digestible form in large and glossy Reports. We note here the constraints acting on the firms, because these constraints became briefly visible in the “siRNA episode”. The firm is racing against other firms to acquire blockbuster drugs that bring in large profits. The firms, therefore, do not take a long view. They are much more focused on tangible and fungible short-term successes, getting perspective by watching their competitor firms, by checking the opinions of customer physicians in clinics, by listening at large scientific/medical conferences, and by hearing guidance from the Marketing Manager. It is fair to point out that the ire of the firm’s stockholders can well reach down to the level of the marketing manager, and his influence then reaches down to the more obscure nooks of the firm. These circumstances lead to research directed mainly by non-scientists, and thus comparable to the official-directed research supported by the funding agencies, though much shorter term in outlook.

Industrial firms in the heavily regulated biomedical markets are also constrained by the opinions of the official regulators of those markets. Where the regulators are to some extent free of political direction there will appear another and very human aspect of their work. The individual decision makers in such regulatory authorities may be willing to share thoughts and wisdom with researchers and firms, but they remember vividly that in 2 years time the products from those researchers and firms may land on their desk and be the object of perceived Conflict of Interest. Our experience with this was most impressive, and it was a major loss of input to our project and work that this contact could not be repeated as we proceeded with the technical work.

We move forward and down to the national funding agencies and the European funders. Most noticeable about the funding agencies is their focus on “excellence and innovation of research”, which are most easily measured by bibliometric indices. This traps the young researcher and also the entire field of developmental research in the dilemma: whether to publish and thus lose the IPRs (the normal decision made by researchers) or to aim for acquiring IPRs but to gainsay the chance of early publication and thus to damage one’s career. We experienced this first-hand, as do many others. The other aspect of the funding agencies is their role in the living out of personal feuds and private agendas. This is more conspicuous in the national and regional agencies and barely noticeable (if at all) at the level of the major European agencies. The difficulties thus visible in the national agencies can be avoided by addressing them through the European agencies, as we experienced personally.

We move further forward and down to the university level. We have discussed experiences at this level above, but it is valuable to identify the constraints acting upon university decision makers. The first is actuarial: if the university owns “your” IPRs it may well be able to license them out for profit and few universities are in a position to ignore this. The withholding of some part of the IPRs is dangerous for the work as a whole but may pay handsomely if the university can realize upon them. Furthermore, the university may find someone cheaper to hire than the original innovator, but also capable of carrying out developmental work, and it will do this if at all possible. The critique above of national funding agencies as feuding grounds of course applies much more intensely to the universities, where almost everybody knows almost everybody else. It seems that without an established cultural ethic of supporting developmental work the universities will look first to their potential profit from IPRs.

We note finally the limited and usually late involvement of clinical bodies and of patients in translational development. This usually happens after journalists have processed the topic in regional or national newspapers; it does not normally follow upon reception of the reports issued by universities or of the Reports issued by the large organizations.

Having provided this our own eye-witness report on the interplay between these several stakeholders we complement our own evidences with those from far more August bodies.

### Consider the penetrating and authoritative analysis given by Chang et al. (2015), which in 2015 pointed to

- USD 22 billion budgeted by the USA National Nanotechnology Initiative between 2000 and 2016;
- 650 Million Euros invested by the European Commission under the Framework research program FP7;
- 22 approved nanomedicines in Europe (22 approved by the European regulator and 71–87 by the FDA in the USA;
- 123 clinical trials running in 2015 with nanotherapeutics;
- insufficient attention being given to the industrial process, for example, during the education and training of the next generation of scientists.

In Europe, deep-seated problems in the culture which produces outstanding research scientists but very few young graduates who know what the industrial requirements are for both R&D: Europe does not train adequately for the developmental and translational sciences. Note that this authoritative summary concords with our views and experiences, though we have couched our version in pithier terms.

We complete this brief survey at different levels with the Report issued by the European Commission: (https://cordis.europa.eu/result/rcn/153608_en.html). In a prose rather turgid for a layman to read it points amongst many positive statements also to the aspects in which the European nanomedicine research enterprises might benefit from better structures. Thus it notes the following:

- the need to federate the nanomedicine community;
- the benefits of networking between nanomedicine communities;
- the importance of communication between nanomedicine researchers and the public;
- the lack of dedicated strategies for nanomedicine in 22 European countries;
- 28% of the countries have legal regulations hindering them in participating in joint transnational calls.

We ourselves experienced the failure of a major project initiative which we coordinated in Europe: at a late stage of the application the local national regulators forbade the participation of a willing global player firm, because that firm was not based in the country: this against the background of only 5 firms worldwide operating in this field of work and none of them based in the country involved. Under the rules of the Call only projects with “partner” firms could apply, and so our project died. The Report states that “knowing both the barriers and opportunities for commercialisation are hence very important even at a basic research stage, and overcoming these barriers would significantly challenge the status-quo for many academics and SMEs (SMEs are small and medium-sized firms)”.

The Report also notes that there are three major barriers in early clinical development: these include the difficulty to find appropriate funding to finance the proof of concept to further raise investment from venture capitalists; they note that this has slowed down the development of the product.

We end the chronicle of woes with a summary: the financial costs named in this review include the USD 22 billion budgeted by the USA National Nanotechnology Initiative between 2000 and 2016, the 650 Million Euros budgeted in Europe under the FP7 program, and the approximately 2 billion USD reported by industrial firms as spent in unsuccessful attempts to couple the advantages of nucleotide medicine with nanomedicine between 2005 and 2012. The approximately 25 billion dollars noted here do not include resources invested by other regions than the USA and Europe. Chinese investment in nanomedicine is high, as evidenced by the numerous Chinese authors cited in this review. It seems that a rough total global investment in nanomedicine would add up to a sum in the region of 50 billion US dollars. From these invested resources the harvest of clinically licensed nanomedical products totals in 2018 is not much better than approximately 100 products. Thus in a very crude reckoning, each nanomedical product in clinical use has cost on average approximately a half billion dollars. The yield per investment in nanomedicine reminds these authors of the drug efficiency of small-molecule drugs, which is approximately 0.01%. If one ignores the costs of misguided efforts by industry to create blockbuster drugs in premature development efforts, there remain two evident culprits in explaining the inefficiency in moving nanoparticle research forward into clinics. The first is the intrinsic difficulty of delivering large molecules or nanoparticles to target sites in the human body, which we identify above as a major research challenge today in nanomedicine. The second is the disjointed nature of the interfaces between academic experts and both private and public bodies appearing as stakeholders in the development process. In particular the heavily regulated nature of any clinical application renders development of a nanomedical product far more expensive than its academic-based initial design and development. Numerous project funding opportunities are available for the relatively cheap academic research, but funding for the subsequent development is subject to the larger dimension of the funding required. Exacerbating this is the reluctance of national authorities to share burdens and profits with other countries, the blockbuster-based culture of the global industrial players, and friction between university authorities and potential developmental funding sources due to inappropriate proprietary behaviour by the universities. Our own TAB assistance lies on ice, because the university involved retains a portion of the IPRs in our work, but without these IPRs no negotiations with developmental funders is possible.

We leave this brief overview of the sad developmental research landscape, in which both the two higher reports presented above agree with our low viewpoint from the ground, and we end this review by offering a few words of advice to young scientists, and then pointing them to some of the glimmers of hope that are starting to grow amongst the desolation.

Our advice to young researchers:

Check carefully on the university you are envisaging as a research base. Does it have a culture of partnering its innovators? Does it run a translational office? Does it engage with significant resources in collaborative research and development with the nearby clinics (ask the clinicians, not the university). Does it cooperate with regional and local politicians to provide facilities such as incubators for innovations? (ask journalists, not the politicians or the university). Does it have a clearly and simply stated career plan supervised by close discussion with its (equivalent of an) employees’ council? Check with the employees’ council, not the university. Distrust the university until it has shown good intentions by providing significant funds and infrastructure for developmental work. If your checks turn up a “No” to more than one or two of these checks, we recommend that you focus on identifying a more cooperative university at an early stage of your career. Finally, in Table 5, we point to some future hopes.

**Table 5:** Organizations regulating and coordinating nanomedical translational work

EMA	European Medicines Agency	https://www.ema.europa.eu/
ETPN	European Technology Platform for Nanomedicine: established the first European Translational Advisory Board	https://etp-nanomedicine.eu/
ERC	European Research Council	https://erc.europa.eu/
FDA	US Food & Drug Administration	https://www.fda.gov/
NNI	National Nanotechnology Initiative	https://www.nano.gov/
Health Canada	Health Canada	https://www.canada.ca/en/health-canada.html
NINT	National Institute for Nanotechnology	http://www.nint-innt.ca/
PMDA	Pharmaceuticals and Medical Devices Agency	https://www.pmda.go.jp/english/
JST	Japan Science and Technology Agency	https://www.jst.go.jp/EN/
TGA	Therapeutic Goods Administration; Australian Government Departmen of Health	https://www.tga.gov.au/
ANN	Australian Nanotechnology Network	http://www.ausnano.net/
CFDA	China Food and Drug Administration	http://eng.sfda.gov.cn/
NanoCTR	National Center for Nanoscience and Technology	http://english.nanoctr.cas.cn/

## Summary

We have saluted the Giants, men and women who were great not only in Physics but also in Nanotechnology, and we also salute hopeful Giants of the future.

We have rehearsed in simplified form some of the quantum–mechanical concepts relevant to nanotechnology, together with brief histories of these topics.

We have presented some “quantum nanoparticles” which generate photoluminescence from the nanoparticle matrix itself, considered the mechanisms causing their glow, and given brief synopses of their histories. We focussed attention on nanoparticles based on the allotropes of carbon, with their expected low toxicities.

We have considered the quantitation of optical pathways especially in the infrared spectrum and the utility of such quantitation in the rational design of nanoparticles for use in clinical diagnostics and monitoring.

We have noted an early report describing nanoparticle-mediated local disruption of one type of barrier at the cellular level.

We have extended our previous thoughts on targeting of nanoparticles and once again identified the challenge of targeting nanoparticles to their pre-ordained sites in the human body as the urgent and major challenge today in nanomedicine. We presented one of numerous cases in which targeting failure destroyed large hopes.

We have shown data indicating a slackening in the pace at which nanoparticle-based clinical applications are being licensed for sale and use in the clinic.

We have considered the “hostile environment” of the translational landscape and—based on our personal experience—have identified some of the issues that house there and have offered warnings and advice intended to guide today’s young Giants in navigating the pitfalls there.

We provide a glimmer of hope that some dwarf beginnings can be discerned to encourage and aid wanderers in the dismal translational landscape.

## Conclusions

Nanomedicine has a bright future if it can move on from the double whammy of precarious targeting and precarious financing; that future would see: Major research attention migrating from creating novel nanoparticle chemistries to a focus on solving targeting issues.

There are useful organisational developments like fireflies glowing in the gloom of the translational valley: these offer hope of a brighter future. However, in view of our own experience a more ample structure needs to be set up: the translational phase of development needs to be stimulated and bolstered by funding which has no actuarial connection with market-based income and profitability.
